# Precision-Engineered Dermatan Sulfate-Mimetic Glycopolymers for Multi-Targeted SARS-CoV-2 Inhibition †

**DOI:** 10.3390/md23120486

**Published:** 2025-12-18

**Authors:** Lihao Wang, Lei Gao, Chendong Yang, Mengfei Yin, Jiqin Sun, Luyao Yang, Chanjuan Liu, Simon F. R. Hinkley, Guangli Yu, Chao Cai

**Affiliations:** 1 Key Laboratory of Marine Drugs of Ministry of Education, Shandong Key Laboratory of Glycoscience and Glycotherapeutics, School of Medicine and Pharmacy, Ocean University of China, Qingdao 266003, China; leowang@stu.ouc.edu.cn (L.W.); gaolei2@msu.edu (L.G.); sunzetao@hotmail.com (C.Y.); ymf@stu.ouc.edu.cn (M.Y.); 13262540703@163.com (J.S.); 2119083118@stu.ouc.edu.cn (L.Y.); liuchanjuan@ouc.edu.cn (C.L.); 2 The Ferrier Research Institute, Victoria University of Wellington, 69 Gracefield Road, Lower Hutt 5040, New Zealand; simon.hinkley@vuw.ac.nz; 3 Laboratory for Marine Drugs and Bioproducts, Qingdao Marine Science and Technology Center, Qingdao 266003, China

**Keywords:** dermatan sulfate, synthesis, disaccharide, glycopolymer, anti-SARS-CoV-2

## Abstract

The ongoing COVID-19 pandemic, caused by SARS-CoV-2, continues to pose major global health challenges despite extensive vaccination efforts. Variant escape, waning immunity, and reduced vaccine efficacy in immunocompromised populations underscore the urgent need for complementary antiviral therapeutics. Here, we report the design, synthesis, and biological evaluation of precision-engineered dermatan sulfate (DS)-mimetic glycopolymers as multi-targeted inhibitors of SARS-CoV-2. Guided by molecular docking and virtual screening, sulfation at the C2 and C4 positions of iduronic acid was identified as critical for binding to the viral spike protein and inhibiting host and viral enzymes, including heparanase (HPSE) and main protease (M^pro^). Chemically synthesized DS disaccharides were covalently grafted onto polymer scaffolds via a post-modification strategy, yielding glycopolymers with well-defined assembly that form uniform nanoparticles under physiological conditions. Surface plasmon resonance and pseudovirus assays revealed strong binding to the viral spike protein (K*_D_* ≈ 177 nM), potent viral neutralization, and minimal cytotoxicity. Cellular uptake studies further demonstrated efficient internalization of nanoparticles and intracellular inhibition of HPSE and M^pro^. These results establish a modular, non-anticoagulant, and glycosaminoglycan-mimetic platform for the development of broad-spectrum antiviral agents to complement vaccination and enhance preparedness against emerging coronavirus variants.

## 1. Introduction

COVID-19, caused by severe acute respiratory syndrome-related coronavirus 2 (SARS-CoV-2), remains a persistent global health challenge. The World Health Organization reports over 7.1 million deaths worldwide [1,2,3], reflecting continued transmission and mortality despite extensive public health interventions. Successive viral mutations have sustained infection rates and complicated containment efforts [4,5,6]. Although approved vaccines have markedly reduced severe illness and mortality, their protection against infection and mild disease has diminished over time [7,8]. Antigenic drift among emerging variants further erodes vaccine efficacy, underscoring the limitations of vaccination alone for long-term viral control [9].

Similarly to the 2002 SARS-CoV strain, SARS-CoV-2 invades host cells through the binding of its surface spike protein to the angiotensin-converting enzyme 2 (ACE2) receptor [10,11,12]. In both viruses, the main protease (M^pro^) is essential for viral replication. Beyond processing viral polyproteins, M^pro^ can also cleave host proteins, thereby creating conditions that favor infection and viral proliferation [13]. These parallels suggest a common therapeutic vulnerability. Targeting the spike protein–ACE2 interaction, together with the inhibition of M^pro^ activity, offers a promising strategy for developing broad-spectrum inhibitors capable of preventing future severe acute respiratory syndrome outbreaks [14,15,16].

Glycosaminoglycans (GAGs) are linear sulfated polysaccharides composed of repeating disaccharide units of hexosamine and uronic acid. Based on their disaccharide structures, GAGs are classified into heparan sulfate (HS), chondroitin sulfate (CS), heparin (HP), and keratan sulfate (KS), among others [17]. Since 2020, numerous studies have shown that HS contributes to SARS-CoV-2 infection. This effect is largely attributed to the ability of the spike receptor-binding domain (RBD) to bind HS in a manner that depends on both chain length and specific sequence features. HS appears to serve as an initial attachment site, enabling the virus to move through the glycocalyx via weak individual contacts that collectively produce strong overall binding. This positioning helps bring the virus to the cell surface, where it can subsequently engage ACE2 and initiate entry [18,19,20,21]. Building on this understanding, the Linhardt and Pomin groups jointly screened a series of marine-derived sulfated polysaccharides for their ability to inhibit infection by multiple SARS-CoV-2 variants in vitro [20,22,23]. Their results spurred a broader investigation into sulfated polysaccharides and their synthetic analogs as potential antiviral agents against COVID-19 [24]. Follow-up studies revealed that the selective desulfation of heparin alters its antiviral activity in distinct manners; specifically, *N*-desulfation markedly reduced antiviral activity against SARS-CoV compared to 6-*O*-desulfation. These findings underscore the critical role of sulfate patterning on cell-surface GAGs in determining their antiviral function [25].

While HS and HP have been extensively studied [26], dermatan sulfate (DS)—a structurally related glycosaminoglycan—remains comparatively underexplored. DS is composed of repeating GalNAc-β-(1→3)-IdoA-α-(1→4) disaccharide units and often occurs as copolymeric chains with chondroitin sulfate (CS). Variants of CS and DS were first identified in marine organisms, underscoring their structural diversity and potential biological significance [27,28]. Endogenous CS/DS contributes to cell proliferation, migration, neuronal differentiation, skeletal development, and immune regulation [29,30].

Beyond the spike protein and M^pro^, heparanase (HPSE), an endo-β-*D*-glucuronidase [31], has also been implicated in SARS-CoV-2 infection [32]. HPSE commonly cleaves the heparan sulfate (HS) side chains of heparan sulfate proteoglycans (HSPGs), thereby diminishing viral adhesion to host cells and facilitating viral dissemination [33]. Notably, HPSE expression is markedly upregulated in cells infected with SARS-CoV-2. In contrast, HPSE-deficient macrophages show reduced viral load and inflammation, underscoring the enzyme’s dual role in viral propagation and immune modulation [34]. Synthetic HPSE inhibitors that are structurally related to heparin, such as Roneparstat and PG545 (Pixatimod), have gained attention for their ability to target both the spike protein and HPSE, offering a promising multi-target antiviral strategy [35].

**Scheme 1:**
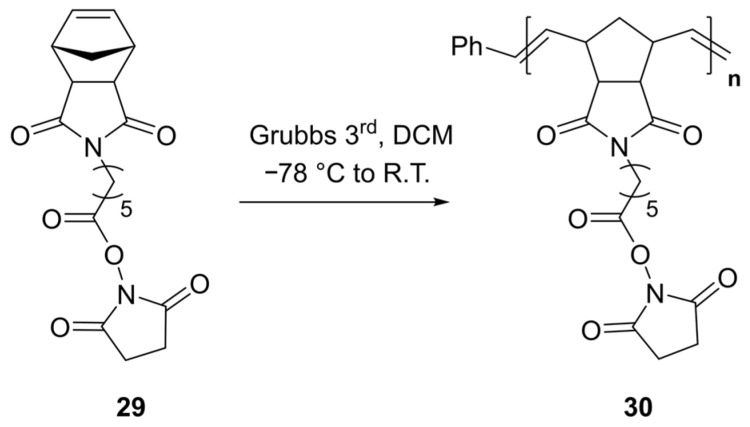
Synthesis of polymer backbone **30**.

DS functions as a non-anticoagulant inhibitor of HPSE and offers lower side effects with improved safety, making it a promising candidate for anti–COVID-19 therapy. While HS plays a crucial role in mediating spike protein binding and viral entry, its interactions with a broad range of binding partners present a challenge. In designing biomimetic glycopolymers targeting HS, it is vital to focus on the selectivity of HS mimetics for specific binding proteins, particularly those like PF4 [36]. Non-selective binding to such proteins could result in undesirable side effects, such as thrombocytopenia. In contrast, the structural differences between DS and HS offer a potential advantage by reducing the likelihood of these side effects, making DS a safer alternative for targeting viral entry. However, natural DS suffers from inherent limitations, including heterogeneous molecular weights and impurities arising from variable site-specific sulfation patterns within chondroitin sulfate. To address these challenges, glycopolymer-based analogs inspired by natural polysaccharides have emerged as a promising alternative. The Hagg group demonstrated that negatively charged polysulfates can mimic natural HS and inhibit SARS-CoV-2 by interacting electrostatically with the spike protein [37]. Concurrently, the Nguyen group designed HS mimetics that have low affinity for heparin-binding proteins, such as PF4, thereby reducing potential side effects and enhancing the safety profile of antiviral glycopolymers [38]. Such synthetic glycopolymers enable precise control over structural features, yielding drug candidates with defined active motifs, enhanced uniformity, reduced toxicity, and improved efficacy [35]. Our laboratory previously developed a ‘post-modification’ strategy based on ring-opening metathesis polymerization (ROMP), enabling the covalent grafting of a synthetic regio-selectively sulphated galactosamine derivative onto a polymer backbone (Scheme 1) to generate multivalent glycopolymers with tunable bioactivity [39].

Here we report the design, synthesis, and in vitro antiviral evaluation of DS-mimetic glycopolymers. The functional disaccharide motifs were identified through molecular docking and virtual screening of selectively sulfated structures. Distinct sulfation patterns produced clear differences in binding affinity for the SARS-CoV-2 (2019-nCoV, Wuhan variant) spike protein and inhibitory activity toward M^pro^ and HPSE enzymes. These findings provide valuable insight for developing non-anticoagulant, glycosaminoglycan (GAG)-mimetic antivirals with broad therapeutic potential.

## 2. Results

### 2.1. Rational Screening, Design, and Synthesis of DS-Mimetic Disaccharide Modules

To evaluate the antiviral potential of GAG polysaccharides against SARS-CoV-2, we first performed molecular modeling and docking of representative GAG disaccharide units. Three-dimensional structures were generated using GLYCAM [40], and docking simulations were carried out with Vina-Carb [41]. The resulting scores are summarized in Table S1.

CS/DS were selected for further analysis due to their shared highly similar structural motifs to heparin and their comparatively lower incidence of reported side effects [42,43]. Docking results showed that disaccharides derived from CS and DS bind heparanase (HPSE) less strongly than those derived from heparan sulfate (HS). This weaker interaction suggests a reduced likelihood of off-target recognition by HS-binding proteins, thereby potentially minimizing unintended biological responses [28,38]. Within these two families, DS disaccharides present a clearer structure–activity trend [44]. Selective sulfation on DS units produced more distinct changes in predicted activity than comparable modifications on CS units, indicating that DS offers a more tunable platform for rational design. Moreover, the docking analysis of the six HS/HP disaccharide units (Table S1, entries 2–7) showed a clear trend: units containing IdoA (entries 5–7) had substantially higher binding scores across all three proteins than those glycopolymers containing GlcA (entries 2–4). This aligns with prior reports highlighting the key contribution of IdoA residues to GAG–protein interactions [45]. On this basis, we focused subsequent screening on DS disaccharides that incorporate IdoA units.

After visualizing the docking poses in 3D and evaluating the forces involved in a 2D diagram (Figures S1–S6), we observed that the addition of sulfate groups strengthens the DS disaccharide’s binding within the RBD pocket. In particular, sulfation improves electrostatic attraction to the positively charged residue K403 and increases stabilizing contacts, including more hydrogen bonds (e.g., with K453 and K495) and additional hydrophobic interactions (e.g., with K505). The location of sulfation also mattered. Notably, sulfation at the C2 and C4 positions of iduronic acid in DS enhanced the predicted antiviral interactions more effectively than sulfation at the C4 and C6 positions of GalNAc. A plausible explanation is that IdoA-2/4S enables deeper insertion into the binding pocket, thereby positioning the GalNAc unit closer to the critical residue K449 [18]. Consistent with this interpretation, different sulfation patterns shifted the disaccharide’s orientation in a manner that altered its contacts with K449. In **DM2**, GalNAc sulfation at C4 and C6 pulled the molecule away from K449, weakening or eliminating GalNAc–K449 interactions. In **DM3**, sulfation occurring exclusively on IdoA positioned GalNAc too close to K449 (approximately 1.42 Å), a distance potentially unfavorable for stable binding. In **DM4**, the more evenly distributed negative charge stabilized the disaccharide in the pocket and led to the strongest overall binding.

**Scheme 2:**
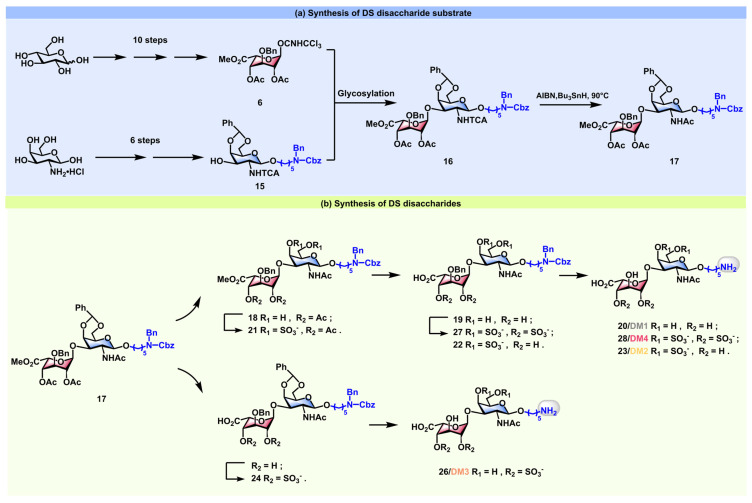
(**a**) Synthetic route of DS disaccharide substrate. (**b**) Synthetic route of DS disaccharides.

Guided by these results, we designed four DS-mimetic disaccharide models and conducted retrosynthetic analysis as shown in Scheme S1. Starting from *D*-glucose and *D*-galactosamine·HCl, the aldonic acid (IdoA) donor **6** (Scheme 2a and Scheme S2) [46] and the *N*-trichloroacetyl-galactosamine acceptor **9** (Scheme S3) [39] were prepared through multistep sequences. Glycosylation of these partners furnished the orthogonally protected disaccharide **11**. During the deprotection of **11**, an azide-bearing linker proved incompatible with the NH-TCA (2,2,2-trichloroacetyl) group, leading to incomplete deprotection (Scheme S4). We therefore replaced the reducing-end linker with alcohol **13**, protected by benzyl (Bn) and carbobenzyloxy (Cbz) groups, and revised the route to obtain the *N*-trichloroacetyl-galactosamine acceptor **15** (Scheme 2a and Scheme S5). The orthogonally protected disaccharide module **16** was synthesized with glycosylation between **6** and **15**, and DS disaccharide **17** was successfully achieved after reduction (Scheme 2a and Scheme S6).

Using disaccharide **17** as the core scaffold, four DS-mimetic disaccharides, including **DM1**, **DM2**, **DM3**, and **DM4**, were subsequently prepared through a combination of orthogonal deprotection and selective sulfation strategies (Scheme 2b and Scheme S7).

### 2.2. Preparation, Characterization, and Protein-Binding Assessment of Diverse DS-Mimetic Glycopolymers

**Figure 1:**
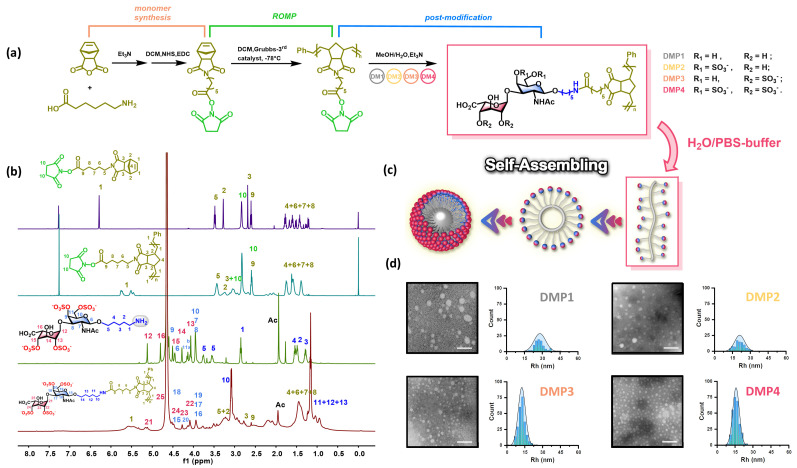
(**a**) Synthetic route of DS-mimetic glycopolymers. (**b**) Overlayed ^1^H NMR spectra of the polymer backbone (**31**) in CDCl_3_, DS-mimetic disaccharide **28** (**DM4**), and the relative glycopolymer (**DMP4**) in D_2_O. (**c**) A schematic illustration of aqueous self-assembly of the glycopolymers into nanoparticles. (**d**) A TEM image of the resulting DS-mimetic glycopolymer nanoparticles (scale bar, 100 nm).

Following the successful synthesis of four distinct DS-mimetic disaccharides, a polymer scaffold featuring *N*-hydroxysuccinimide (NHS) ester functionalities (degree of polymerization = 62) was prepared using established methods (Figure 1a) [39]. These disaccharide units were subsequently grafted onto the scaffold in a methanol/water system, yielding four novel glycopolymers (Figure 1a and Scheme S8). The successful grafting and precise degree of sugar substitution (DSS) for each glycopolymer were verified by NMR spectroscopy (Figure 1b and Table 1).

Consistent with our previous reports, these glycopolymers were observed to self-assemble into stable nanoparticles in aqueous solution, driven by the amphiphilic balance between the hydrophilic sugar side chains and the hydrophobic polymer backbone (Figure 1c). The particle size and uniformity of the resulting nanoparticles were characterized by transmission electron microscopy (TEM) and dynamic light scattering (DLS) (Figure 1d and Table 1). The glycopolymers self-assembled into nanoparticles with an average diameter of approximately 20 nm under physiological conditions (Figure 1d). Glycopolymers bearing sulfate modifications exhibited a more uniform size distribution and enhanced structural stability. Notably, glycopolymers **DMP3** and **DMP4**, which bear sulfate modifications at the C2 and C4 positions of IdoA within their disaccharide units, consistently formed smaller and more uniform nanoparticles (Table 1).

It is worth noting that the particle sizes measured by DLS were approximately 10–15 times larger than those observed by TEM. This discrepancy likely arises because during TEM analysis, the nanoparticles are well dispersed on the copper grid after solvent evaporation, whereas in DLS, strong interparticle interaction and the hydration layer around micelles increase the apparent hydrodynamic diameter (Z-average, R_h_) [47].

Relative to **DMP2**, both **DMP3** and **DMP4** exhibit more negative (higher-magnitude) *ζ*-potentials (Table 1). Notably, **DMP3** outperforms **DMP2** despite carrying the same number of anionic groups (sulfate and carboxylate). This is attributed to the greater hydrophilicity and outward presentation of the anionic substituents in **DMP3**, promoting their localization at the nanoparticle–water interface, increasing surface charge density, and stabilizing self-assembly. Although **DMP4** carries five negative charges and **DMP3** carries only three, both exhibit nearly identical zeta potentials. This observation suggests that, during self-assembly, the glycopolymer adopts a morphology in which the sugar-based side chains orient outward, while the hydrophobic backbone and reducing-end GalNAc units are sequestered inward. As a result, the hydrophilicity and surface charge are dominated by the non-reducing end IdoA2/4S moieties, whose influence on the nanoparticles far exceeds that of the GalNAc4/6S units. All these physicochemical features above suggest that **DMP3** and **DMP4** may display superior biological activity [48,49].

Molecular docking indicated stronger binding of **DM3** and **DM4** to the spike RBD than **DM2** (Table S1). For **DM4**, the predicted interactions are dominated by hydrogen bonds with polar residues and electrostatic contacts with basic residues in the RBD. By contrast, **DM1** (unsulfated) and **DM2** (GalNAc C4/C6 sulfation) showed weak RBD affinity. Upon grafting the disaccharides onto the polymer backbone, the ‘multivalent effect’ potentially promotes further-enhanced protein binding [50,51].

Consistent with these predictions, SPR measurements against spike and ACE2 (Table 2) showed that **DMP4** bound spike with the greatest affinity (K*_D_* ≈ 177 nM), approximately an order of magnitude stronger than its binding to ACE2 (K*_D_* ≈ 1088 nM). Other glycopolymers also bound spike in the several-hundred-nanomolar range, with **DMP3** outperforming **DMP2**, underscoring the positional specificity of sulfation in GAG-mimetic oligosaccharides. These trends align with the nanoparticle characterization data (Figure 1d and Table 1). We also assessed the spike-binding properties of our previously synthesized glycopolymer **MMP**, which features 3,4,6-*O*-sulfated GalNAc motifs and is known for its antitumor activity [39]. **MMP** displayed the second-highest binding affinity following DMP4, despite its simpler structure. These results suggest that **MMP** holds strong potential for further development as a broad-spectrum antiviral agent, particularly for rapid deployment during public health emergencies.

SPR analysis confirmed the reliability of molecular docking for assessing the antiviral potential of GAG oligosaccharides. The results demonstrated that, once incorporated into a glycopolymer, the DS-mimetic disaccharides exhibited high-affinity binding to the viral spike protein, underscoring the promise of sulfated glycopolymers as antiviral candidates. Based on these findings, the safety and antiviral efficacy of the synthesized glycopolymers were then evaluated.

### 2.3. In Vitro Antiviral Activity and Cellular Uptake of DS-Mimetic Glycopolymers

**Figure 2:**
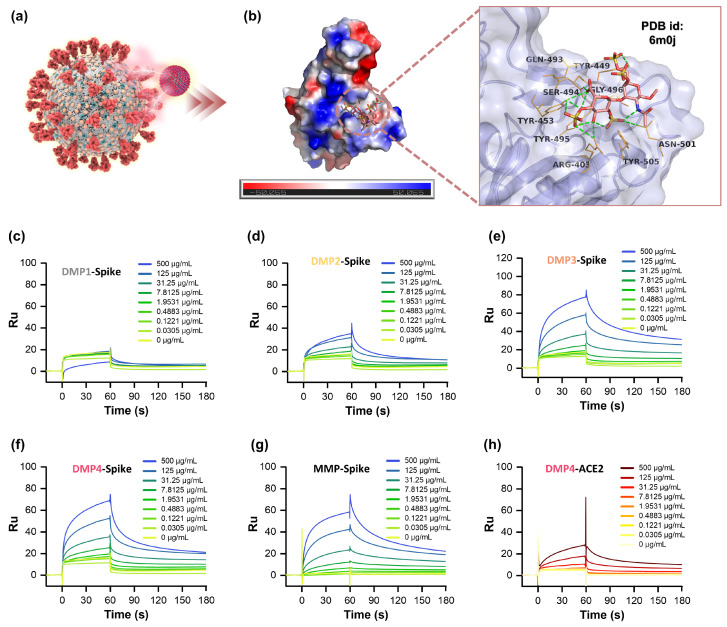
(**a**) Schematic diagram of glycopolymer nanoparticles inhibiting SARS-CoV-2. (**b**) Molecular docking of DS-mimetic disaccharides and spike protein. (**c**–**g**) SPR analysis of DS-mimetic glycopolymers with spike protein: (**c**) **DMP1**; (**d**) **DMP2**; (**e**) **DMP3**; (**f**) **DMP4**; (**g**) **MMP**. (**h**) SPR analysis of **DMP4** with ACE2 protein.

**Figure 3:**
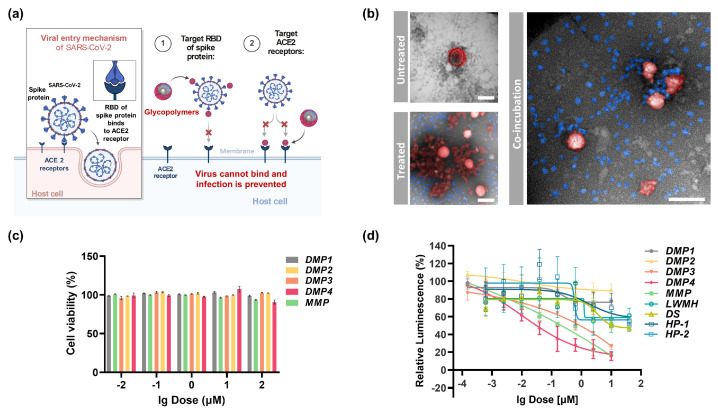
(**a**) Schematic diagram of DS-mimetic glycopolymer nanoparticles targeting viral particles and protecting host cells. (**b**) TEM images of pseudovirus before and after treatment with **DMP4**; scale bar, 100 nm; The pseudo-colors are added for visual clarity: red, pseudovirus particles and fragments; blue, glycopolymer nanoparticles. The original TEM image is provided in the Supporting Information (Figures S7–S9). Co-incubation: pseudovirus and glycopolymer were mixed and immediately processed for negative-stain TEM; Treated: samples prepared identically but incubated for 2 h at 37 °C before grid preparation. (**c**) Cytotoxicity assays of glycopolymers (mean ± SD, n = 3). (**d**) Pseudovirus infection assays of diverse sulfated glycopolymers (mean ± SD, n = 3).

Previous reported studies, including those by Linhardt and co-workers, have established that during SARS-CoV-2 infection, the viral spike protein first interacts with HS on the host cell surface, which facilitates subsequent engagement with the ACE2 receptor to trigger cellular entry [10,12,19,23,25]. The SPR analyses in this study have confirmed that DS-mimetic glycopolymers can bind both the spike protein and ACE2 (Figure 2 and Table 2). It is proposed that these glycopolymers competitively associate with the viral spike protein, thereby preventing the viral attachment to cell-surface HS, while exhibiting weaker, lower-toxicity interactions with ACE2 as well. This dual-binding behavior may effectively block the viral internalization pathway and enhance antiviral efficacy (Figure 3a).

TEM imaging revealed the morphology of SARS-CoV-2 pseudoviruses displaying surface spike proteins (highlighted in red in Figure 3b). TEM showed that **DMP4** glycopolymer nanoparticles (highlighted in blue in Figure 3b) adsorbed onto and aggregated on the pseudoviral surface upon co-incubation with the glycopolymers. Prolonged incubation caused pronounced structural damage to the pseudovirus. Numerous viral fragments (~50 nm) were observed, and intact particles shifted from a rough to a smooth surface, consistent with loss of spike proteins. These changes suggest that strong electrostatic interactions between the glycopolymers and the viral envelope destabilize and compromise pseudovirus integrity.

To further assess antiviral efficacy at the cellular level, we evaluated the ability of the glycopolymers to inhibit pseudovirus infection in 293T/17 cells. Prior to this, cytotoxicity was examined in 293 T/17 cells, where all glycopolymers demonstrated minimal toxicity, maintaining over 90% cell viability even at 100 μM (Figure 3c). After confirming their biocompatibility, we performed concentration-dependent assays to determine the half-maximal inhibitory concentration (IC_50_) values against pseudovirus infection (Figure 3d). Among the tested compounds, **DMP4** exhibited the strongest inhibition (IC_50_ = 104 nM), followed by **MMP** (IC_50_ = 190 nM) and **DMP3** (IC_50_ = 1414 nM). These activities surpassed those of glycopolymers **DMP1** and **DMP2** as well as natural GAG polysaccharides (HP, LWMH, DS), aligning with the binding results observed at the protein level.

**Figure 4:**
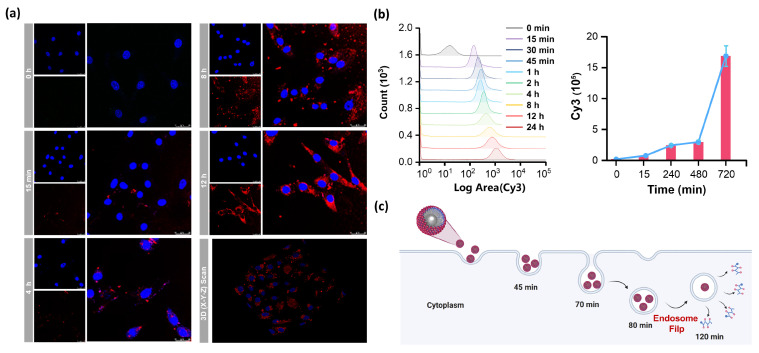
(**a**) Confocal images of Vero cells incubated with **Cy3-DMP4** (20 μg·mL^−1^) for four hours. The scale bar indicates 5 μm. (**b**) Fluorescence intensity of Vero cells incubated with **Cy3-DMP4** obtained by laser scanning confocal microscopy. Flow cytometric analysis of fluorescence intensity in Vero cells incubated with **Cy3-DMP4** (20 μg·mL^−1^) for different times. (**c**) Schematic diagram of entry of DS-mimetic glycopolymer nanoparticles into cells by endocytosis.

Building on the cellular assays and characterization of the self-assembled glycopolymer nanoparticles, we propose that these nanoparticles can enter host cells through endocytosis and subsequently release polysaccharide components via membrane flipping within endosomes (Figure 4c). This process may suppress intracellular replication of SARS-CoV-2 and inhibit key viral and host enzymes, including M^pro^ and HPSE, thereby achieving a coordinated and multi-targeted antiviral effect.

To verify the cellular internalization capability of the glycopolymer nanoparticles, **DMP4** was fluorescently labeled with sulfo-Cy3. Cellular uptake was then examined using confocal fluorescence microscopy (Figure 4a) and flow cytometry at various time points (Figure 4b). The results demonstrated that the **DMP4** nanoparticles were efficiently internalized by Vero cells, with maximal uptake observed between 4 and 8 h post-incubation.

**Figure 5:**
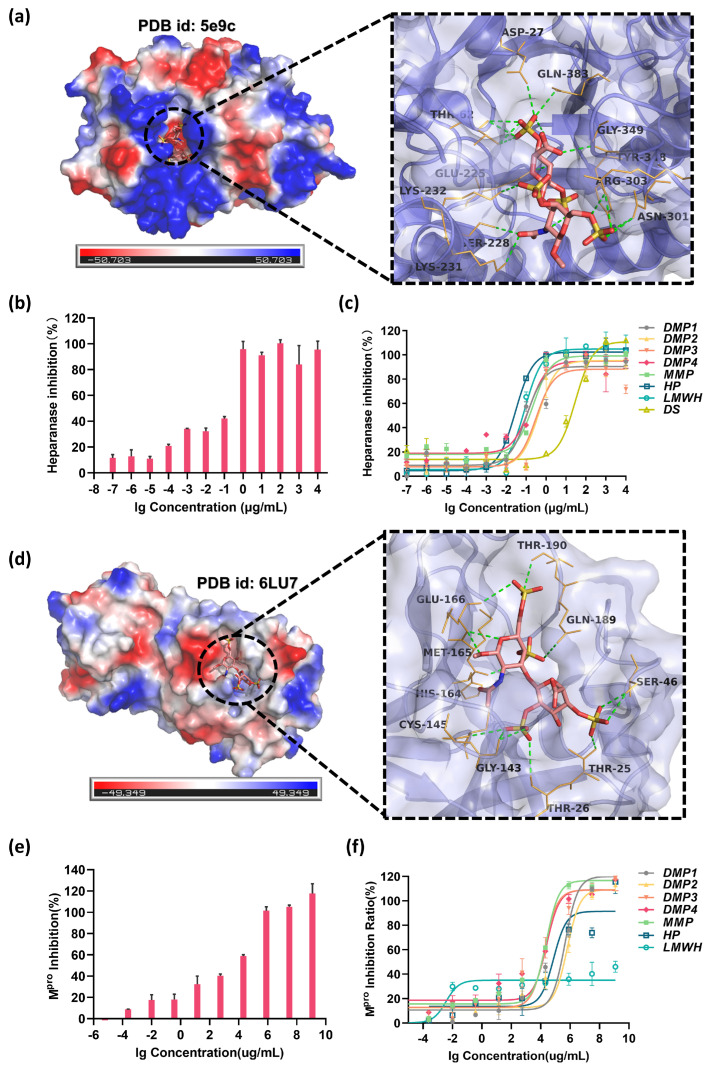
(**a**) Molecular docking of DS-mimetic disaccharides and heparanase protein. (**b**) Inhibition of heparanase by glycopolymer **DMP4** using a TR-FRET assay (mean ± SD, n = 3). (**c**) Inhibition of heparanase by diverse sulfated glycopolymers using a TR-FRET assay (n = 3). (**d**) Molecular docking of DS-mimetic disaccharides and Mpro protein. (**e**) Inhibition assay of Mpro by glycopolymer **DMP4** (n = 3). (**f**) Inhibition assay of Mpro by diverse sulfated glycopolymers (mean ± SD, n = 3).

After confirming cellular internalization of the saccharide polymers as nanoparticles, we assessed their inhibitory activity against HPSE and M^pro^ in concentration-dependent assays. The results were consistent with both molecular docking predictions (Figure 5a,d) and antiviral activity measurements (Table 3). Among the tested polymers, **DMP4** exhibited the strongest inhibition of HPSE, with an IC_50_ comparable to that of natural heparin. In contrast, **DMP3** showed the most potent inhibition on M^pro^, significantly exceeding that of natural GAGs, which is consistent with the docking analysis. These findings suggest that sulfation at the C2 and C4 positions of IdoA in the DS-mimetic disaccharides is critical for effective interaction with key residues in the active site of M^pro^ (Figure 5d).

Both the molecular docking studies and the subsequent biological assays demonstrated that the DS-mimetic disaccharides effectively target the viral spike protein and inhibit the HPSE and M^pro^ enzymes. When formulated into nanoparticles, these disaccharide glycopolymers are efficiently internalized in Vero cells. The glycopolymers exert potent antiviral effects against SARS-CoV-2 by multi-targeted actions at the viral surface, cell membrane, and intracellular environment. These results highlight their promise as a new class of antiviral therapeutics.

## 3. Discussion

### 3.1. Strengths and Key Findings of the Study

In this study, the molecular docking and virtual screening were performed to assess the antiviral potential of sulfated GAG oligosaccharides against SARS-CoV-2 [20,38]. The focus is on key targets in the viral infection process, including the spike protein, as well as M^pro^ and HPSE enzymes involved in post-infection viral proliferation and further spread.

**Figure 6:**
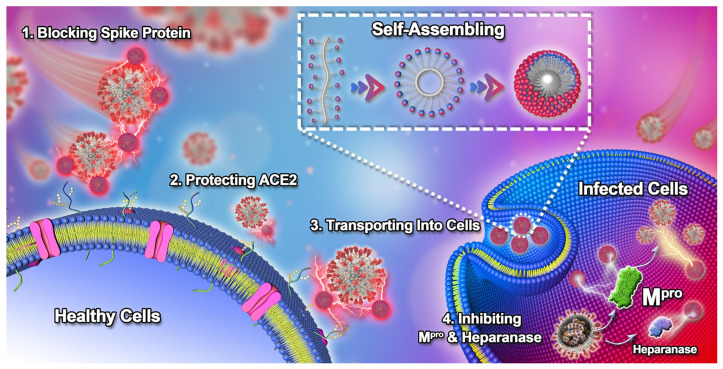
Proposed mechanism of action for DS-mimetic glycopolymers as multi-target antiviral agents against SARS-CoV-2: 1. Sulfated glycopolymers bind to the viral spike protein with high affinity. 2. Low-toxicity glycopolymers bind to and shield ACE2 on healthy cells, preventing the recognition between the viral spike protein and ACE2. 3. The glycopolymers self-assemble into stable nanoparticles, which are internalized by cells via endocytosis. 4. Inside infected cells, the glycopolymers inhibit HPSE and M^pro^ enzymes, suppressing viral replication and further infection.

The screening identifies sulfated DS disaccharide units as promising candidates for antiviral drug development. These DS-mimetic disaccharides are synthesized through chemical methods and grafted onto a polymer backbone under mild and efficient conditions, forming distinct glycopolymers that enhance antiviral activity through multivalent effects [39,50]. SPR assays at the molecular level validate the docking prediction results, while in vitro cellular experiments demonstrate that the glycopolymer nanoparticles self-assembled under physiological conditions are effectively internalized by host cells. Additionally, the inhibitory effects of glycopolymers on M^pro^ and HPSE enzymes are evaluated in infected Vero cells. The surface DS-mimetic glycopolymers inhibit SARS-CoV-2 infection and replication through a multi-targeted mechanism (Figure 6), offering new insights into the development of broad-spectrum antiviral therapeutics based on GAG polysaccharide mimetics.

### 3.2. Study Limitations and Outlooks

This insightful study still possesses several limitations. First, the antiviral activity of the DS-mimetic glycopolymers was evaluated only at the in vitro level. Due to constraints in synthesis scale and the relatively low yield of the chemical sulfation process, the amount of material obtained was insufficient for in vivo antiviral assessment in animal models. Similarly, the current quantities do not allow high-throughput screening against multiple coronavirus variants, limiting our ability to experimentally confirm the broad-spectrum antiviral potential of these glycopolymers. Moreover, the release behavior of the self-assembled glycopolymer nanoparticles requires further validation, particularly regarding the preservation of sulfated oligosaccharide integrity during cellular uptake, as sulfation patterns are critical for intracellular enzyme inhibition. Additionally, due to the structural complexity of GAGs and the diversity of possible sulfation motifs, the molecular docking model in this study did not encompass all structural variants. The DS disaccharide model used in this research does not include the structure with IdoA at the reducing end and GalNAc at the non-reducing end. Previous reports have also suggested that higher-ordered oligosaccharides, such as HS tetrasaccharides, exhibit stronger binding affinity toward M^pro^ [52]. Future studies should therefore extend the screening, synthesis, and biological evaluation to specific oligosaccharides with higher degrees of polymerization.

In our ongoing studies, we focus on the biomimetic synthesis of GAGs and sulfated polysaccharides derived from marine sources with structurally defined oligosaccharides and precise sulfation patterns. These efforts aim to further explore their antiviral activity against SARS-CoV-2, influenza virus, HSV, and other emerging viral pathogens, advancing the development of next-generation glycomimetic antiviral therapeutics.

## 4. Materials and Methods

### 4.1. Materials

Ethyl vinyl ether (EVE, 97%), N-(3-Dimethylaminopropyl)-N′-ethylcarbodiimide hydrochloride (EDC, 98%), and N-hydroxysuccinimide (98%, NHS) were purchased from Aladdin (Shanghai, China) and used without further purification. Nucleic acid dye DAPI and Grubbs-type catalysts (G 3rd) were purchased from Sigma Aldrich (St. Louis, MO, USA) and used directly. Other chemical reagents were purchased from Sinopharm Chemical Reagent Co., Ltd. (Shanghai, China) and used directly. SARS-CoV-2 spike Protein (S1 + S2 ECD, His-tag, cat No. 40589-V08B1) and Human Angiotensin-Converting Enzyme 2 (ACE2) Protein (His-Tag, HPLC-verified, cat No. 10108-H08B) were purchased from Sino Biological (Beijing, China). Minimum essential medium (MEM) was obtained from Gibco (Rockville, MD, USA). Fetal bovine serum (FBS) and trypsin were obtained from Gibco-Invitrogen (Grand Island, NY, USA). Cell proliferation/cytotoxicity assay kit (CCK8) was purchased from Dojindo (Tokyo, Japan). SARS-CoV-2 pseudovirus was purchased from PackGene Biotech (Guangzhou, China). Heparanase assay toolbox was purchased from Cisbio Assay (Codolet, France), and heparinase (human recombinant heparanase 7570-GH) was purchased from R&D systems (Minneapolis, MN, USA). Porcine intestinal heparin (Mw = 15 kDa) was obtained from Linhardt’s laboratory (Troy, NY, USA). Low-molecular-weight heparin (LMWH, Enoxaparine, Mw ≈ 4.5 kDa) was purchased from China Resources Double-crane Pharmaceutical Co., Ltd. Dermatan sulfate (≥90%, Mw ≈ 3.5 kDa) was purchased from Sigma Aldrich (St. Louis, MO, USA).

### 4.2. Synthesis of the Engineered Dermatan Sulfate–Mimetic Glycopolymers

#### 4.2.1. General Procedure for the Preparation of Polymer Containing NHS Ester (**30**, **DP** = **50**)

Compound **29** was synthesized based on our previously published procedure [39]. A solution of **29** (30 mg, 0.094 mmol) in DCM (1 mL) was stirred at −78 °C for 30 min with argon protection. A desirable amount of the Grubbs 3rd catalyst (1.42 mg, 0.0019 mmol, 2%) from the fresh-prepared stock solution in DCM (14.2 mg·mL^−1^, 100 μL) was added to the reaction flask. The mixture was stirred vigorously at −78 °C for 20 min, protected from light, followed by warming up to room temperature. The resultant solution was stirred at room temperature for an additional 1 h, after which TLC indicated full conversion and it was quenched with excess ethyl vinyl ether (EVE). The mixture was stirred for 30 min, the excess Et_2_O (×5 Volume) was added, and a light-brown precipitate was formed. The suspension was centrifuged and then the supernatant was discarded. The precipitate was washed by repeating the process of dissolving in DCM/Et_2_O, centrifuging, and decanting until the supernatant appeared colorless to afford the final polynorbornyl NHS ester **30** (25 mg, DP = 50, 85%) as a gray solid. The polymer was stored at −20 °C and characterized by ^1^H-NMR. ^1^H NMR (500 MHz, DMF-*d*7) *δ* 7.70–7.36 (m, 5H), 6.12–5.54 (m, 125H).

#### 4.2.2. General Procedure for Post-Modification of NHS-Containing Polymer with Sugar Units

Before coupling the NHS-activated polymer with the sugar units, the NHS-containing polymer (1.0 equiv., relative to monomer) was first dissolved in DMF (1.2 mL) to generate solution A. Separately, the terminal amine-functionalized disaccharide (1.2 equiv.) was dissolved in Milli-Q water (200 μL) to form solution B. Solution A was then added to solution B, followed immediately by triethylamine (5 equiv.). The reaction mixture was stirred vigorously at room temperature for 5 h, at which point TLC confirmed complete conversion.

The crude product was purified by dialysis against water for 48 h using a 3.5 kDa MWCO membrane. The resulting solution was lyophilized to afford the glycopolymer as a white powder. The purified materials were stored at −20 °C and characterized by ^1^H NMR.

The degree of sugar substitution (DSS) was determined from ^1^H NMR spectra by integrating the N-acetyl peaks of the grafted disaccharides relative to the backbone reference peaks (olefin protons of the ROMP scaffold).

#### 4.2.3. General Procedure for Fluorescent Labeling of Glycopolymers by Post-Modification

To a solution of glycopolymers in PBS buffer (pH 7.4), EDC/NHS (2.0 equiv.) was added, which was then stirred at room temperature for 30 min. Sulfo-Cyanine3 amine (Sulfo-Cy3-NH_2_) was then added, and the reaction was allowed to proceed for an additional 4 h in the absence of light. TLC analysis confirmed complete fluorescent labeling.

The reaction mixture was purified by dialysis against water for 48 h using a 3.5 kDa MWCO membrane, with protection from light maintained throughout. TLC analysis confirmed the absence of free Cy3 in the dialysate. The dialyzed solution was lyophilized to yield the Cy3-labeled glycopolymer as a pink powder. The final materials were stored at −20 °C in the dark.

### 4.3. SPR Analysis

#### 4.3.1. Immobilization of Protein on a CM5 Sensor Chip

A CM5 chip was first set in a Biacore T200 to immobilize RBD and ACE2 protein for surface plasmon resonance (SPR) analysis. The carboxylate dextran matrix of the sensor chip was washed with PBS-P running buffer (1×, GE healthcare), and then activated with a mixture of 0.4 M EDC and 0.1 M NHS from the kit for 420 s at a flow rate of 10 μL/min. Afterwards, SARS-CoV-2 spike protein (pI 6.52) was immobilized at a concentration of 25 µg/mL in 10 mM sodium acetate (pH 5.0), and ACE2 protein (pI 5.60) was immobilized at a concentration of 25 µg/mL in 10 mM sodium acetate (pH 4.5) onto the chip surface. The remaining binding sites on the chip surface were blocked with 1 M ethanolamine (pH 8.5). The baseline was allowed to stabilize with PBS-P running buffer for at least 2 h before injecting test samples.

#### 4.3.2. Kinetic Binding Affinity Assays

Varying concentrations (0.0305–500 μg/mL) of DS-mimetic glycopolymers were dissolved in the PBS-P running buffer and injected for 60 s at 30 μL/min, followed by a 300 s dissociation phase. Regeneration of the sensor chip after each analysis cycle was performed by injecting 0.5 mM NaOH for 5 s. The response was monitored as a function of time (sensorgram) at 25 °C and subtracted from the response of the reference surface. BIAevaluation software 4.1. was used to evaluate kinetic parameters (Ka: association rate constant; Kd: dissociation rate constant; KD: apparent equilibrium dissociation constant).

### 4.4. Cell Viability Assay

Vero cells (1 × 10^4^ cells per well) were, respectively, cultured into a 96-well microplate overnight with MEM with 10% FBS and 1% Penicillin/Streptomycin. Then, varying concentrations (final working concentration 0.01–100 μM, in MEM) of glycopolymer stock solution were added to specific wells in triplicate and incubated for 24 h at 37 °C under 5% CO_2_. Afterwards, the supernatant was replaced with fresh MEM with 10% CCK8 solution (Dojindo, Japan) and incubated for an additional 2 h, and quenched by adding 10 μL Stop solution. Eventually, OD 450 nm was measured by the microplate reader Tecan Spark 10 M, and the cell viability was calculated, normalized to the controls, and presented as a percentage as follows:

Cell viability (%) = [OD_450_(sample)]/[OD_450_(control)] × 100%.

### 4.5. SARS-CoV-2 Pseudovirus Neutralization Assay

#### 4.5.1. SARS-CoV-2 Pseudovirus Production and Transient Transfection of ACE2

293 T/17 cells were employed to pack the SARS-CoV-2 pseudoviral particles. Cells were cultured with DMEM supplemented (Coring, NY, USA, cat NO. 10-013-CVR) with 10% fetal bovine serum (ExCell, cat No. FND500) and 1% Penicillin Streptomycin solution (Coring, USA, cat No. 25-053-CI). Plasmid transfection was performed in 6-well plates by LipFiter 3.0 Reagent (HANBIO, cat No. HB-LF3-1000). The viral particle-containing supernatant was harvested at 48 h post-transfection and stored at −80 °C.

#### 4.5.2. SARS-CoV-2 Pseudovirus Neutralization Assays

ACE2-pcDNA3.1 plasmid transfection was performed in 6-well plates by LipFiter 3.0 Reagent (HANBIO). The supernatant of 293 T/17 cells was abandoned, and cells were harvested by trypsinization, collected in a centrifuge tube, and centrifuged at 1000 rpm for 5 min, after which the supernatant was discarded, and the cells were resuspended with complete DMEM medium. Afterwards, 293 T/17-ACE2 cells (1.2 × 104 cells per well) was cultured into 96-well microplates (Corning 3917, NY, USA) for an additional 6–8 h at 37 °C under 5% CO2. Supernatant was replaced with complete medium (10% FBS, 1% Penicillin and Streptomycin) with varying concentration samples and incubated for 24 h. Then, the supernatant was replaced with fresh complete medium and incubated for an additional 48 h, and 15 μL Renilla luciferase reagent (Promega, cat lot #E2720) was added to the plate. The plates were placed on an orbital shaker for 2 min, followed by 10 min at room temperature to stabilize the luminescent signal. Eventually, the luminescent signal was measured by the microplate reader SpectraMax Paradigm. Relative light units (RLU) were calculated as follows:

RLU (%) = [RLU(sample)/RLU(DMSO)] × 100%

Cell viability at various compound concentrations was calculated using Excel. The resulting data were then analyzed and plotted using GraphPad Prism 7.0 to generate dose–response curves and calculate the IC50 values.

### 4.6. Confocal Microscopy for the Localization of Cellular Uptake of Glycopolymers

#### 4.6.1. Confocal Microscopy for Internalization of Vero Cell

Vero cells (8 × 10^4^ cells per well) were cultured in 35 mm glass-bottomed Petri dishes (Nest, cat No. 801002-ZX) overnight with MEM (10% FBS, 1% Penicillin and Streptomycin). Afterwards, supernatant was replaced with fresh medium (1% Penicillin and Streptomycin) with 20 μg/mL **Cy3-DMP4** and incubated for 12 h at 37 °C under 5% CO_2_. Then, cells were washed twice with PBS and fixed with 4% paraformaldehyde for 1 h, followed by washing away paraformaldehyde and staining with 200 μL of DAPI for an additional 10 min. Eventually, the supernatant was replaced with PBS and the cellular uptake of glycopolymer nanoparticles was imaged with a confocal microscope (Leica TCS SP8).

#### 4.6.2. Confocal Microscopy for Internalization of Vero Cells at Different Time Points

Vero cells (8 × 10^4^ cells per well) were cultured in 96-well microplate overnight with MEM with 10% FBS and 1% Penicillin/Streptomycin. Then, supernatant was replaced with fresh medium (1% Penicillin and Streptomycin) with 20 μg/mL Cy3-DMP4 and incubated for 15 min, 30 min, 45 min, 1 h, 2 h, 4 h, 8 h, 12 h, and 24 h, respectively. Afterwards, cells were washed twice with PBS and fixed with 4% paraformaldehyde for 1 h, followed by washing away paraformaldehyde and staining with 200 μL of DAPI for an additional 10 min. Eventually, the supernatant was replaced with PBS and the cellular uptake of glycopolymer nanoparticles was imaged with a confocal microscope (Leica TCS SP8). Fluorescence intensities were quantified by ImageJ 1.53c software.

### 4.7. Flow Cytometric Analysis of Cellular Uptake of the Glycopolymers

Vero cells (1 × 10^4^ cells per well) were cultured in 35 mm glass-bottomed Petri dishes (Nest, cat No. 801002-ZX) overnight with MEM (10% FBS, 1% Penicillin and Streptomycin). Then, supernatant was replaced with fresh medium (1% Penicillin and Streptomycin) with 20 μg/mL Cy3-DMP4 and incubated for 15 min, 30 min, 45 min, 1 h, 2 h, 4 h, 8 h, 12 h, and 24 h, respectively. Then, the supernatant of Vero cells was abandoned, and the cells were digested with pancreatin, collected in a centrifuge tube, and centrifuged at 1000 rpm for 5 min, after which the supernatant was discarded, and the cells were washed twice with PBS and prepared in a single-cell suspension. Differential cell counting was performed by flow cytometry (Beckman MoFlo XDP).

### 4.8. TR-FRET Heparanase Inhibition Assay

Heparanase inhibition assays were performed using the heparanase assay toolbox (Cisbio) and human recombinant heparanase 7570-GH-005 (R&D, Lot. DCEM0218041). The homogenous time-resolved fluorescence (HTRF) signal (excitation 337 nm, emission 620 nm and 665 nm) was monitored with a microplate reader Tecan Spark 10 M.

The heparanase inhibition activity assays were performed in a 112-μL reaction (14 μL sample solution, 14 μL heparanase solution, 28 μL biotin-heparan sulphate-Eu cryptate substrate solution, 56 μL Streptavidin-XLent solution) to measure the inhibition constant IC_50_ for glycopolymers. In total, 14 μL varying concentrations (final working concentration 0.1 pg/mL–10 mg/mL) of glycopolymers and 14 μL 5.3 nM heparanase solution in pH 7.5 text buffer (20 mM Tris-HCl, 0.15 M NaCl, and 0.1% CHAPS) were added in a 384-well microplate (Greiner, #781080 384-well) in triplicate. Afterwards, the plate was incubated at 37 °C for 10 min. The enzyme reaction was initiated by adding 28 μL biotin-heparan sulphate-Eu cryptate (Cisbio, #61BHSKAA) substrate solution (0.7 μg/mL in 0.2 M NaCH_3_CO_2_ buffer, pH 5.5) and the plate was incubated at 37 °C for 1 h, followed by the addition of 56 μL Streptavidin-XLent (Cisbio, # 611SAXLA) solution (1.0 μg/mL in 0.1 M NaPO4, 0.8 M KF, 1 mg/mL heparin, 0.1% BSA buffer, pH 7.4) and incubation at 37 °C for 5 min. HTRF signals were monitored by microplate reader at λ_em1_ = 620 nm and λ_em2_ = 665 nm after 60 μs of excitation at λ_ex_ = 337 nm. Specific HTRF signal was expressed as percentage of delta F, and calculated as follows:

Delta F (%) = {[(F_665_/F_620_) sample − (F_665_/F_620_) blank]/(F_665_/F_620_) blank} × 100%.

### 4.9. In Vitro Activity Assays of the SARS-CoV-2 M^pro^ Inhibitors

#### 4.9.1. SARS-CoV-2 M^pro^ Protein Expression In Vitro

Details and SDS-PAGE image can be found in the Supporting Information.

#### 4.9.2. SARS-CoV-2 M^pro^ Inhibition Assay

The M^pro^ inhibitory activity assays were performed following established protocols from previous studies, with minor adjustments to suit our experimental setup [53]. All measurements were conducted within a 100 μL reaction volume (2 μL sample solution, 88 μL M^pro^ solution, 10 μL substrate solution). These setups were used to determine the inhibition constant (IC_50_) of the glycopolymers. In total, 200 nM of M^pro^, 20 μM of substrate, and varying concentrations (final working concentration 0.0274–540 μg/mL) of DS-mimetic glycopolymers were added into different wells in a black, clear-bottomed 96-well plate (Corning, NY, USA). Glycopolymers were dissolved in H_2_O to prepare a stock solution and diluted in the assay buffer (50 mM Tris·HCl, 1 mM EDTA, pH 7.3) to the desired concentrations. In total, 2 μL of diluted glycopolymer solution was added to a solution of Mpro (228 nM) in assay buffer (88 μL), and then the mixture was incubated at room temperature for 30 min. The enzyme reaction was triggered by adding a solution of diluted substrate solution (200 μM) in assay buffer (10 μL). Fluorescence intensity (excitation 340 nm, emission 490 nm) was monitored by microplate reader once every 45 s. The initial reaction velocities (substrate cleaved/second) were calculated by fitting the linear portion of the curves (within the first 10 min of the progress curves) to a straight line using the software Origin Pro (Learning Edition, Version 2025b. OriginLab Corporation, Northampton, MA, USA.). Afterwards, the reaction velocity was converted to enzyme activity, and the inhibition rate was calculated as follows:

Inhibition rate (%) = {1 − [Enzyme activity (compound) − Enzyme activity (blank)]/[Enzyme activity (control) − Enzyme activity (blank)]} × 100%

## 5. Conclusions

In summary, we developed a class of precision-engineered DS-mimetic glycopolymers that demonstrate multi-targeted inhibition of SARS-CoV-2. Based on the molecular docking and structure–activity analysis, regioselective sulfation at C2 and C4 positions of iduronic acid was identified as a key determinant of antiviral potency. These disaccharide modules, when covalently grafted onto a polymer scaffold via post-modification chemistry, self-assembled into uniform nanomicelles with enhanced colloidal stability and controlled surface charge. SPR analyses confirmed that the sulfated glycopolymers, particularly **DMP4**, bound to the viral spike protein with nanomolar affinity while exhibiting weaker and less toxic interactions with ACE2. Electron microscopy and pseudovirus infection assays revealed that these glycopolymers effectively prevented viral attachment through acting as molecular sentinels—adsorbing onto the viral surface and disrupting the integrity of the envelope. Cellular studies further demonstrated efficient endocytic uptake of the nanoparticles and subsequent inhibition of key viral and host enzymes, M^pro^ and HPSE, achieving synergistic antiviral activity across multiple biological targets.

Collectively, this study establishes a versatile platform for the rational design of glycosaminoglycan-mimetic therapeutics. The DS-mimetic glycopolymers combine structural precision, tunable sulfation, and favorable biocompatibility, offering a new strategy for broad-spectrum antiviral development beyond COVID-19. Their multivalent, multi-target mechanism provides a foundation for next-generation antiviral materials capable of addressing emerging viral threats with reduced resistance potential.

## 6. Patents

L.W., G.Y., and C.C. are co-inventors on a patent application describing sulfated glycosaminoglycan-mimetic polymer and the preparation method.

## Figures and Tables

**Table 1:** Composition and physicochemical characteristics of obtained DS-mimetic glycopolymers.

Glycopolymer	Sugar units	M_w_ (NMR) ^*a*^	R_h_ (nm) ^*b*^	R_m_ (nm) ^*c*^	Zeta-potential ^*b*^	DSS ^*d*^
**DMP1**	**DM1** (0S)	33 kDa	348.20	27.98	−18.4	82%
**DMP2**	**DM2** (G4/6S)	36 kDa	327.30	21.09	−21.8	88%
**DMP3**	**DM3** (I2/4S)	31 kDa	246.60	12.52	−24.2	83%
**DMP4**	**DM4** (I2/4S, G4/6S)	33 kDa	269.45	15.83	−23.3	86%
**MMP** ***^e^***	**GalNAc** (3/4/6S)	34 kDa	231.35	21.99	−12.0	84%

*^a^* Molecular weight (Mw) was determined by ^1^H-NMR integration. *^b^* R_h_ and zeta potential were measured with Zetasizer Nano ZS90 instrument. *^c^* R_m_ were measured with TEM and processed with Nano Measurer 1.2. *^d^* DS = Degree of sugar substitution. *^e^* The compound was obtained using an established synthetic protocol [39].

**Table 2:** Binding kinetics of DS-mimetic glycopolymers to SARS-CoV-2 spike and ACE2 protein.

Compound and Protein	*k_a_* (M^−1^s^−1^) *^a^*	*k_d_* (1/s) *^a^*	K*_D_* (M) *^a^*
**DMP1-Spike**	2.25 × 10^4^	1.10 × 10^−3^	4.89 × 10^−7^
**DMP** **2-Spike**	1.09 × 10^4^	3.90 × 10^−3^	3.58 × 10^−7^
**DMP** **3-Spike**	1.61 × 10^4^	4.72 × 10^−3^	2.92 × 10^−7^
**DMP4-Spike**	2.27 × 10^4^	4.00 × 10^−3^	1.77 × 10^−7^
**MMP-Spike**	1.78 × 10^4^	4.75 × 10^−3^	2.67 × 10^−7^
**DMP4-ACE2**	4.41 × 10^3^	4.80 × 10^−3^	1.08 × 10^−6^

*^a^* ka: Association rate constant; kd: dissociation rate constant; KD: apparent equilibrium dissociation constant.

**Table 3:** Inhibition of heparanase and M^pro^ by DS-mimetic glycopolymers.

Entry	Compounds	${IC}_{50}^{a}$ (nM)	${IC}_{50}^{a}$ (μg/mL)	${IC}_{50}^{b}$ (μM)	${IC}_{50}^{b}$ (μg/mL)
**1**	**DMP1**	5.48	0.18	2.01	66.33
**2**	**DMP2**	21.35	0.77	1.31	47.16
**3**	**DMP3**	8.12	0.25	0.47	14.57
**4**	**DMP4**	3.67	0.12	0.89	29.37
**5**	**MMP**	5.39	0.18	0.71	24.14
**6**	**DS**	1249	43.72	-	-
**7**	**LMWH *^c^***	18.13	0.082	>1000	>4500
**8**	**Heparin**	3.74	0.056	8.97	134.55

*^a^* Inhibition of heparanase was assessed by in vitro TR-FRET assay against fluorescently tagged HS. *^b^* Inhibition of M^pro^ was assessed by in vitro fluorescence intensity assay against fluorogenic substrate MCA-AVLQSGFR-Lys (Dnp)-Lys-NH_2_. *^c^* LMWH = low-molecular-weight heparin.

## Data Availability

The data are contained within the article or Supplementary Materials.

## Supplementary Materials

The following supporting information can be downloaded at: https://www.mdpi.com/article/10.3390/md23120486/s1, Table S1. Predicted Binding Affinities of GAG-Mimetic Oligosaccharides with Target Proteins; Figure S1. 2D diagram of receptor-ligand interactions between SARS-CoV-2 RBD and GalNAc-346S (Table S1 Entry-1); Figure S2. 2D diagram of receptor-ligand interactions between SARS-CoV-2 RBD and DM1 (Table S1 Entry-16); Figure S3. 2D diagram of receptor-ligand interactions between SARS-CoV-2 RBD and DM2 (Table S1 Entry-18); Figure S4. 2D diagram of receptor-ligand interactions between SARS-CoV-2 RBD and DM3 (Table S1 Entry-17); Figure S5. 2D diagram of receptor-ligand interactions between SARS-CoV-2 RBD and DM4 (Table S1 Entry-20); Figure S6. 2D diagram of receptor-ligand interactions between SARS-CoV-2 RBD and DS-2S-46S (Table S1 Entry-19); Scheme S1. Retrosynthetic route of DS-mimetic glycopolymers.; Scheme S2. Synthesis of 6. Reagent and conditions; Scheme S3. Synthesis of 9. Reagent and conditions; Scheme S4. Synthesis of 12 and deprotection of 12.; Scheme S5. Synthesis of 15. Reagent and conditions; Scheme S6. Synthesis of 17; Scheme S7. Synthesis of 21, 24, 27, 29; Scheme S8. Synthesis of DS-mimetic glycopolymers; Figure S7. TEM images of pseudovirus; Figure S8. TEM images of co-incubation of the pseudovirus with DMP4 for 5 min at 37 °C; Figure S9. TEM images of co-incubation of the pseudovirus with DMP4 for 2 h at 37 °C; Figure S10. SDS-PAGE analysis of affinity chromatography-purified protein; Figure S11. Confocal images of cells incubated with Cy3-SS (20 μg·mL^−1^) for 12 h [54,55].

## Abbreviations

The following abbreviations are used in this manuscript:

SARS-CoV-2	Severe Acute Respiratory Syndrome-Related Coronavirus 2
COVID-19	Coronavirus Disease 2019
SARS-CoV	Severe Acute Respiratory Syndrome-Related Coronavirus
ACE2	Angiotensin-Converting Enzyme 2
GAG	Glycosaminoglycan
HS	Heparan Sulfate
DS	Dermatan Sulfate
HP	Heparin
RBD	Receptor Binding Domain
HPSE	Heparanase
HSPGs	Heparan Sulfate Proteoglycans
M^pro^	Main Protease
Bn	Benzy
Cbz	Carbobenzyloxy
IdoA	Aldonic Acid
GalNAc	N-Acetylgalactosamine
TR-FRET	Time-Resolved Fluorescence Resonance Energy Transfer
