# Supplementary material for: Precision-Engineered Dermatan Sulfate-Mimetic Glycopolymers for Multi-Targeted SARS-CoV-2 Inhibition
Source: Mar Drugs. 2025 Dec 18;23(12):486. doi: 10.3390/md23120486 (PMC12734876; doi:10.3390/md23120486)
Supplement: Supplementary file 1 [file marinedrugs-23-00486-s001.zip › marinedrugs-4030333-supplementary.pdf]

Electronic Supplementary Information (ESI)

for

Precision-Engineered Dermatan Sulfate-Mimetic Glycopolymers for Multi-Targeted SARS-CoV-2 Inhibition

Lihao Wang [1,†], Lei Gao [1,†], Chendong Yang [1], Mengfei Yin [1], Jiqin Sun [1], Luyao Yang [1], Chanjuan Liu [1], Simon F.R. Hinkley [3], Guangli Yu [1,2,*] and Chao Cai [1,2,*]

[1]Key Laboratory of Marine Drugs of Ministry of Education, Shandong Key Laboratory of Glycoscience and Glycotherapeutics, School of Medicine and Pharmacy, Ocean University of China, Qingdao 266003, People's Republic of China.

[2]Laboratory for Marine Drugs and Bioproducts, Qingdao Marine Science and Technology Center, Qingdao 266003, People's Republic of China.

[3]The Ferrier Research Institute, Victoria University of Wellington, 69 Gracefield Road, Lower Hutt, 5040, New Zealand.

E-mail addresses: caic@ouc.edu.cn (C. Cai), glyu@ouc.edu.cn (G. Yu)

******This article is dedicated to the memory of late Prof. Robert J. Linhardt***

Table of Contents:

1. Materials and Characterization 3

1.1 Materials and reagents 3

1.2 Characterization 3

2. Molecular Modeling and Docking Studies 4

2.1 Construction of GAG-Mimetic Oligosaccharide Models 4

2.2 Docking of Oligosaccharides with the SARS-CoV-2 RBD 4

2.3 Docking of Oligosaccharides with the Heparanese 5

2.4 Docking of Oligosaccharides with the M^{pro} protein 5

2.5 Docking Results of Protein–Oligosaccharide Complexes 6

2.6 Interaction Forces Analysis between Oligosaccharides and SARS-CoV-2 RBD 8

3. Retrosynthetic Analysis of Dermatan Sulfate-mimetic Glycopolymers 11

4. Synthetic Route of DS-mimetic Glycopolymers 12

5. Experimental Procedures and Characterization Data 16

6. NMR Spectra of Compounds and Glycopolymers 37

7. Transmission Electron Microscopy (TEM) Observations 79

7.1 TEM Imaging of the Glycopolymers 80

7.2 TEM Imaging of the SARS-CoV-2 Pseudovirus. 80

8. Dynamic Light Scattering (DLS) and Zeta potential 81

9. SARS-CoV-2 M^{pro} Protein Expression and Purification. 82

10. Confocal Microscopy for Internalization of Different Cells. 83

References 83

1. Materials and Characterization

1.1 Materials and reagents

D-glucose (*D*-Glu, 99%), sodium hydride (NaH, 60% in mineral oil), benzyl bromide (BnBr, 99%), tris(phenylthio)orthoformate (PhS3CLi, 97%), *n*-butyllithium (99%), trifluoroacetic acid (TFA, 99.5%), t-butyldimethylchlorosilane (TBSCl, 98%), trichloroacetonitrile (Cl_3CCN, 99%), 1,8-diazabicyclo[5.4.0]-7-undecene (DBU, 99%), D-galactosamine hydrochloride (D-GalNH$_2$·HCl, 99%), trichloroacetyl chloride (TCA-Cl, 99%), boron trifluoride etherate ($BF_3 \cdot Et_2O$, 98%), thiophenol (PhSH, 98%), *N*-iodosuccinimide (NIS, 99%), trimethylsilyl trifluoromethanesulfonate (TMSOTf, 99.5%), $Pd(OH)_2$ and Pd/C (20 wt% on carbon), benzaldehyde dimethyl acetal (98%), (+/-)-10-camphorsulfonic acid (CSA, 99%), sodium methylate (MeONa, 99%), triethylamine(99.5%), α,α-dimethoxytoluene (99%), 5-aminopentanol (99%) , benzaldehyde (BzH, 99.5%), sodium borohydride ($NaBH_4$, 99%), benzyl chloroformate (Cbz-Cl, 99%), tri-n-butyltin hydride (Bu_3SnH, 98%), 2,2′-Azobis(2-methylpropionitrile) (AIBN, 99%) and Amberlite® IR-120 cation exchange resin (H^+ form) were purchased from Aladdin (Shanghai, China) and used without further purification. Sephadex™ LH-20 was purchased from GE healthcare. Sulfur trioxide triethylamine complex (96%) was purchased from Tokyo Chemical Industry (Tokyo, Japan). Other chemical reagents were purchased from Sinopharm Chemical Reagent Co.Ltd. and used directly. GST Fusion Protein Purification Kit and Dabcyl-KTSAVLQSGFRKME-Edans were purchased from Beyotime (Shanghai, China). Factor Xa protease was purchased from NEB (Beverly, MA, USA).

1.2 Characterization

Nuclear magnetic resonance (NMR) spectra were recorded on an Agilent DD2 500 MHz spectrometer (USA) and calibrated by using residual $CDCl_3$ (^{1}H NMR δ = 7.26 ppm, ^{13}C NMR δ = 77.16 ppm), CD_3OD (^{1}H NMR δ = 3.31 ppm, ^{13}C NMR δ = 49.00 ppm), and D_2O (^{1}H NMR δ = 4.79 ppm) as internal reference. The chemical shifts of all the NMR spectra were reported in delta (δ) units and expressed as parts per million (ppm). The following abbreviations are used to designate multiplicities: s = singlet, d = doublet, t = triplet, q = quartet, m = multiplet, brs = broad singlet. High-resolution electrospray ionization (ESI) mass spectra were measured using LTQ orbitrsp XL (Thermo Fisher, Waltham, MA, USA). Zeta potential and spherical nanomicelle diameters analysis was performed by dynamic light scattering measurement using the zetasizer Malvern Nano ZS 90 nanoparticle analyser (United Kingdom). Transmission electron microscope (TEM) analyse was performed on a JEOL JSM 5410 transmission electron microscope (Tokyo, Japan). Nano Measure image processing software (version 1.2.0) was used to count the particle size and Photoshop CS6 (Adobe, USA) was used for image pseudocoloring. Surface Plasmon Resonance (SPR) experiments were performed on a Biacore

T200 SPR instrument (Cytiva, USA). Absorbance and fluorescence intensity measurements were performed by microplate reader Tecan Spark 10M. (Switzerland). Confocal laser-scanning microscopy was performed by a Leica TCS SP8 confocal scanning microscope (Germany). Flow cytometric analysis was performed by a Beckman MoFlo XDP flow cytometer (US).

2. Molecular Modeling and Docking Studies

2.1 Construction of GAG-Mimetic Oligosaccharide Models

The 3D structures of glycosaminoglycan (GAG)-mimetic oligosaccharides were generated using GLYCAM-Web (http://glycam.org), a modeling platform developed by the Robert J. Woods group at the Complex Carbohydrate Research Center, University of Georgia. [40] The resulting structures were exported in PDB format for use in subsequent docking studies.

2.2 Docking of Oligosaccharides with the SARS-CoV-2 RBD

The 3D structure of the SARS-CoV-2 receptor-binding domain (RBD) (PDB ID: 6MOJ) was obtained from the 2019-nCov Drug Target Information Database (http://ncovtarget.qnlm.ac/web/mg/hm), maintained by Qingdao Marine Science and Technology Center in Qingdao, China. Molecular docking simulations were conducted using Vina-Carb 1.0 (http://glycam.org), a docking program optimized for carbohydrate-protein interactions and developed by the Robert J. Woods group at the University of Georgia.[41]

The docking grid was centered at coordinates (X: -32.81, Y: 25.66, Z: 4.79), with a box size of 23 × 21 × 21 Å^3 to fully encompass the RBD binding site. Receptor and ligand structures were prepared in the required PDBQT format using OpenBabel (version 2.4.1), converted from standard PDB files.

Molecular docking simulations were conducted using Vina-Carb 1.0, with the exhaustiveness parameter set to 20 to ensure adequate sampling of binding poses. The resulting conformations were ranked by binding affinity, and those with the lowest predicted free energies were selected as representative binding modes. Polar interactions between GAG-mimetic oligosaccharides and the RBD were visualized using Edu PyMOL (version 2.4.2). Comprehensive interaction profiling, including full-force analysis and 2D interaction mapping, was conducted using Discovery Studio (version 19.1.0.18287).

2.3 Docking of Oligosaccharides with the Heparanese

The crystal structure of the heparanase enzyme (PDB ID: 5E9C) was obtained from the RCSB Protein Data Bank (http://www.rcsb.org). Prior to docking, water molecules were removed and hydrogen atoms were added to the structure. The docking grid was centered at coordinates (X: -30.2, Y: -7.9, Z: 4.5), with a box size of 32.3 × 49.9 × 69.2 Å^3 to encompass the active site. Receptor and ligand structures were prepared in the required PDBQT format using OpenBabel (version 2.4.1), converted from standard PDB files.

Molecular docking simulations were conducted using Vina-Carb 1.0, with the exhaustiveness parameter set to 20 to ensure adequate sampling of binding poses. The resulting conformations were ranked by binding affinity, and those with the lowest predicted free energies were selected as representative binding modes. Polar interactions between GAG-mimetic oligosaccharides and the heparinase protein were visualized using Edu PyMOL (version 2.4.2). Comprehensive interaction profiling, including full-force analysis and 2D interaction mapping, was conducted using Discovery Studio (version 19.1.0.18287).

2.4 Docking of Oligosaccharides with the M^{pro} protein

The 3D structure of the SARS-CoV-2 M^{pro} protein (PDB ID: 6LU7) was obtained from the 2019-nCov Drug Target Information Database (http://ncovtarget.qnlm.ac/web/mg/hm), maintained by Qingdao Marine Science and Technology Center in Qingdao, China. Molecular docking simulations were conducted using Vina-Carb 1.0 (http://glycam.org), a docking program optimized for carbohydrate-protein interactions and developed by the Robert J. Woods group at the University of Georgia.

The docking grid was centered at coordinates (X: -9.26, Y: 11.52, Z: 68.61), with a box size of 19.23 × 28.94 × 20.25 Å^3 to encompass the active site. Receptor and ligand structures were prepared in the required PDBQT format using OpenBabel (version 2.4.1), converted from standard PDB files.

Molecular docking simulations were conducted using Vina-Carb 1.0, with the exhaustiveness parameter set to 20 to ensure adequate sampling of binding poses. The resulting conformations were ranked by binding affinity, and those with the lowest predicted free energies were selected as representative binding modes. Polar interactions between GAG-mimetic oligosaccharides and the M^{pro} protein were visualized using Edu PyMOL (version 2.4.2). Comprehensive interaction profiling,

including full-force analysis and 2D interaction mapping, was conducted using Discovery Studio (version 19.1.0.18287).

2.5 Docking Results of Protein–Oligosaccharide Complexes

The docking results for GAG-mimetic oligosaccharides with the target proteins are summarized in Table S1. Binding affinities were evaluated based on the calculated docking scores, with lower free energy values indicating stronger predicted interactions.

Table S1. Predicted Binding Affinities of GAG-Mimetic Oligosaccharides with Target Proteins

Entry	Oligosaccharides	Structure	Score		
			RBD	Heparanese	M^{pro}
1	GalNAc-346S	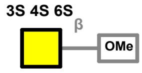	-5.9	-6.8	-6.3
2	HP1-0S	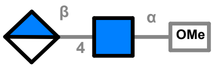	-5.9	-7.2	-6.5
3	HP1-2S-36S	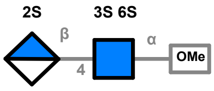	-5.8	-7.2	-7.4
4	HP1-24S-36S	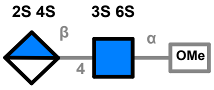	-6.0	-7.3	-7.6
5	HP2-0S	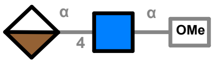	-6.1	-7.7	-6.7
6	HP2-2S-36S	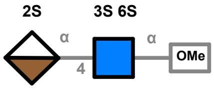	-5.9	-7.7	-7.6
7	HP2-24S-36S	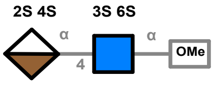	-6.1	-7.9	-7.8
8	CS-0S	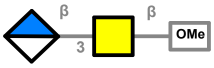	-5.9	-7.3	-7.1
9	CSA	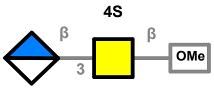	-6.1	-6.8	-7.1
10	CSC	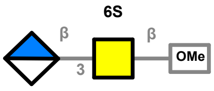	-6.2	-7.5	-7.3
11	CSD	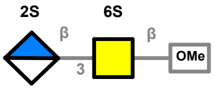	-6.1	-7.1	-7.7
12	CSE	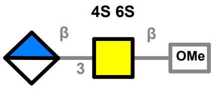	-5.6	-7.2	-7.2

Entry	Oligosaccharides	Structure	Score		
			**RBD**	**Heparanese**	**M^{pro}**
13	CS-2S-46S	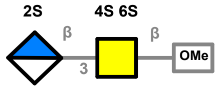	-5.7	-7.3	-6.3
14	CS-24S-46S	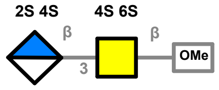	-6.1	-7.5	-6.3
15	CSB (DSA)	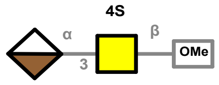	-5.9	-6.9	-6.7
16	DS-0S (DM1)	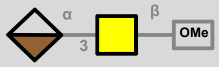	-5.7	-7.6	-7.0
17	DS-24S (DM3)	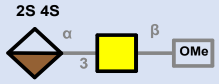	-6.4	-7.3	-7.4
18	DS-46S (DM2) (DSE)	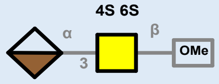	-5.9	-7.2	-6.7
19	DS-2S-46S	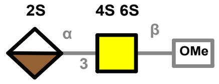	-5.7	-7.6	-6.3
20	DS-24S-46S (DM4)	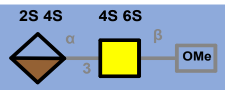	-6.4	-7.4	-7.5
21	DS-3S-46S	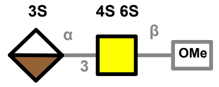	-6.2	-7.6	-7.2
22	DS-234S	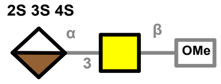	-5.9	-7.0	-6.9
23	DS-234S-46S	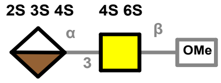	-5.9	-7.0	-6.0
24	GlcNAc-346S	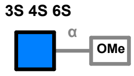	-5.8	-6.8	-7.0

2.6 Interaction Forces Analysis between Oligosaccharides and SARS-CoV-2 RBD

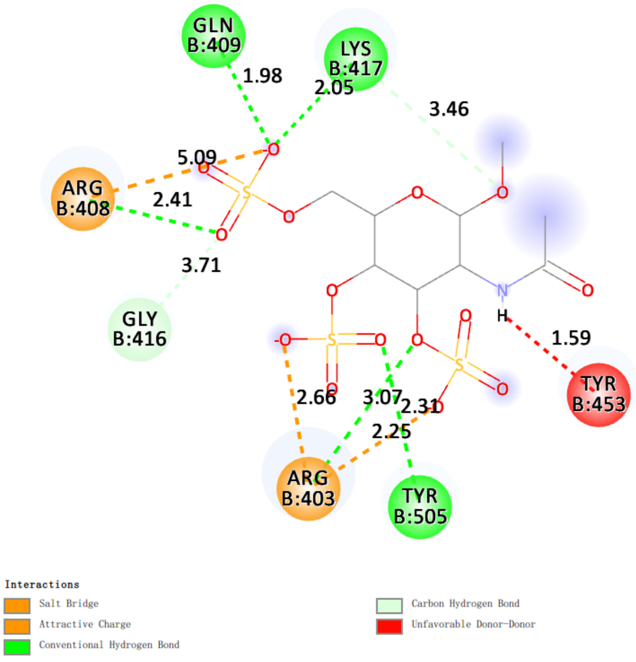

Figure S1. 2D diagram of receptor-ligand interactions between SARS-CoV-2 RBD and GalNAc-346S (**Table S1 Entry-1**)

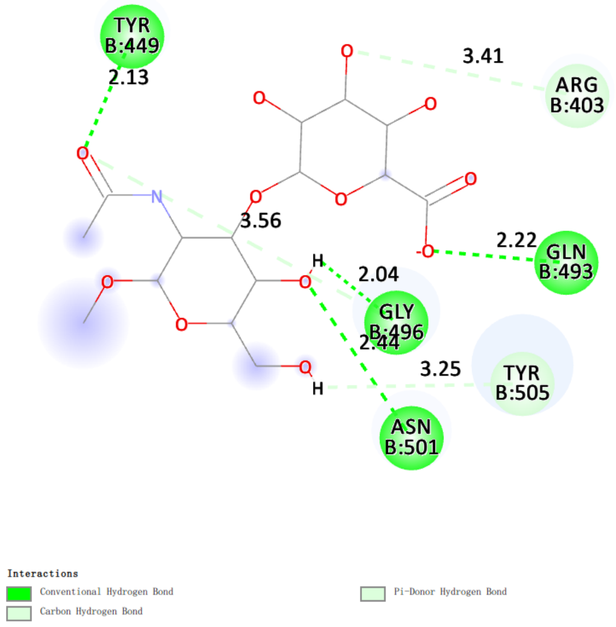

Figure S2. 2D diagram of receptor-ligand interactions between SARS-CoV-2 RBD and **DM1** (**Table S1 Entry-16**)

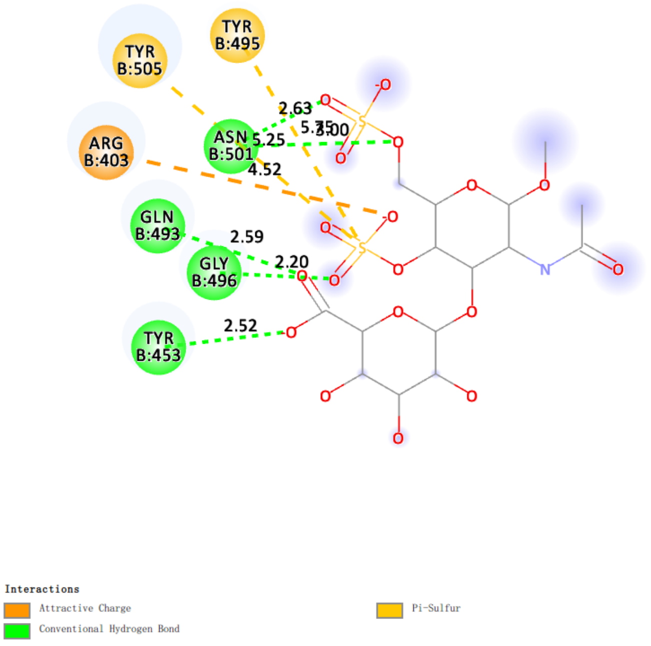

Figure S3. 2D diagram of receptor-ligand interactions between SARS-CoV-2 RBD and **DM2** (**Table S1 Entry-18**)

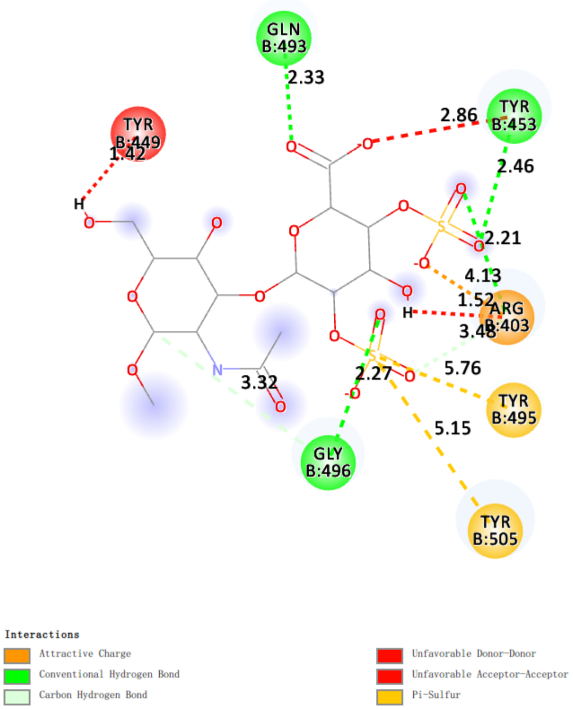

Figure S4. 2D diagram of receptor-ligand interactions between SARS-CoV-2 RBD and **DM3** (**Table S1 Entry-17**)

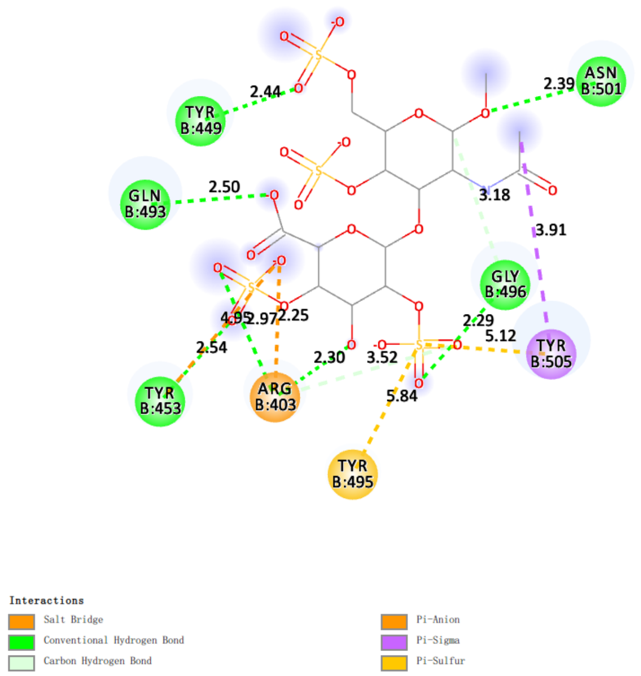

Figure S5. 2D diagram of receptor-ligand interactions between SARS-CoV-2 RBD and **DM4** (**Table S1 Entry-20**)

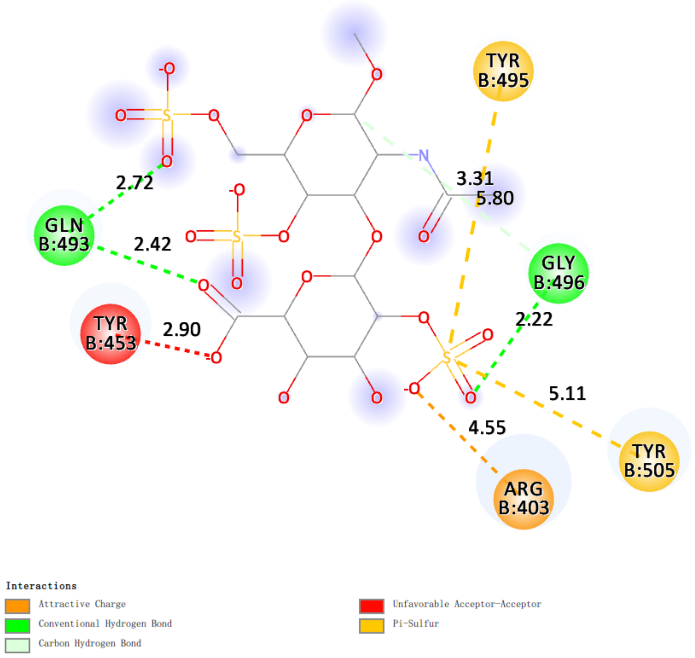

Figure S6. 2D diagram of receptor-ligand interactions between SARS-CoV-2 RBD and **DS-2S-46S** (**Table S1 Entry-19**)

3. Retrosynthetic Analysis of Dermatan Sulfate-mimetic Glycopolymers

By analyzing the results of molecular docking and the structure of natural dermatan sulfate, we performed a retrosynthetic analysis of dermatan sulfate-mimetic disaccharides and glycopolymers with diverse sulfation patterns.

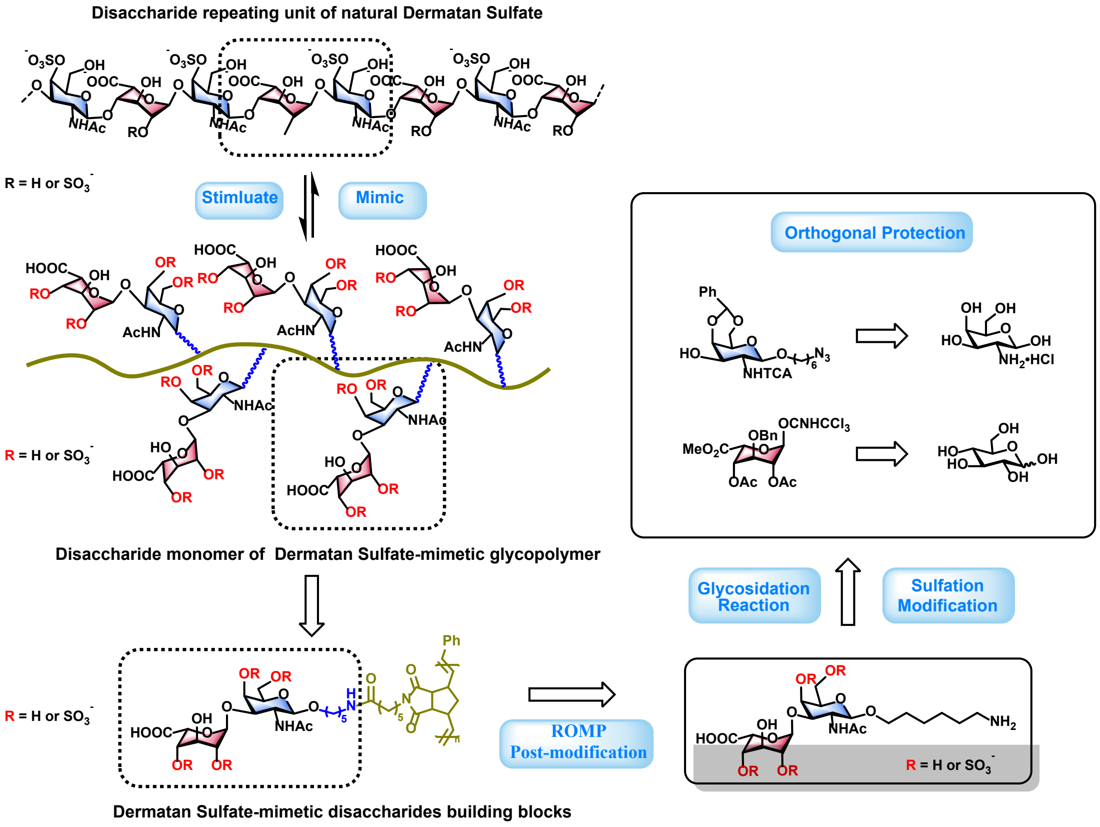

Scheme S1. Retrosynthetic route of DS-mimetic glycopolymers.

4. Synthetic Route of DS-mimetic Glycopolymers

Guided by reported methodologies, the synthesis of compound **6** was strategically designed and implemented.[46]

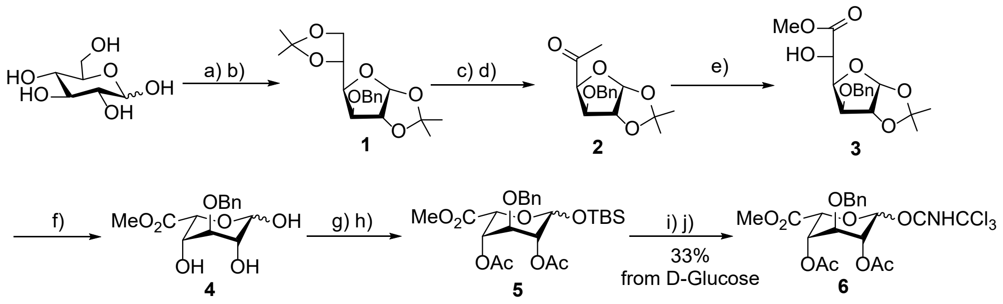

Scheme S2. Synthesis of **6**. Reagent and conditions: a) Na_2SO_4, H_2SO_4, Acetone. b) THF, NaH, TBAI, BnBr. c) 66% AcOH. d) NaIO4, Silica gel, H_2O/DCM. e) 1) PhS_3CLi, -78 ºC, THF. 2) $CuCl_2$, CuO, MeOH/H_2O/DCM. f) 90% TFA. g) Py, TBSCl. h) Py, Ac_2O. i) HF·Py, THF. j) $CNCCl_3$, DBU, DCM.

The synthesis of alcohol receptors **9** was performed using established methods with minor modifications.[39] Specifically, the NHTroc (2,2,2-trichloroethoxycarbonyl) protecting group was replaced with an NHTCA (2,2,2-trichloroacetyl) group to reduce steric hindrance and enhance the yield of β-glycosidic product 8. This substitution also eliminated the need for strong bases and high-temperature conditions, thereby minimizing the risk of sulfate group loss.

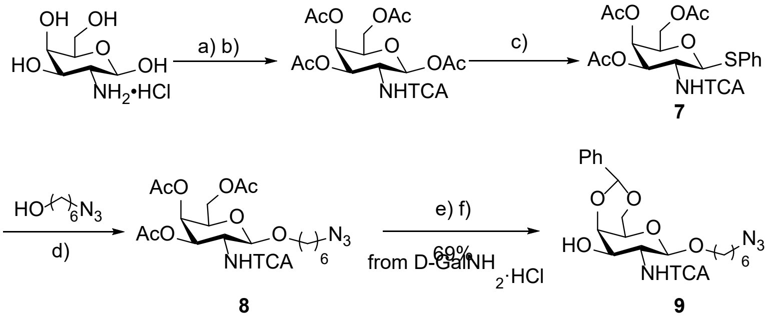

Scheme S3. Synthesis of **9**. Reagent and conditions: a) TCACl, $NaHCO_3$, H_2O, r. t. b) Py, Ac_2O. c) PhSH, $BF_3 \cdot Et_2O$, DCM. d) NIS, DCM, TMSOTf. e) MeOH, MeONa. f) $PhCH(OMe)_2$, CSA, CH_3CN.

During the glycosylation of donor **6** with alcohol acceptor **9** to yield disaccharide 11, a significant amount of the corresponding orthoester intermediate **10** was detected. Formation of such orthoesters is common when acyl-protected glycosyl trichloroacetimidates are employed as donors.[54] Increasing

the amount of TMSOTf from 0.2 to 0.4 equivalents promoted complete conversion of the intermediate to the desired disaccharide **11**.

Subsequent deprotection of **11** presented an additional challenge. In the final reduction step, the trichloroacetyl group could not be transformed into an acetyl group with the coexistence of azido group, despite extensive screening of reduction conditions.

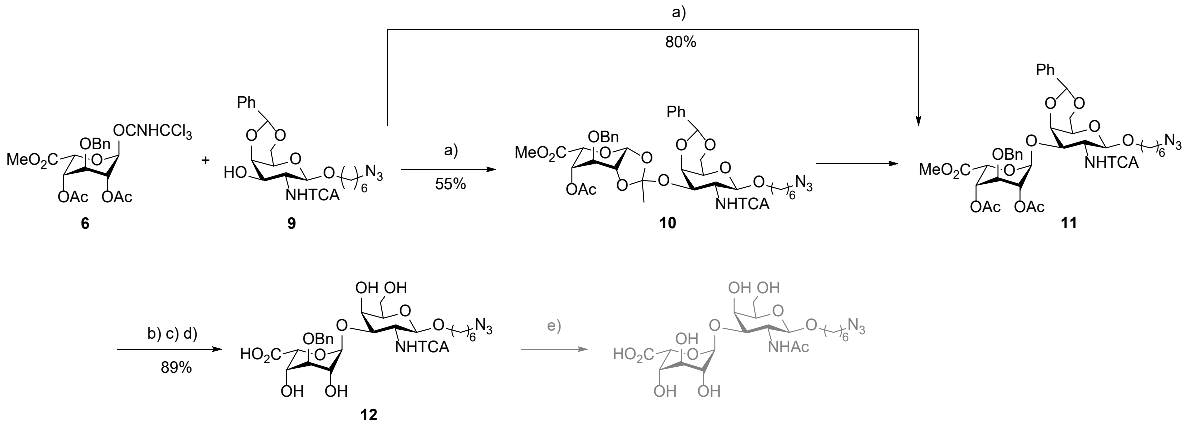

Scheme S4. Synthesis of **12** and deprotection of **12**. Reagent and conditions: a) TMSOTf, DCM, -20 ºC. b) 80% $AcOH/H_2O$, 80 ºC. c) MeOH, MeONa. d) 1M LiOH, 30% H_2O_2. e) AIBN, Bu_3SnH, Toluene and other reduction conditions.

Alcohol **13** was synthesized as a receptor to replace the azide group and was subsequently subjected to glycosylation with donor **7**. The 3-*O*-hydroxy–unprotected derivative **15** was then obtained through a two-step sequential transformation.

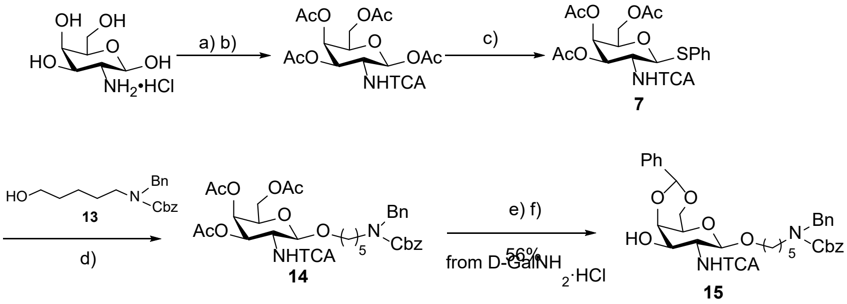

Scheme S5. Synthesis of **15**. Reagent and conditions: a) TCACl, $NaHCO_3$, H_2O, r. t. b) Py, Ac_2O. c) PhSH, $BF_3 \cdot Et_2O$, DCM. d) NIS, DCM, TMSOTf. e) MeOH, MeONa. f) $PhCH(OMe)_2$, CSA, CH_3CN.

Following the synthetic route established for compound **11**, disaccharide module **16** was successfully prepared using donor **6** and acceptor **15**. The trichloroacetyl group was effectively converted into an acetyl group, resulting in the formation of disaccharide module **17**.

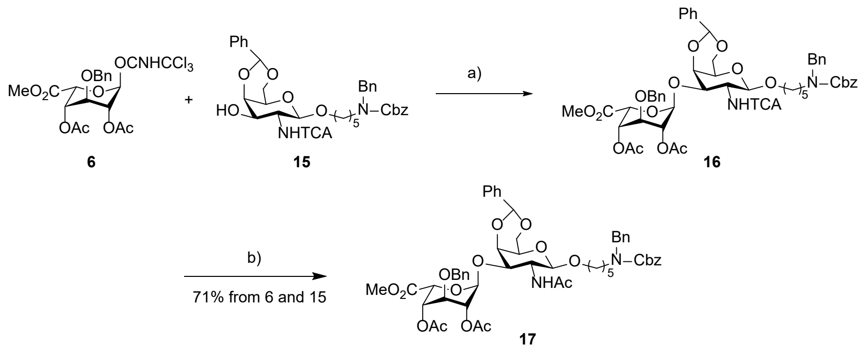

Scheme S6. Synthesis of **17**. Reagent and conditions: a) TMSOTf, DCM, -20 ºC. b) AIBN, Bu_3SnH, Toluene.

Staring from disaccharide module **17**, four different DS-mimetic disaccharides were synthesized via selective deprotection and sulfonation steps.

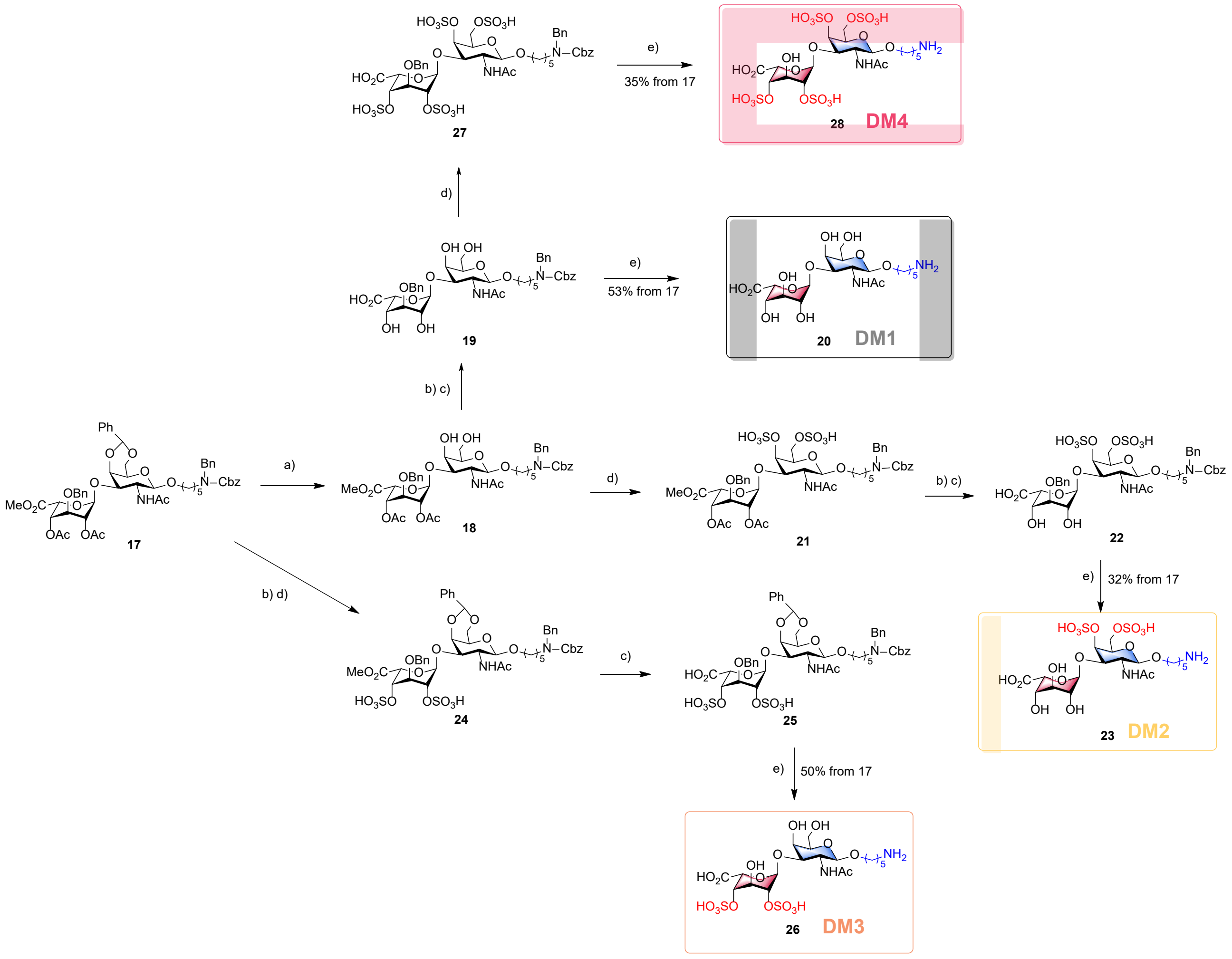

Scheme S7. Synthesis of **21**, **24**, **27**, **29**. Reagent and conditions: a) 50%TFA. b) MeOH, MeONa. c) 1M LiOH, 30% H_2O_2. d) $SO_3{\cdot}Et_3N$, DMF, 55 ºC. e) $Pd(OH)_2/C$, H_2, $MeOH/H_2O$.

DS-mimetic glycopolymers were synthesized via post-modification of NHS-containing polymer backbone with sugar units, following established procedures with minor modifications.[4]

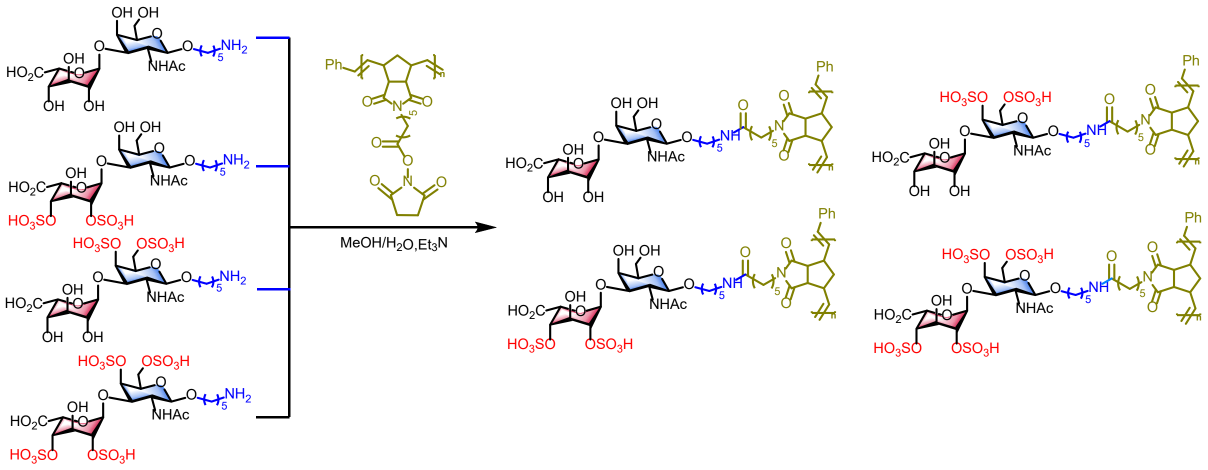

Scheme S8. Synthesis of DS-mimetic glycopolymers.

5. Experimental Procedures and Characterization Data

3-*O*-benzyl-1,2,5,6-bis-*O*-(1-methylethylidene)-*α*-*D*-glucofuranose (1)

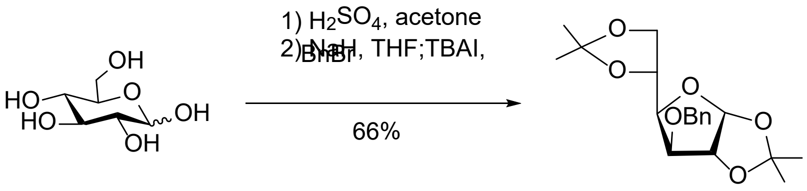

1

To a solution of ***D*-glucose** (240 g, 403.60 mmol, 1.0 eq.) in acetone (1500 mL) at room temperature was added anhydrous Na_2SO_4 (240 g, 3.0 eq.), and concentrated sulfuric acid (8 mL, 149.80 mmol, 0.1 eq.) was slowly added dropwise under stirring. The resultant solution was stirred at room temperature overnight after which TLC indicated full conversion and neutralized with saturated NaOH solution. The solid was filtered off, and the filtrate was concentrated under reduced pressure. The residue was diluted with CH_2Cl_2 (DCM) followed by sequentially washing with saturated aqueous $NaHCO_3$ solution and brine. The combined organic layers were dried over anhydrous Na_2SO_4. The solid was filtered off, and the filtrate was concentrated under reduced pressure to give white solid, which was directly used in the subsequent step without further purification.

To an ice-cooled solution of the crude product (13.22 g, 50.77 mmol, 1.0 eq.) in tetrahydrofuran (THF) (100 mL) was added sodium hydride (NaH) (60% in mineral oil, 4.88 g, 121.8 mmol, 2.4 eq.) in portions. After evolution of hydrogen ceased, tetrabutylammonium iodide (TBAI) (0.13 g, 0.35 mmol, 6.8‰) and benzyl bromide (BnBr) (13.96 mL, 116.7 mmol, 2.2 eq.) were added. The resultant solution was stirred at room temperature overnight after which TLC indicated full conversion. At this point, the reaction was quenched by slowly adding methanol dropwise in an ice bath and the volatile was removed under reduced pressure. The residue was dissolved in DCM and washed with brine. The collected organic layers were dried over anhydrous Na_2SO_4. The solid was filtered off, and the filtrate was concentrated. The residue was purified by silica gel column chromatography (petroleum ether/ethyl acetate 8:1) to afford **1** as yellow syrup (11.75 g, 66%). ^{1}H NMR (500 MHz, $CDCl_3$): δ 7.36 - 7.27 (m, 5H), 5.90 (d, J = 3.7 Hz, 1H), 4.68 (d, J = 11.8 Hz, 1H), 4.64 (d, J = 11.8 Hz, 1H), 4.58 (d, J = 3.5 Hz, 1H), 4.35 (dd, J = 6.2 Hz, 2H), 4.15 (dd, J = 7.7, 3.1 Hz, 1H), 4.11 (dd, J = 8.5, 6.2 Hz, 1H), 4.02 (d, J = 3.6 Hz, 1H), 4.01 - 3.98 (m, 1H), 1.49 (s, 3H), 1.43 (s, 3H), 1.37 (d, J = 5.7 Hz, 3H), 1.31 (s, 3H).

3-*O*-benzyl-1,2-*O*-isopropylidene-*α-D*-xylo-dialdose (2)

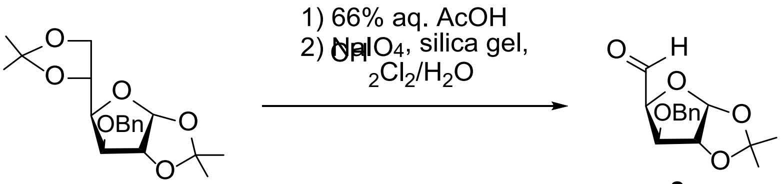

1 2

The purified product **1** (5.22 g, 14.88 mmol, 1.0 eq.) was dissolved in 66% acetic acid (AcOH) (30 mL). The reaction was allowed to stir at room temperature for 14 h after which TLC indicated that most of residue remained unreacted, more 66% AcOH (30 mL) was added in reaction mixture. The resultant solution was stirred at 40 ºC for an additional 2 h after which TLC indicated full conversion. The reaction mixture was evaporated under reduced pressure and the residue was diluted with DCM followed by sequentially washing with saturated aqueous $NaHCO_3$ solution and brine. The combined organic layers were dried over anhydrous Na_2SO_4. The solid was filtered off, and the filtrate was concentrated under reduced pressure to give yellow syrup, which was directly used in the subsequent step without further purification.

To a solution of sodium periodate ($NaIO_4$) (4.14 g, 19.36 mmol) in H_2O (30 mL) was added a solution of silica gel (30 g) in DCM (250 mL) under stirring. The suspension was stirred vigorously for 30 min, followed by addition of a solution of crude product in the previous step (4.62 g, 14.88 mmol) in DCM (24 mL). The resultant solution was stirred at room temperature overnight after which TLC indicated full conversion. The reaction mixture was filtered through a pad of celite and concentrated under reduced pressure to give yellow syrup, which was directly used in the subsequent step without further purification.

Methyl 3-*O*-benzyl-1,2-isopropylidene-*α-L*-idofuranosiduronate (3)

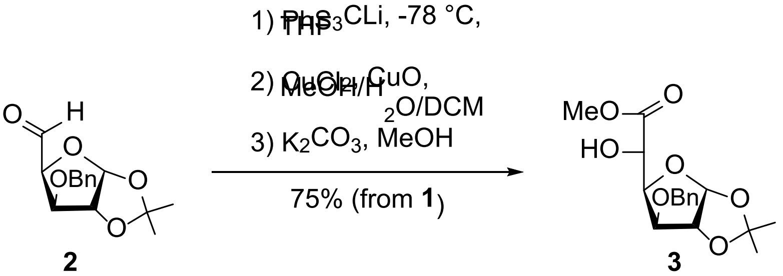

2 3

Tris(phenylthio)orthoformate (PhS_3CLi) (6.10 g, 17.9 mmol) was dissolved in anhydrous THF (50 mL) and was stirred under nitrogen at -50 ºC after which a solution of n-butyllithium (10.24 mL, 122.3 mmol) in anhydrous THF (20 mL) was added dropwise via a fitted dropping funnel over 1 h. A solution of crude **2** (4.6 g, 16.5 mmol) in anhydrous THF (15.75 mL) was added dropwise via dropping funnel to the reaction flask over 1 h. At this point, the reaction was allowed to warm to room temperature over 1 h after which TLC indicated full conversion and quenched with saturated ammonium chloride (NH_4Cl) (16 mL) in an ice bath. The reaction mixture was evaporated under reduced pressure at low temperature and the aqueous phase was extracted with DCM. The combined organic layers were dried over anhydrous Na_2SO_4, filtered, concentrated under reduced pressure. The crude product was directly used in the subsequent step without further purification.

To a solution of $CuCl_2$ (7.62 g, 44.69 mmol) and CuO (2.02 g, 25.25 mmol) in methanol (MeOH) (315 mL) were added H_2O (27.6 mL) under stirring at room temperature, followed by addition of a solution of the crude product from the previous step in DCM (27.6 mL). The resultant suspension was allowed to stir at room temperature for 1 h after which TLC indicated full conversion. The solid was filtered off, and the filtrate was concentrated under reduced pressure. The residue was diluted with DCM followed by sequentially washing with saturated aqueous $NaHCO_3$ solution and brine. The combined organic layers were dried over anhydrous Na_2SO_4, filtered, concentrated under reduced pressure. The residue was purified by silica gel column chromatography (petroleum ether/ethyl acetate 3:1) to afford **3** (4.2 g, 75%) as a yellow syrup. ^{1}H NMR (500 MHz, $CDCl_3$): δ 7.38 - 7.27 (m, 5H), 6.00 (d, J = 3.9 Hz, 1H), 4.72 (d, J = 11.6 Hz, 1H), 4.67 (d, J = 3.3 Hz, 1H), 4.56 - 4.50 (m, 3H), 4.19 (d, J = 2.5 Hz, 1H), 3.74 (s, 3H), 3.29 (d, J = 1.8 Hz, 1H), 1.48 (s, 3H), 1.33 (d, J = 11.6 Hz, 3H).

Methyl (t-butyldimethylsilyl 2,4-di-*O*-acetyl-3-*O*-benzyl-L-idopyranosid)uronate (5)

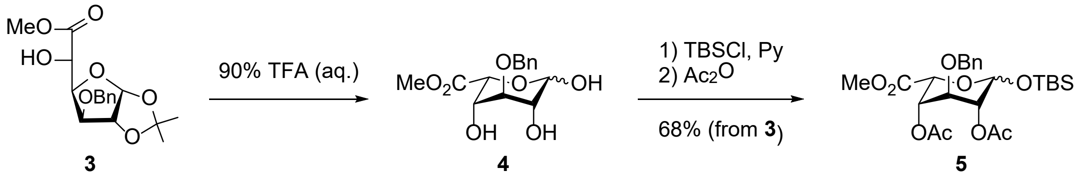

Compound **3** was dissolved in 90% trifluoroacetic acid (TFA) (20 mL) and stirred at -20 ºC for 3 h after which TLC indicated full conversion and the reaction mixture was evaporated under reduced pressure. The resulting white solid **4** (500 mg, 1.68 mmol) was dissolved in pyridine (Py) (16 mL) and

followed by addition of t-butyldimethylchlorosilane (TBSCl) (1.26 g, 8.4 mmol, 5.0 eq.) under stirring at -20 ºC. The resultant solution was stirred at low temperature for 3 h after which TLC indicated full conversion and followed by addition of acetic anhydride (Ac_2O) (8 mL). After stirring at low temperature for 2 h after which TLC indicated full conversion, the mixture was concentrated under reduced pressure. The residue was purified by silica gel column chromatography (petroleum ether/ethyl acetate 8:1) to afford compound **5** as a yellow syrup (1.01 g, 68%). ^{1}H NMR (500 MHz, $CDCl_3$): δ 7.42 - 7.27 (m, 5H), 5.12 (d, J = 6.6 Hz, 2H), 4.94 (s, 1H), 4.76 (d, J = 11.7 Hz, 1H), 4.70 (d, J = 11.8 Hz, 1H), 4.62 (d, J = 1.6 Hz, 1H), 3.93 - 3.83 (m, 1H), 3.76 (s, 3H), 2.06 (s, 3H), 2.03 (s, 3H), 0.88 (s, 9H), 0.17 (s, 3H), 0.11 (s, 3H).13C NMR (126 MHz, CDCl3): δ 170.06, 170.03, 168.00, 137.01, 128.54, 128.14, 127.78, 92.89, 73.63, 72.88, 72.49, 67.69, 67.12, 52.34, 20.86, 20.78, 18.00.

Methyl 2,4-di-*O*-acetyl-3-*O*-benzyl-L-idopyranosiduronate trichloroacetimidate (6)

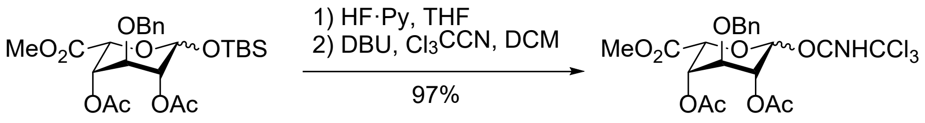

5 **6**

To a solution of purified product **5** (300 mg, 0.60 mmol) in THF (5 mL) was added hydrogen fluoride-pyridine complex (HF·Py) (297.33 mg, 3.0 mmol, 5 eq.) under stirring at -10 ºC. The reaction was allowed to stir at -10 ºC for 6 h after which TLC indicated full conversion and quenched with saturated $NaHCO_3$ solution. The resultant solution was diluted with DCM followed by sequentially washing with saturated aqueous $NaHCO_3$ solution and brine. The combined organic layers were dried over anhydrous Na_2SO_4, filtered, concentrated under reduced pressure. The crude product was dissolved in anhydrous DCM (6 mL) at -10 ºC followed by addition of trichloroacetonitrile (Cl_3CCN) (432 μL, 16.2 mmol, 27 eq.) and DBU (12 μL, 0.3 mmol, 0.5 eq.). The reaction mixture was allowed to stir for 30 min after which TLC indicated full conversion and evaporated e under reduced pressure at a low temperature. The residue was purified by silica gel column chromatography (petroleum ether/ethyl acetate 6:1) to afford compound **6** as a yellow syrup (308 mg, 97%). ^{1}H NMR (500 MHz, $CDCl_3$): δ 8.70 (s, 1H), 7.33 (dt, J = 15.8, 7.4 Hz, 6H), 6.41 (s, 1H), 5.25 (s, 1H), 5.12 (s, 1H), 5.07 (s, 1H), 4.78 (t, J = 12.9 Hz, 1H), 4.71 (d, J = 11.7 Hz, 1H), 3.88 (s, 1H), 3.81 - 3.72 (m, 4H), 2.10 (d, J = 8.8 Hz, 4H), 2.05 (d, J = 11.5 Hz, 4H).13C NMR (126 MHz, CDCl3): δ 169.73, 168.08, 160.01, 136.97,

128.34, 127.92, 127.64, 94.80, 72.42, 71.12, 67.79, 67.39, 64.95, 52.63, 20.79, 20.67. HRMS (ESI) *m/z* calcd for $C_{20}H_{22}Cl_3NNaO_9$ [M+Na]$^+$ 548.0253, found 548.0257.

Phenyl 3,4,6-tri-*O*-acetyl-2-deoxy-2-trichloroacetamido-1-thio-*β*-*D*-galactopyranoside (7)

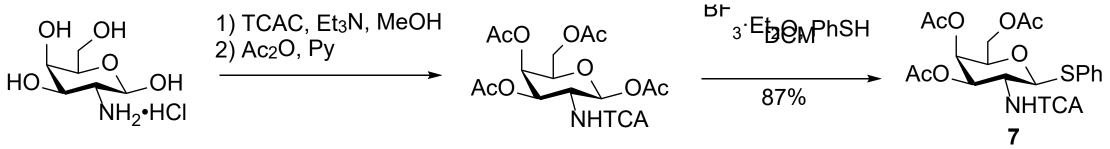

7

To a solution of ***D*-galactosamine hydrochloride** (10 g, 46.37 mmol) in MeOH (100 mL) was added trichloroacetyl chloride (TCAC) (12 mL, 139.13 mmol, 3.0 eq.) in an ice bath followed by addition of triethylamine (Et_3N) to pH 9. The reaction mixture was allowed to stir overnight at room temperature after which TLC indicated full conversion and concentrated by reduced pressure. The residues were co-evaporated with anhydrous toluene twice and dissolved in pyridine (40 mL) followed by addition of acetic anhydride (20 mL). The resultant solution was allowed to stir at room temperature for 7 h and concentrated under reduced pressure. The residue was diluted with DCM followed by sequentially washing with 1 M HCl, saturated aqueous $NaHCO_3$ solution and brine. The combined organic layers were dried over anhydrous Na_2SO_4. The solid was filtered off, and the filtrate was concentrated under reduced pressure to afford yellow syrup, which was directly used in the subsequent step without further purification.

To an ice-cooled solution of the crude product (24.44g, 46.4 mmol) in DCM (100 mL) were added boron trifluoride etherate ($BF_3{\cdot}Et_2O$) (17.52 mL, 6.8 mmol, 3.0 eq.) and thiophenol (PhSH) (11.52 g, 10.46 mmol, 2.0 eq.) and the reaction flask was gradually warmed up to room temperature. The resultant solution was stirred overnight after which TLC indicated full conversion and quenched by adding MeOH in an ice bath. The mixture was concentrated under reduced pressure and diluted with DCM followed by sequentially washing with saturated aqueous $NaHCO_3$ solution and brine. The combine organic layers were dried over anhydrous Na_2SO_4, filtered, concentrated under reduced pressure, and purified via silica gel column chromatography (petroleum ether/ethyl acetate 6:1) to afford **7** as a white solid (21.9 g, 87%). ^{1}H NMR (500 MHz, $CDCl_3$): δ 7.57 - 7.46 (m, 2H), 7.36 - 7.28 (m, 3H), 6.72 (d, J = 8.8 Hz, 1H), 5.40 (d, J = 2.3 Hz, 1H), 5.27 (dt, J = 34.6, 17.3 Hz, 1H), 4.93 (t, J = 15.9 Hz, 1H), 4.24 - 4.06 (m, 3H), 4.02 - 3.89 (m, 1H), 2.13 (s,3H), 2.04 (d, J = 5.2 Hz, 3H),

1.98 (s, 3H).^{13}C NMR (126 MHz, CDCl3): δ 170.40, 170.04, 161.71, 132.92, 132.04, 129.00, 128.44, 86.79, 74.69, 70.54, 66.80, 61.61, 51.37, 20.67, 20.63, 20.51.

6-Azidohexyl 3,4,6-tri-*O*-acetyl-2-deoxy-2-trichloroacetamido-*β-D*-galactopyranoside (8)

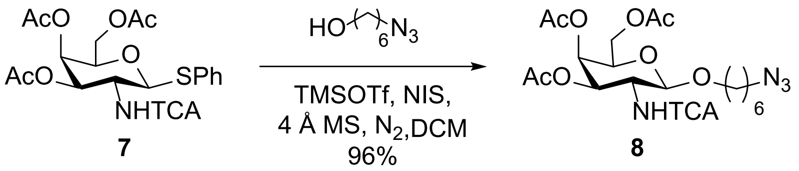

To a solution of thioglycoside donor **7** (700 mg, 1.29 mmol) and alcohol acceptor **6-azidohexanol**[4] (756 mg, 1.94 mmol, 1.5 eq.) in 12 mL anhydrous DCM with N_2 protection was added 4 Å molecule sieves and the suspension was stirred at room temperature for 30 min then cooled to -20 ºC. N-iodosuccinimide (NIS) (600 mg, 2.58 mmol, 2.0 eq.) and trimethylsilyl trifluoromethanesulfonate (TMSOTf) (91 µL, 0.52 mmol, 0.4 eq.) were added to the reaction mixture. The suspension was allowed to stir at low temperature for 30 min after which TLC indicated full conversion and quenched with Et_3N. The resultant suspension was filtered through celite and concentrated under reduced pressure and purified via silica gel column chromatography (petroleum ether/ethyl acetate 4:1) to afford compound **8** (712 mg, 96%) as a white solid. ^{1}H NMR (500 MHz, $CDCl_3$): δ 6.72 (d, J = 8.6 Hz, 1H), 5.38 (t, J = 7.0 Hz, 1H), 5.32 (dd, J = 11.3, 3.3 Hz, 1H), 4.70 (d, J = 8.3 Hz, 1H), 4.16 (qd, J = 11.3, 6.8 Hz, 2H), 4.09 - 4.00 (m, 1H), 3.96 - 3.85 (m, 2H), 3.53 - 3.42 (m, 1H), 3.27 - 3.19 (m, 2H), 2.14 (d, J = 8.9 Hz, 3H), 2.04 (s, 3H), 1.99 (s, 3H), 1.58 (dt, J = 13.7, 7.1 Hz, 4H), 1.37 (tt, J = 19.9, 9.9 Hz, 4H).^{13}C NMR (126 MHz, CDCl3): δ 170.41, 170.35, 170.13, 161.91, 100.69, 92.36, 70.80, 70.11, 69.25, 66.65, 61.29, 53.25, 51.32, 29.29, 28.70, 26.43, 25.47, 20.67, 20.66, 20.51.

6-Azidohexyl 4,6-*O*-benzylidene-2-deoxy-2-trichloroacetamido-*β-D*-galactopyranoside (9)

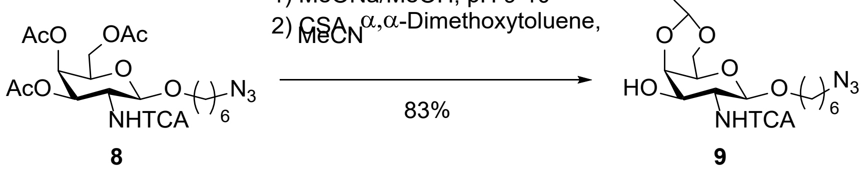

To a solution of compound **8** (720.8 mg, 1.25 mmol) in MeOH (10 mL) was adjusted to pH 10 by adding saturated MeONa in MeOH. The mixture was stirred at room temperature for 1 h after which TLC indicated full conversion and neutralized with Amberlite® IR-120 cation exchange resin (H^+ form), filtered, and concentrated to give the product as white solid, which was directly used in the subsequent step without further purification.

The crude product was dissolved in acetonitrile (MeCN) (10 mL), a catalytic amount of (+/-)-10-camphorsulfonic acid (37.65 mg, 0.16 mmol) and α,α-dimethoxytoluene (488.60 µL, 3.26 mmol, 2.0 eq.) were added. The mixture was stirred at room temperature for 3 h until the TLC indicated full conversion after that concentrated under reduced pressure and purified via silica gel column chromatography (petroleum ether/ethyl acetate 2:1) to give **9** (558 mg, 83%) as a white solid. ^{1}H NMR (500 MHz, $CDCl_3$): δ 7.50 (dd, J = 6.3, 2.7 Hz, 2H), 7.36 (dd, J = 4.7, 1.5 Hz, 3H), 6.93 (d, J = 7.3 Hz, 1H), 5.57 (s, 1H), 4.78 (d, J = 8.3 Hz, 1H), 4.32 (t, J = 11.6 Hz, 1H), 4.26 - 4.13 (m, 2H), 4.07 (t, J = 11.8 Hz, 1H), 3.93 (tt, J = 16.9, 8.4 Hz, 1H), 3.80 (dt, J = 10.3, 7.9 Hz, 1H), 3.52 (s, 1H), 3.47 (dt, J = 9.1, 6.7 Hz, 1H), 3.24 (q, J = 7.3 Hz, 2H), 2.87 (d, J = 9.7 Hz, 1H), 1.66 - 1.49 (m, 4H), 1.43 - 1.29 (m, 4H). ^{13}C NMR (126 MHz, CDCl3): δ 177.38, 162.34, 137.33, 129.29, 128.25, 126.35, 109.99, 101.29, 99.60, 92.53, 74.99, 69.48, 69.17, 69.08, 66.66, 56.72, 51.34, 29.53, 29.32, 28.73, 26.47, 25.55.

Methyl 4-*O*-acetyl-3-*O*-benzyl-*α-L*-idopyranosiduronate 1,2-(6-Azidohexyl 4,6-O-benzylidene-2-deoxy-2-trichloroacetamido-*β-D*-galactopyranoside-3-yl ortho-acetate) (10)

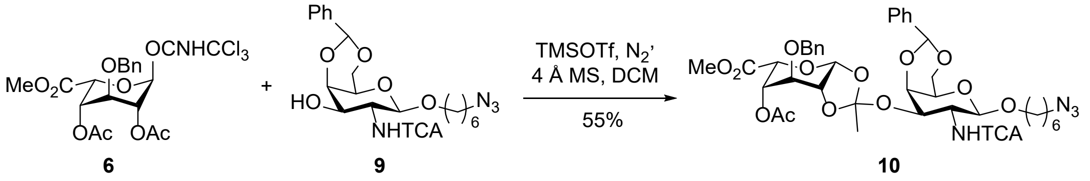

Trichloroacedimidate donor **6** (149 mg, 0.28 mmol) and acceptor **9** (168 mg, 0.31 mmol, 1.1 eq.) were dissolved in anhydrous DCM (10 mL) under N_2 protection. The solution was treated with flame-dried 4 Å molecule sieves and stirred at room temperature for 30 min. At this point, the suspension was cooled to -20 ºC and TMSOTf (10.24 µL, 0.056 mmol, 0.2 eq.) was added. The reaction mixture was stirred for 30 min after TLC indicated full conversion and quenched with Et_3N, filtered through a pad of celite. The filtrate was concentrated under reduced pressure and purified via silica gel column

chromatography (petroleum ether/ethyl acetate 3:1) to afford compound **10** (140 mg, 55%) as a yellow syrup. ^{1}H NMR (500 MHz, $CDCl_3$): δ 8.36 (s, 2H), 7.54 - 7.23 (m, 10H), 7.17 (d, J = 6.8 Hz, 1H), 5.54 (s, 1H), 5.52 (d, J = 1.7 Hz, 1H), 5.15 (d, J = 7.7 Hz, 1H), 4.79 (d, J = 11.7 Hz, 1H), 4.65 (d, J = 11.7 Hz, 1H), 4.53 - 4.45 (m, 2H), 4.33 (d, J = 12.2 Hz, 1H), 4.20 (d, J = 3.1 Hz, 1H), 4.10 - 4.02 (m, 3H), 3.91 (dt, J = 23.5, 8.5 Hz, 1H), 3.75 (s, 3H), 3.60 (dt, J = 10.7, 7.8 Hz, 1H), 3.47 (dd, J = 16.3, 6.9 Hz, 3H), 3.23 (t, J = 6.9 Hz, 2H), 2.03 (s, 3H), 1.73 (s, 3H), 1.69 (s, 3H), 1.54 (dd, J = 17.1, 10.3 Hz, 4H), 1.36 (t, J = 12.2 Hz, 4H). ^{13}C NMR (126 MHz, $CDCl_3$): δ 177.32, 170.19, 167.94, 162.14, 137.73, 136.83, 128.86, 128.56, 128.25, 128.06, 128.04, 126.17, 124.40, 100.71, 98.44, 96.45, 76.02, 75.37, 72.89, 71.31, 69.72, 69.54, 69.10, 68.13, 66.76, 66.69, 54.82, 52.59, 51.35, 29.30, 28.71, 26.48, 25.53, 24.89, 20.82.

6-Azidohexyl *O*-(methyl-2,4-di-*O*-acetyl-3-*O*-benzyl-α-L-idopyranosiduronate)-(1→3)-4,6-*O*-benzylidene-2-deoxy-2-trichloroacetamido-*β*-*D*-galactopyranoside (11)

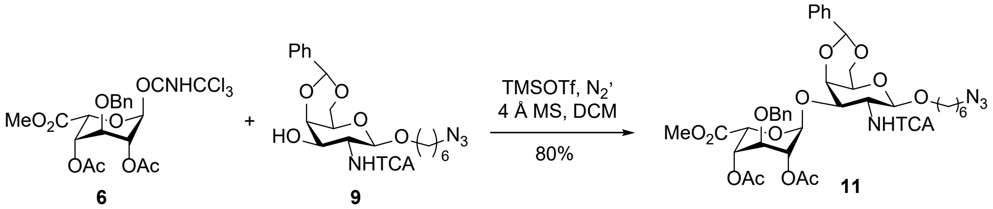

According to the produce of compound **10**, trichloroacedimidate donor **6** (149 mg, 0.28 mmol) and acceptor 9 (168 mg, 0.31 mmol, 1.1 eq.) were reacted with TMSOTf (20.48 μL, 0.112 mmol, 0.4 eq.) for 30 min. After work-up, the residue was purified by silica gel column chromatography (petroleum ether/ethyl acetate 1:1) to afford product **11** (205 mg, 80%) as a yellow syrup. ^{1}H NMR (500 MHz, $CDCl_3$): δ 7.54 - 7.19 (m, 10H), 6.76 (d, J = 6.7 Hz, 1H), 5.45 (d, J = 13.2 Hz, 1H), 5.21 (s, 1H), 5.13 (s, 1H), 5.10 (s, 1H), 5.04 (d, J = 8.3 Hz, 1H), 4.91 (s, 1H), 4.75 (d, J = 12.2 Hz, 1H), 4.68 (d, J = 12.2 Hz, 1H), 4.61 - 4.53 (m, 2H), 4.29 (d, J = 12.2 Hz, 1H), 4.10 (d, J = 11.8 Hz, 1H), 3.92 (dt, J = 9.6, 6.2 Hz, 1H), 3.77 - 3.68 (m, 2H), 3.51 (s, 1H), 3.48 - 3.40 (m, 1H), 3.33 (s, 3H), 3.22 (t, J = 6.9 Hz, 2H), 2.02 - 1.96 (m, 6H), 1.55 (d, J = 6.7 Hz, 4H), 1.35 (s, 4H). ^{13}C NMR (126 MHz, $CDCl_3$): δ 169.70, 169.21, 168.78, 161.79, 137.74, 137.45, 128.85, 128.33, 128.09, 127.74, 126.91, 125.83, 100.53, 100.42, 98.61, 77.09, 74.64, 71.55, 71.27, 69.59, 69.29, 67.86, 66.61, 66.34, 66.08, 54.99, 51.97, 51.34, 29.25, 28.73, 26.45, 25.51, 20.76, 20.66.

6-Azidohexyl *O*-(methyl-3-*O*-benzyl-*α*-*L*-idopyranosiduronate)-(1→3)-2-deoxy-2-trichloroacetamido-*β*-*D*-galactopyranoside (12)

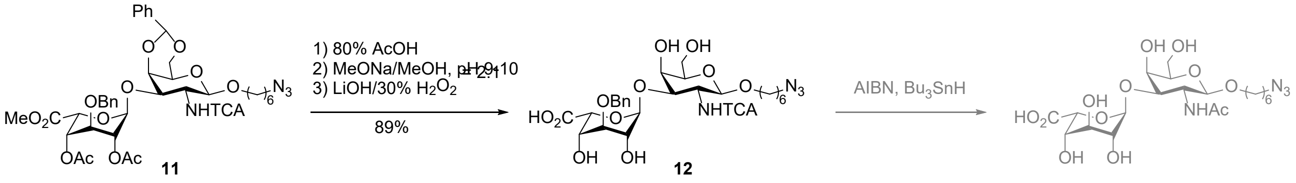

Compound **11** (80 mg, 0.089 mmol) was dissolved in 80% AcOH (2 mL) and stirred at 75 ºC until TLC indicated full conversion. The solvent was evaporated under reduced pressure and the residue was purified via silica gel column chromatography to afford the crude product (40 mg, 56%). The pure product (40 mg, 0.049 mmol) was dissolved in MeOH (2 mL) and adjusted to pH 10 by adding saturated MeONa in MeOH. The mixture was allowed to stir at room temperature until TLC indicated full conversion and neutralized with Amberlite® IR-120 cation exchange resin (H^+ form), filtered, concentrated under reduced pressure to give crude product, which was directly used in the subsequent step without further purification. The crude product was re-dissolved in a mixture of THF and MeOH (THF/MeOH 4:1 *v/v,* 1 mL), a mixed solution of 1 M lithium hydroxide (LiOH) and 30% hydrogen peroxide (H_2O_2) (2:1, *v/v*, 150 μL) was added. The reaction mixture was stirred at room temperature until TLC indicated full conversion and neutralized with Amberlite® IR-120 cation exchange resin (H^+ form). The resin was filtered and concentrated under reduced pressure. The residue was purified by Sephadex LH-20 gel filtration (MeOH) to give the desired product **12** (31.3 mg, 89%).

***N*-benyl-*N*-benzyloxycarbonyl-5-aminopentanol (13)**

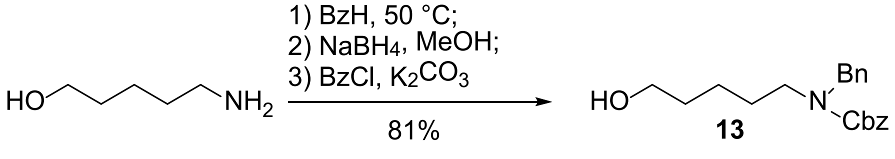

5-Aminopentanol (1 g, 9.69 mmol) was diluted with ethanol (EtOH) and benzaldehyde (BzH) (1.03 g, 9.69 mmol, 1.0 eq.) was added at room temperature. The mixture was stirred for 10 min then concentrated under reduced pressure at 50 ºC and co-evaporated with anhydrous toluene twice. The residue was re-dissolved in MeOH (12 mL) and sodium borohydride ($NaBH_4$) (0.45 g, 11.63 mmol,

1.2 eq.) was slowly added in an ice bath. The resultant solution was allowed to warm up to room temperature and stirred for 2 h and quenched with AcOH (6.46 mL, 6.78 mmol, 0.7 eq.). At this point, an aqueous solution of potassium carbonate (K_2CO_3) (2.25g, 16.28 mmol, 1.68 eq.) was added to the reaction flask. The resultant mixture was stirred for 5 h followed by extraction of ether twice, the combine organic layers were added to aqueous $NaHCO_3$ solution. Benzyl chloroformate (CbzCl, 1.55 mL, 10.67 mmol, 1.1 eq) was slowly added to the resulting biphasic mixture, which was then allowed to warm gradually to room temperature for 14 h until TLC indicated full conversion and diluted with ether (25 mL) followed by sequentially washing with 1 M HCl, saturated aqueous $NaHCO_3$ solution and brine. The combined organic layers were dried over anhydrous Na_2SO_4, filtered, concentrated and purified via silica gel column chromatography (petroleum ether/ethyl acetate 5:1) to give desired compound **13** (2.57 g, 81%) as a colourless transparent oil. ^{1}H NMR (500 MHz, $CDCl_3$): δ 7.28 (dt, J = 37.0, 34.0 Hz, 1H), 5.19 (d, J = 15.2 Hz, 1H), 4.51 (d, J = 9.3 Hz, 1H), 3.53 (t, J = 35.1 Hz, 1H), 3.26 (d, J = 35.8 Hz, 1H), 2.16 (s, 1H), 1.64 - 1.42 (m, 1H), 1.30 (dd, J = 24.6, 13.0 Hz, 1H). Analytical data correspond with those reported. [55]

***N*-(benzyl)-benzyloxycarbonyl-5-aminopentyl-3,4,6-tri-*O*-acetyl-2-deoxy-2-trichloroacetamido-*β-D*-galactopyranoside (14)**

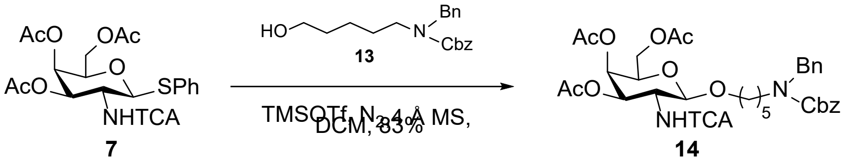

According to the produce for the conversion of compound **8**, thioglycoside donor **7** (700 mg, 1.26 mmol) and the alcohol acceptor **13** (756 mg, 2.30 mmol, 1.5 eq.) were treated with TMSOTf (91 μL, 0.5 mmol, 2 eq.) at -20 ºC for 30 min. After work-up, the residue was purified by silica gel column chromatography (petroleum ether/ethyl acetate 4:1) to afford product **14** (1.2 g, 83%) as a white solid. ^{1}H NMR (500 MHz, $CDCl_3$) δ 7.38 – 7.14 (m, 10H), 5.38 – 5.31 (m, 2H), 5.17 (d, J = 10.8 Hz, 1H), 4.72 (d, J = 7.9 Hz, 1H), 4.51 – 4.47 (m, 2H), 4.13 – 4.05 (m, 2H), 3.92 – 3.78 (m, 2H), 3.54 – 3.13 (m, 4H), 2.14 (s, 3H), 2.04 (s, 3H), 1.98 (s, 3H), 1.63 – 1.24 (m, 6H). ^{13}C NMR (126 MHz, $CDCl_3$) δ 170.46, 170.36, 170.24, 162.02, 156.27, 137.76, 136.66, 128.54, 128.44, 127.93, 127.72, 127.32, 127.21, 100.57, 92.50, 70.70, 69.79, 69.54, 67.18, 66.73, 61.40, 60.41, 53.02, 50.30, 47.13, 46.11,

29.60, 28.80, 27.16, 23.09, 20.68, 20.51. HRMS (ESI) *m/z* calcd for $C_{34}H_{41}Cl_3N_2NaO_{11}$ $[M+Na]^+$ 781.1669, found 781.1681.

N-(benzyl)-benzyloxycarbonyl-5-aminopentyl-4,6-*O*-benzylidene-2-deoxy-2-trichloroacetamido-*β*-*D*-galactopyranoside (15)

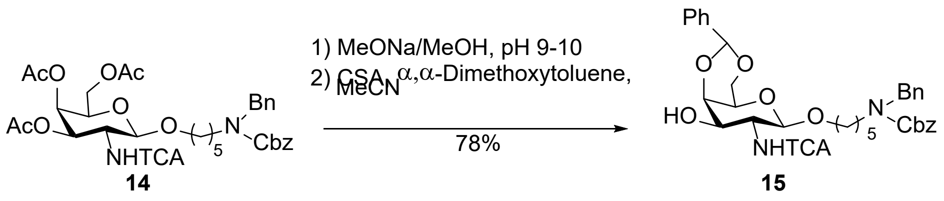

According to the produce for the conversion of compound **9**, the pure product **14** (1.5 g, 1.97 mmol) was treated with MeONa/MeOH, then CSA (45.50 mg, 0.1 eq.) and α,α-Dimethoxytoluene (590.40 μL, 2.0 eq.). After work-up, the residue was purified by silica gel column chromatography (petroleum ether/ethyl acetate 1:1) to afford product **15** (1.15 g, 78%) as a white solid. ^{1}H NMR (500 MHz, $CDCl_3$): *δ* 7.56 - 7.06 (m, 15H), 5.57 (s, 1H), 5.17 (d, *J* = 15.6 Hz, 2H), 4.76 (d, *J* = 8.0 Hz, 1H), 4.47 (s, 2H), 4.32 (d, *J* = 12.4 Hz, 1H), 4.23 - 4.13 (m, 2H), 4.07 (d, *J* = 12.4 Hz, 1H), 3.96 - 3.73 (m, 2H), 3.48 (d, *J* = 12.7 Hz, 1H), 3.26 - 3.12 (m, 2H), 2.93 (d, *J* = 10.0 Hz, 1H), 1.53 (d, *J* = 7.3 Hz, 1H), 1.37 - 1.17 (m, 1H). ^{13}C NMR (126 MHz, $CDCl_3$): *δ* 177.53, 137.82, 137.38, 129.27, 128.53, 128.45, 128.24, 127.91, 127.78, 127.28, 126.36, 101.28, 99.58, 75.05, 69.38, 69.26, 69.09, 67.17, 66.64, 56.63, 50.48, 50.26, 47.13, 46.13, 29.54, 28.95, 27.88, 27.28, 23.20, 18.38. HRMS (ESI) *m/z* calcd for $C_{35}H_{39}Cl_3N_2NaO_8$ $[M+Na]^+$ 743.1665, found 743.1653.

N-(benzyl)-benzyloxycarbonyl-5-aminopentyl-*O*-(Methyl-2,4-di-O-acetyl-3-O-benzyl-*α*-*L*-idopyranosiduronate)-(1→3)-4,6-*O*-benzylidene-2-deoxy-2-trichloroacetamido-*β*-*D*-galactopyranoside (16)

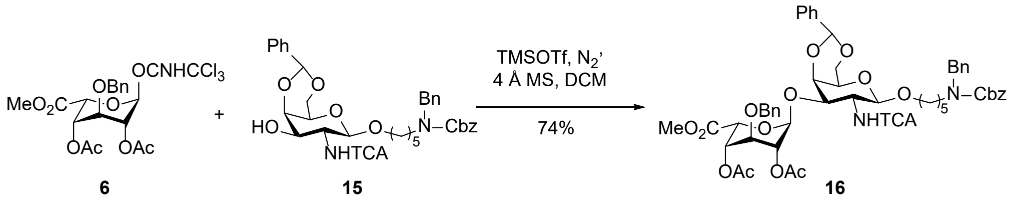

According to the produce of compound **11**, trichloroacedimidate donor **6** (24 mg, 0.046 mmol) and acceptor 15 (33 mg, 0.046 mmol, 1 eq.) were reacted with TMSOTf (3.04 μL, 0.0138 mmol, 0.2 eq.) for 20 min. After work-up, the residue was purified by silica gel column chromatography (petroleum ether/ethyl acetate 3:1) to afford product **16** (36 mg, 74%). ^{1}H NMR (500 MHz, $CDCl_3$): δ 7.59 - 7.08 (m, 20H), 6.95 (d, J = 78.7 Hz, 1H), 5.46 (s, 1H), 5.20 (s, 1H), 5.13 (t, J = 15.4 Hz, 4H), 5.00 (d, J = 8.3 Hz, 1H), 4.91 (s, 1H), 4.74 (d, J = 12.2 Hz, 1H), 4.67 (d, J = 12.1 Hz, 1H), 4.57 (d, J = 9.5 Hz, 2H), 4.46 (d, J = 9.2 Hz, 2H), 4.28 (d, J = 12.3 Hz, 1H), 4.09 (d, J = 12.2 Hz, 1H), 3.85 (d, J = 23.6 Hz, 2H), 3.72 (s, 2H), 3.49 (s, 1H), 3.41 (d, J = 22.8 Hz, 1H), 3.34 (s, 3H), 3.18 (d, J = 36.2 Hz, 2H), 2.01 (s, 3H), 1.99 (d, J = 0.8 Hz, 6H), 1.49 (d, J = 25.9 Hz, 4H), 1.27 (d, J = 5.9 Hz, 2H). ^{13}C NMR (126 MHz, $CDCl_3$) δ 176.29, 169.74, 169.25, 168.82, 161.90, 137.85, 137.63, 137.48, 128.86, 128.51, 128.36, 128.11, 127.82, 127.05, 125.86, 100.46, 98.68, 74.67, 71.58, 71.40, 69.61, 69.29, 67.82, 67.15, 66.59, 66.33, 66.22, 54.87, 51.98, 47.09, 46.09, 44.82, 28.97, 23.25, 22.36, 20.65. HRMS (ESI) *m/z* calcd for $C_{53}H_{59}Cl_3N_2NaO_{16}$ $[M+Na]^+$ 1107.2823, found 1107.2819.

***N*-(benzyl)-benzyloxycarbonyl-5-aminopentyl-*O*-(Methyl-2,4-di-*O*-acetyl-3-*O*-benzyl-*α-L*-idopyranosiduronate)-(1→3)-4,6-*O*-benzylidene-2-deoxy-2-acetamido-*β-D*-galactopyranoside (17)**

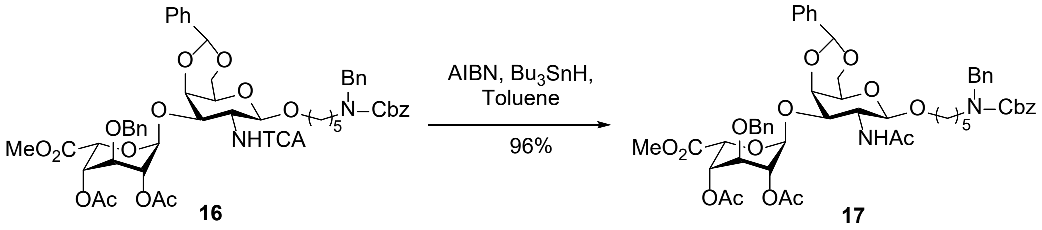

To a solution of compound **16** (20 mg, 0.018 mmol) in toluene (1 mL) was added tri-n-butyltin hydride (Bu_3SnH) (86 μL, 0.108 mmol, 6.0 eq.) under N_2 protection followed by addition a solution of recrystallized 2,2′-Azobis(2-methylpropionitrile) (AIBN) (16 mg, 0.09 mmol, 5.0 eq.) in toluene. The resultant solution was stirred under N_2 protection at room temperature for 30 min and heated to 90 ºC under reflux condition for an additional 2 h. At this point, TLC showed the reaction was completed. The mixture was concentrated under reduced pressure and the residue was purified via silica gel column chromatography (petroleum ether/ethyl acetate 1:2) to afford product **17** (17.3 mg, 96 %) as a yellow oil. ^{1}H NMR (500 MHz, $CDCl_3$): δ 7.50 - 7.11 (m, 20H), 5.81 - 5.48 (m, 1H), 5.43 (s, 1H),

5.25 - 5.01 (m, 5H), 4.88 (s, 1H), 4.71 (s, 3H), 4.47 (s, 3H), 4.27 (d, J = 11.7 Hz, 1H), 4.11 (s, 1H), 4.06 (d, J = 12.0 Hz, 1H), 3.85 (d, J = 30.7 Hz, 1H), 3.77 (s, 1H), 3.48 (s, 1H), 3.41 (s, 3H), 3.20 (d, J = 25.9 Hz, 2H), 2.02 (s, 3H), 2.01 (s, 3H), 1.86 (d, J = 20.3 Hz, 3H), 1.52 (d, J = 22.7 Hz, 4H), 1.29 (s, 2H). ^{13}C NMR (126 MHz, $CDCl_3$): δ 169.76, 169.43, 168.86, 137.87, 137.68, 128.67, 128.50, 128.35, 128.00, 127.83, 127.70, 127.09, 125.88, 100.58, 100.38, 99.03, 75.12, 72.27, 71.66, 69.41, 68.04, 67.13, 66.95, 66.52, 66.23, 60.38, 54.58, 52.03, 50.19, 47.18, 29.68, 28.93, 27.83, 27.37, 23.46, 23.14, 21.03, 20.82, 20.70, 14.18, 13.59. HRMS (ESI) *m/z* calcd for $C_{53}H_{62}N_2NaO_{16}$ $[M+Na]^+$ 1005.3992, found 1005.3988.

***N*-(benzyl)-benzyloxycarbonyl-5-aminopentyl-*O*-(Methyl-2,4-di-*O*-acetyl-3-*O*-benzyl-α-L-idopyranosiduronate)-(1→3)-2-deoxy-2-acetamido-*β*-*D*-galactopyranoside (18)**

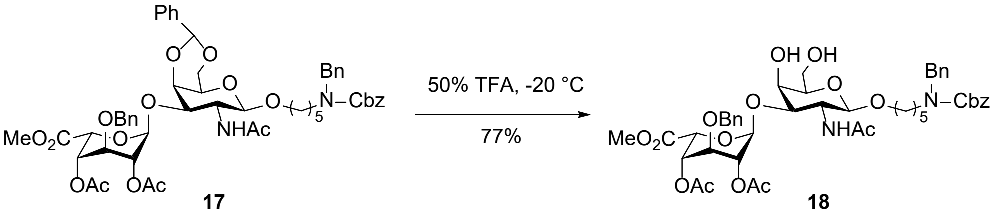

17 **18**

The pure product **17** was dissolved in 50% TFA at -10 ºC and stirred for 4 h after which TLC indicated full conversion. The reaction mixture was evaporated under reduced pressure and the residue was purified by silica gel column chromatography to afford product **18** (10 mg, 77%). ^1H NMR (500 MHz, $CDCl_3$): δ 7.46 - 7.12 (m, 15H), 5.21 - 5.12 (m, 3H), 5.10 (s, 1H), 5.06 (d, J = 2.0 Hz, 1H), 4.89 (s, 1H), 4.76 (d, J = 11.5 Hz, 1H), 4.64 (d, J = 11.3 Hz, 1H), 4.55 (d, J = 8.3 Hz, 1H), 4.50 (s, 1H), 3.96 (s, 2H), 3.90 - 3.69 (m, 6H), 3.52 – 3.38 (m, 2H), 3.36 (s, 1H), 3.29 - 3.15 (m, 2H), 2.05 (d, J = 7.0 Hz, 6H), 1.96 (d, J = 15.8 Hz, 3H), 1.54 (d, J = 6.6 Hz, 4H), 1.28 (d, J = 14.8 Hz, 2H). ^{13}C NMR (126 MHz, $CDCl_3$): δ 173.99, 173.63, 173.15, 141.41, 140.72, 132.40, 132.01, 131.94, 131.62, 131.45, 131.20, 131.03, 104.50, 103.87, 83.94, 83.75, 78.32, 76.54, 76.45, 73.02, 72.13, 71.19, 70.74, 65.34, 56.33, 56.18, 54.15, 51.10, 50.14, 33.50, 32.88, 31.08, 26.91, 26.66, 24.46, 24.35. HRMS (ESI) *m/z* calcd for $C_{46}H_{58}N_2NaO_{16}$ $[M+Na]^+$ 917.3679, found 917.3671.

N-(benzyl)-benzyloxycarbonyl-5-aminopentyl-*O*-(Methyl-3-*O*-benzyl-*α*-*L*-idopyranosiduronate)-(1→3)-2-deoxy-2-acetamido-*β*-*D*-galactopyranoside (19)

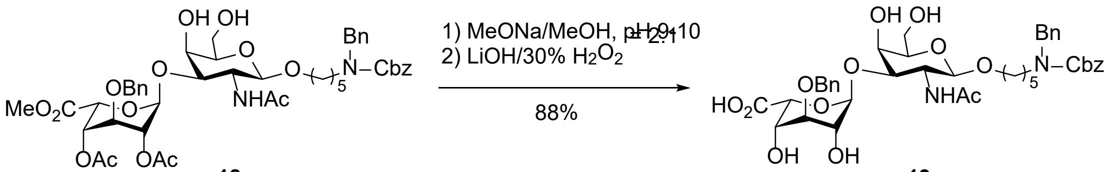

The pure product **18** (17 mg, 0.019 mmol) was dissolved in MeOH (1 mL) and adjusted to pH 10 by adding saturated MeONa in MeOH. The mixture was allowed to stir at room temperature for 3 h after which TLC indicated full conversion and neutralized with Amberlite® IR-120 cation exchange resin (H^+ form), filtered, concentrated under reduced pressure to give crude product, which was directly used in the subsequent step without further purification. The crude product was re-dissolved in a mixture of THF and MeOH (THF/MeOH 4:1 *v/v*, 1 mL), a mixed solution of 1 M LiOH and 30% H_2O_2 (2:1, *v/v,* 150 μL) was added at -10 ºC. The mixture was stirred at low temperature until TLC indicated full conversion and neutralized with Amberlite® IR-120 cation exchange resin (H^+ form). The resin was filtered and the filtrate was concentrated under reduced pressure. The residue was purified via Sephadex LH-20 gel filtration (MeOH) to give the desired product **19** (13 mg, 88%). ^1H NMR (500 MHz, CD_3OD): δ 7.48 - 7.22 (m, 15H), 5.16 (d, J = 18.3 Hz, 2H), 4.77 (d, J = 11.3 Hz, 1H), 4.70 (d, J = 11.3 Hz, 1H), 4.60 (s, 1H), 4.51 (s, 2H), 4.36 (s, 1H), 4.13 (s, 1H), 3.99 (d, J = 2.8 Hz, 2H), 3.81 (d, J = 29.1 Hz, 1H), 3.77 - 3.69 (m, 3H), 3.62 (s, 1H), 3.59 (t, J = 5.6 Hz, 1H), 3.51 (t, J = 5.8 Hz, 1H), 3.25 (s, 1H), 1.93 (d, J = 14.9 Hz, 3H), 1.51 (d, J = 17.6 Hz, 4H), 1.26 (s, 4H). ^{13}C NMR (126 MHz, CD_3OD) δ 138.23, 128.17, 127.89, 127.52, 127.26, 126.90, 103.12, 101.53, 79.91, 78.91, 75.12, 72.53, 70.09, 70.04, 69.08, 68.74, 68.13, 67.03, 66.94, 61.15, 51.43, 46.47, 46.13, 28.84, 22.85, 21.72. HRMS (ESI) *m/z* calcd for $C_{37}H_{43}N_2O_{14}$ $[M-H]^-$ 739.2719, found 739.2705.

5-Aminopentyl-*O*-*α*-*L*-idopyranosiduronate-(1→3)-2-deoxy-2-acetamido-*β*-*D*-galactopyranoside (20)

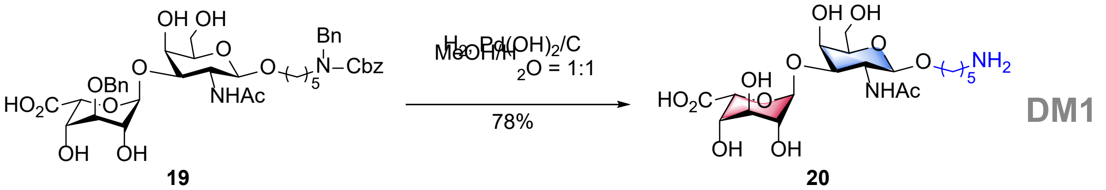

Compound **19** (15 mg, 0.019 mmol) was dissolved in a mixture of MeOH/H_2O (1:1, *v/v,* 1 mL), the solution was purged with H_2 and a catalytic amount of 20% $Pd(OH)_2/C$ was added. The suspension was purged with H_2 for 5 min and stirred under an H_2 atmosphere for 1 h, filtered (celite) and concentrated to give **20** (8.4 mg, 78%) as a white solid. 1H NMR (500 MHz, D_2O) δ 4.80 (d, J = 4.3 Hz, 1H), 4.46 (d, J = 8.5 Hz, 1H), 4.40 (d, J = 3.5 Hz, 1H), 4.01 – 3.91 (m, 2H), 3.88 – 3.75 (m, 3H), 3.72 – 3.53 (m, 7H), 3.45 – 3.40 (m, 1H), 2.93 – 2.87 (m, 1H), 1.97 (s, 3H), 1.58 (ddd, J = 30.0, 14.8, 7.3 Hz, 4H), 1.31 (ddd, J = 52.1, 21.4, 13.8 Hz, 4H). HRMS (ESI) *m/z* calcd for $C_{19}H_{33}N_2O_{12}$ [M-H]$^-$ 481.2038, found 481.2015.

N-(benzyl)-benzyloxycarbonyl-5-aminopentyl-*O*-(Methyl-2,4-di-*O*-acetyl-3-*O*-benzyl-*α-L*-idopyranosiduronate)-(1→3)-4,6-di-*O*-sulfo-2-deoxy-2-acetamido-*β-D*-galactopyranoside (21)

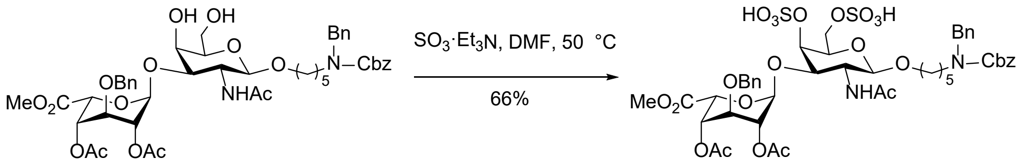

To a solution of compound **18** (23 mg, 0.026 mmol) in DMF (1 mL) was added sulfur trioxide-triethylamine complex ($SO_3 \cdot Et_3N$) (188.5 mg, 1.04 mmol, 40 eq.). The mixture was stirred at 50 ºC for 10 h after which TLC indicated full conversion and purified via Sephadex LH-20 gel filtration (MeOH) to give desired compound **21** (18 mg, 66%) as a syrup. ^{1}H NMR (500 MHz, CD_3OD) δ 7.41 – 7.15 (m, 15H), 5.59 (d, J = 2.8 Hz, 1H), 5.28 (t, J = 3.3 Hz, 1H), 5.15 (d, J = 19.6 Hz, 2H), 4.98 (d, J = 6.3 Hz, 2H), 4.72 (d, J = 2.1 Hz, 1H), 4.67 (d, J = 12.3 Hz, 1H), 4.62 (d, J = 12.2 Hz, 1H), 4.51 (s, 2H), 4.39 (dd, J = 11.8, 2.8 Hz, 2H), 4.35 – 4.26 (m, 1H), 4.03 – 3.87 (m, 3H), 3.78 (d, J = 11.6 Hz, 1H), 3.64 (t, J = 4.6 Hz, 1H), 3.48 – 3.35 (m, 1H), 3.24 (s, 2H), 2.00 (s, 3H), 1.97 (s, 3H), 1.94 (dd, J = 11.4, 6.4 Hz, 3H), 1.57 – 1.41 (m, 5H), 1.27 – 1.19 (m, 3H). ^{13}C NMR (126 MHz, CD_3OD) δ 171.95, 169.80, 169.48, 169.32, 137.73, 136.69, 128.19, 127.96, 127.79, 127.58, 127.30, 126.94, 101.20, 75.24, 73.10, 71.67, 69.81, 68.96, 68.57, 68.31, 68.04, 66.89, 51.93, 51.36, 50.07, 49.85, 48.44,

46.17, 28.88, 27.54, 27.05, 22.85, 21.85, 19.47, 19.28. HRMS (ESI) *m/z* calcd for $C_{46}H_{57}N_2O_{22}S_2$ [M-H]$^-$ 1053.2849, found 1053.2815.

N-(benzyl)-benzyloxycarbonyl-5-aminopentyl-O-(3-O-benzyl-α-L-idopyranosiduronate)-(1→3)-4,6-di-O-sulfo-2-deoxy-2-acetamido-β-D-galactopyranoside (22)

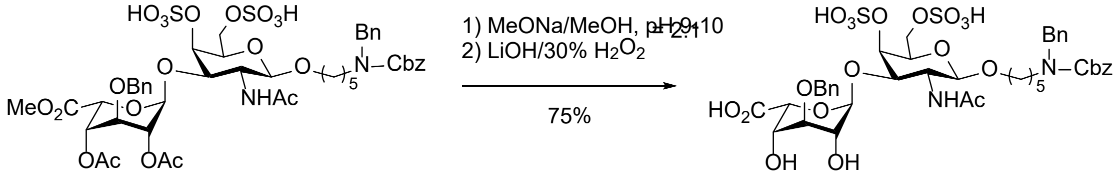

According to the produce for the conversion of compound **18** to compound **19**, compound **21**(12 mg, 0.011 mmol) was sequentially treated with MeoNa and 1 M LiOH/30 %H_2O_2 (2:1 *v/v*, 150 μL). Amberlite® IR-120 cation exchange resin (H^+ form) was added to neutralize the reaction mixture, filtered. The solvent was evaporated under reduced pressure and the residue was purified by Sephadex LH-20 gel filtration (MeOH) to give desired compound **22** (8 mg, 75%) as a syrup. ^{1}H NMR (500 MHz, CD_3OD) δ 7.53 – 7.07 (m, 15H), 5.16 (d, J = 19.2 Hz, 2H), 4.97 (s, 1H), 4.78 (s, 2H), 4.71 (d, J = 2.5 Hz, 1H), 4.52 (s, 2H), 4.47 – 4.36 (m, 2H), 4.33 – 4.24 (m, 1H), 4.05 (dd, J = 10.4, 6.1 Hz, 2H), 3.96 (dd, J = 10.9, 3.2 Hz, 2H), 3.89 – 3.74 (m, 1H), 3.67 (dd, J = 8.4, 5.2 Hz, 1H), 3.50 (dd, J = 8.4, 5.4 Hz, 1H), 3.41 (s, 1H), 3.31 (dt, J = 3.2, 1.5 Hz, 2H), 1.93 (d, J = 15.4 Hz, 3H), 1.60 – 1.42 (m, 5H), 1.23 (dd, J = 19.7, 11.6 Hz, 3H). ^{13}C NMR (126 MHz, CD_3OD) δ 172.33, 138.71, 137.83, 136.71, 128.19, 127.78, 127.64, 127.32, 127.02, 103.51, 101.25, 80.77, 76.03, 75.74, 73.07, 72.41, 71.81, 71.47, 70.61, 68.95, 68.21, 67.05, 66.94, 52.04, 50.11, 49.90, 30.50, 28.87, 27.76, 27.13, 26.76, 22.84, 21.80, 17.37, 12.71, 12.65, 9.61. HRMS (ESI) *m/z* calcd for $C_{41}H_{51}N_2O_{20}S_2$ [M-H]$^-$ 955.2482, found 955.2495.

5-Aminopentyl-O-α-L-idopyranosiduronate-(1→3)-4,6-di-O-sulfo-2-deoxy-2-acetamido-β-D-galactopyranoside (23)

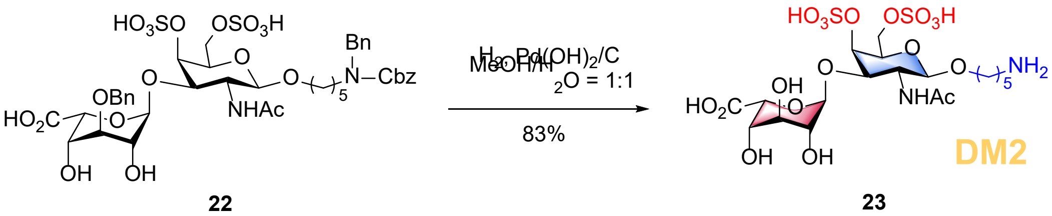

According to the produce for the conversion of compound **19** to **20**, a catalytic amount of 20% $Pd(OH)_2/C$ was added to a solution of compound **22** (8 mg, 0.008 mmol) in a mixture of $MeOH/H_2O$ (1:1 *v/v,* 1 mL). The solution was purged with H_2 and the suspension was stirred under an H_2 atmosphere for 1 h after which TLC indicated full conversion, filtered (celite), concentrated by reduced pressure to give the desired compound **23** (4 mg, 83%) as a white solid. ^1H NMR (500 MHz, D_2O) δ 4.69 (d, J = 4.7 Hz, 1H), 4.61 (s, 1H), 4.49 (d, J = 3.9 Hz, 1H), 4.45 (d, J = 7.7 Hz, 1H), 4.15 (dd, J = 11.3, 2.8 Hz, 1H), 4.10 – 4.02 (m, 1H), 3.99 – 3.87 (m, 3H), 3.77 (ddd, J = 16.7, 9.0, 5.1 Hz, 2H), 3.59 – 3.52 (m, 1H), 3.49 – 3.44 (m, 1H), 3.36 (dd, J = 7.8, 4.7 Hz, 1H), 2.84 (t, J = 7.6 Hz, 2H), 1.90 (s, 3H), 1.58 – 1.43 (m, 5H), 1.34 – 1.22 (m, 3H). ^{13}C NMR (126 MHz, D_2O) δ 175.73, 174.60, 102.87, 100.91, 76.02, 75.38, 72.34, 72.20, 71.45, 71.18, 70.32, 67.92, 51.77, 39.37, 28.02, 26.48, 22.11, 21.95. HRMS (ESI) *m/z* calcd for $C_{19}H_{33}N_2O_{18}S_2$ [M-H]$^-$ 641.1175, found 641.1173.

_N_-(benzyl)-benzyloxycarbonyl-5-aminopentyl-_O_-(Methyl-3-_O_-benzyl-2,4-di-_O_-sulfo-_α_-_L_-idopyranosiduronate)-(1→3)-4,6-_O_-benzylidene-2-deoxy-2-acetamido-_β_-_D_-galactopyranoside (24)

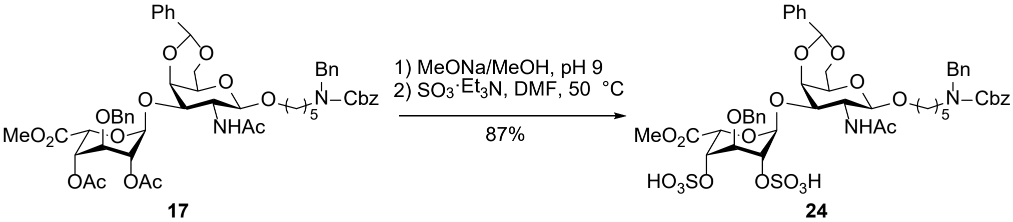

To a solution of **17** (50 mg, 0.05 mmol) in MeOH (1 mL) was adjusted to pH 9 by adding saturated MeONa in MeOH. The resultant solution was stirred at -10 ºC for 4 h after which TLC indicated full conversion and neutralized with Amberlite® IR-120 cation exchange resin (H+ form), filtered, concentrated under reduced pressure. The crude product (18 mg, 0.02 mmol) was re-dissolved in DMF (1 mL) and reacted with $SO_3·Et_3N$ (72.5 mg, 0.4 mmol, 20 eq.). The mixture was stirred at 50 ºC for 10 h after which TLC indicated full conversion, purified via Sephadex LH-20 gel filtration (MeOH)

to afford **24** (18 mg, 87%) as a syrup. ^{1}H NMR (500 MHz, CD_3OD) δ 7.44 – 7.13 (m, 20H), 5.40 (s, 1H), 5.35 (s, 1H), 5.14 (m, 3H), 4.76 (d, *J* = 11.7 Hz, 1H), 4.60 (d, *J* = 11.0 Hz, 2H), 4.50 (m, 3H), 4.39 (m, 1H), 4.25 – 4.07 (m, 4H), 3.89 – 3.68 (m, 2H), 3.53 (m, 1H), 3.39 (s, 3H), 3.34 (s, 1H), 1.95 (m, 2H), 1.46 (m, 4H). ^{13}C NMR (126 MHz, CD_3OD):*δ* 172.32, 169.78, 138.13, 137.96, 128.18, 127.93, 127.55, 127.28, 126.96, 125.79, 101.88, 100.27, 80.01, 74.66, 71.39, 71.05, 70.44, 69.02, 68.85, 67.01, 66.48, 66.29, 50.90, 50.79, 46.41, 28.80, 22.76, 21.78. HRMS (ESI) *m/z* calcd for $C_{49}H_{57}N_2O_{20}S_2$ [M-H]$^-$ 1057.2951, found 1057.2963.

***N*-(benzyl)-benzyloxycarbonyl-5-aminopentyl-*O*-(2,4-di-*O*-sulfo-3-*O*-benzyl-*α*-*L*-idopyranosidu-ronate)-(1→3)-4,6-*O*-benzylidene-2-deoxy-2-acetamido-*β*-*D*-galactopyranoside (25)**

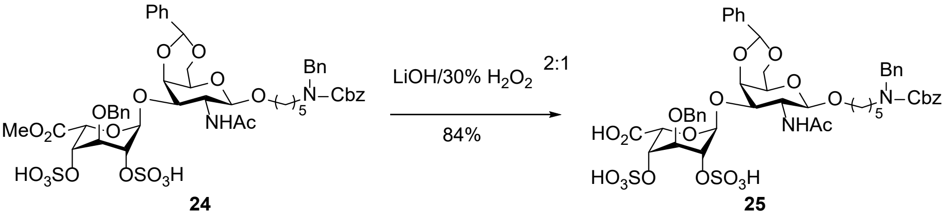

To a solution of **24** in a mixture of THF/MeOH (4:1 *v/v,* 1 mL) was added a solution of 1 M LiOH/30%H_2O_2 (2:1 *v/v,* 100 μL) at -10 ºC. The resultant solution was stirred at low temperature for 2 h after which TLC indicated full conversion and quenched by Amberlite® IR-120 cation exchange resin (H+ form), filtered, concentrated under reduced pressure. The residue was purified via Sephadex LH-20 gel filtration (MeOH) to afford **25** (37.8 mg, 84%) as a syrup. ^{1}H NMR (500 MHz, CD_3OD):*δ* 7.50 - 7.04 (m, 20H), 5.48 (d, *J* = 10.1 Hz, 1H), 5.35 (s, 1H), 5.15 (d, *J* = 19.5 Hz, 2H), 5.07 (s, 1H), 4.75 (d, *J* = 11.6 Hz, 1H), 4.70 (s, 1H), 4.60 (d, *J* = 11.6 Hz, 1H), 4.40 (d, *J* = 3.1 Hz, 1H), 4.33 (s, 1H), 4.22 - 4.16 (m, 1H), 4.09 (t, *J* = 12.5 Hz, 2H), 3.88 (d, *J* = 11.0 Hz, 1H), 3.79 (s, 1H), 3.68 (s, 1H), 3.53 (s, 1H), 3.44 (d, *J* = 30.9 Hz, 1H), 3.39 (s, 1H), 1.96 (m, 3H), 1.51 (d, *J* = 20.9 Hz, 4H), 1.32 (m, 2H). ^{13}C NMR (126 MHz, CD_3OD):*δ* 138.20, 138.05, 128.18, 128.08, 127.92, 127.87, 127.54, 127.50, 127.29, 126.87, 125.94, 125.77, 102.22, 101.58, 100.43, 74.74, 74.54, 71.45, 71.09, 70.43, 68.92, 66.31, 50.97, 48.04, 47.87, 28.80, 22.76, 21.82. HRMS (ESI) *m/z* calcd for $C_{48}H_{55}N_2O_{20}S_2$ [M-H]$^-$ 1043.2795, found 1043.2788.

5-Aminopentyl-*O*-(2,4-di-*O*-sulfo-*α*-*L*-idopyranosiduronate)-(1→3)-2-deoxy-2-acetamido-*β*-*D*-galactopyranoside (26)

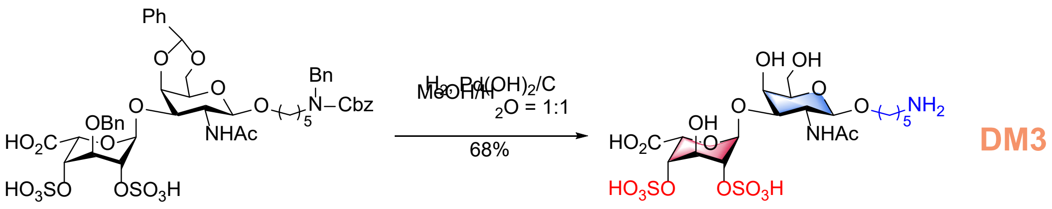

25 **26**

According to the produce for the conversion of compound **19** to **20**, a catalytic amount of 20% $Pd(OH)_2/C$ was added to a solution of compound **25** (17 mg, 0.016 mmol) in a mixture of $MeOH/H_2O$ (1:1 *v/v,* 1 mL). The solution was purged with H_2 and the suspension was stirred under an H_2 atmosphere for 1 h after which TLC indicated full conversion, filtered (celite), concentrated by reduced pressure to give the desired compound **26** (7 mg, 68%) as a white solid. ^1H NMR (500 MHz, D_2O) δ 5.17 (s, 1H), 4.61 (s, 1H), 4.55 (s, 1H), 4.50 (m, 1H), 4.13 (m, 1H), 3.97 (m, 1H), 3.87 – 3.75 (m, 3H), 3.74 – 3.57 (m, 5H), 2.93 (s, 2H), 2.01 (s, 3H), 1.58 (m, 4H), 1.35 (m, 2H). ^{13}C NMR (126 MHz, D_2O) δ 174.88, 174.60, 162.57, 101.24, 100.85, 79.64, 74.93, 74.27, 72.83, 70.06, 67.75, 66.42, 65.94, 60.91, 51.36, 39.42, 28.06, 26.52, 23.36, 22.40, 22.11. HRMS (ESI) *m/z* calcd for $C_{41}H_{51}N_2O_{20}S_2$ $[M-H]^-$ 955.2482, found 955.2495. HRMS (ESI) *m/z* calcd for $C_{19}H_{33}N_2O_{18}S_2$ $[M-H]^-$ 641.1175, found 641.1182.

***N*-(benzyl)-benzyloxycarbonyl-5-aminopentyl-*O*-(2,4-di-*O*-sulfo-3-*O*-benzyl-*α*-*L*-idopyrano-siduronate)-(1→3)-4,6-di-*O*-sulfo-2-deoxy-2-acetamido-*β*-*D*-galactopyranoside (27)**

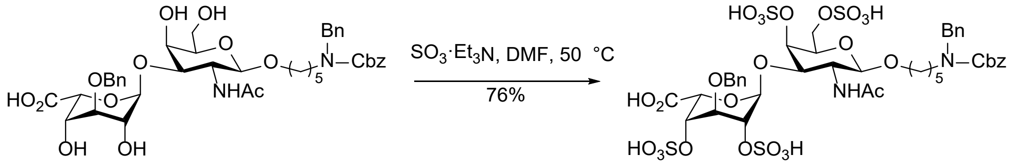

19 **27**

According to the produce for the conversion of compound **18** to compound **21**, compound **19** (22 mg, 0.027 mmol) in DMF (1 mL) was treated with sulfur trioxide-triethylamine complex ($SO_3 \cdot Et_3N$) (98 mg, 0.54 mmol, 20 eq.). The mixture was stirred at 50 ºC for 10 h after which TLC indicated full conversion and purified via Sephadex LH-20 gel filtration (MeOH) to give desired compound **27** (23 mg, 76%) as a syrup. ^1H NMR (500 MHz, CD_3OD) δ 7.51 – 7.13 (m, 15H), 5.47 (s, 1H), 5.31 (d, J = 9.4 Hz, 1H), 5.15 (d, J = 21.1 Hz, 2H), 4.83 – 4.73 (m, 3H), 4.69 (d, J = 12.3 Hz, 1H), 4.51 (s, 2H),

4.46 (s, 1H), 4.43 – 4.26 (m, 4H), 4.05 (s, 1H), 3.91 (d, J = 5.5 Hz, 1H), 3.89 – 3.75 (m, 2H), 3.42 (d, J = 29.9 Hz, 1H), 3.22 (s, 2H), 1.99 (d, J = 15.4 Hz, 3H), 1.52 (s, 4H), 1.30 (s, 4H). ^{13}C NMR (126 MHz, CD_3OD) δ 172.73, 138.32, 128.21, 127.96, 127.61, 126.97, 102.60, 101.70, 75.17, 74.46, 73.12, 71.42, 71.19, 69.01, 68.23, 66.95, 51.54, 47.94, 47.76, 47.59, 47.42, 47.25, 46.51, 28.84, 27.03, 22.81, 22.20, 7.88. HRMS (ESI) *m/z* calcd for $C_{41}H_{51}N_2O_{26}S_4$ [M-H]$^-$ 1115.1618, found 1115.1625.

5-Aminopentyl-*O*-(2,4-di-*O*-sulfo-*α*-*L*-idopyranosiduronate)-(1→3)-4,6-di-*O*-sulfo-2-deoxy-2-acetamido-*β*-*D*-galactopyranoside (28)

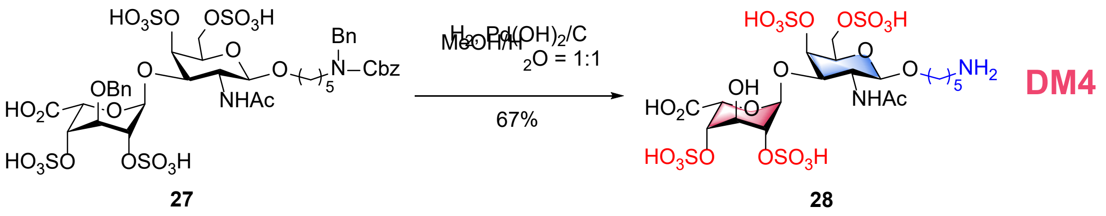

According to the produce for the conversion of compound **19** to **20**, a catalytic amount of 20% $Pd(OH)_2/C$ was added to a solution of compound **27** (14 mg, 0.013 mmol) in a mixture of MeOH/H_2O (1:1 *v/v,* 1 mL). The solution was purged with H_2 and the suspension was stirred under an H_2 atmosphere for 1 h after which TLC indicated full conversion, filtered (celite), concentrated by reduced pressure to give the desired compound **28** (6.7 mg, 67%) as a white solid. ^{1}H NMR (500 MHz, D_2O) δ 5.12 (s, 1H), 4.80 (s, 1H), 4.60 (s, 1H), 4.51 (s, 1H), 4.46 (d, J = 4.8 Hz, 1H), 4.28 (s, 1H), 4.15 (dd, J = 11.4, 2.9 Hz, 1H), 4.07 (t, J = 10.0 Hz, 2H), 3.95 (s, 3H), 3.76 (dt, J = 12.0, 6.0 Hz, 1H), 3.58 – 3.51 (m, 1H), 2.85 (t, J = 7.6 Hz, 2H), 1.93 (s, 3H), 1.58 – 1.44 (m, 4H), 1.33 – 1.23 (m, 2H). ^{13}C NMR (126 MHz, D_2O) δ 175.05, 174.33, 161.70, 100.96, 100.14, 75.92, 75.50, 74.22, 72.13, 70.35, 67.85, 66.43, 66.01, 51.93, 39.34, 28.02, 26.35, 22.43, 21.98. HRMS (ESI) *m/z* calcd for $C_{19}H_{33}N_2O_{24}S_4$ [M-H]$^-$ 801.0311, found 801.0305.

6. NMR Spectra of Compounds and Glycopolymers

^{1}H NMR spectrum ($CDCl_3$, 500 MHz) of compound 1

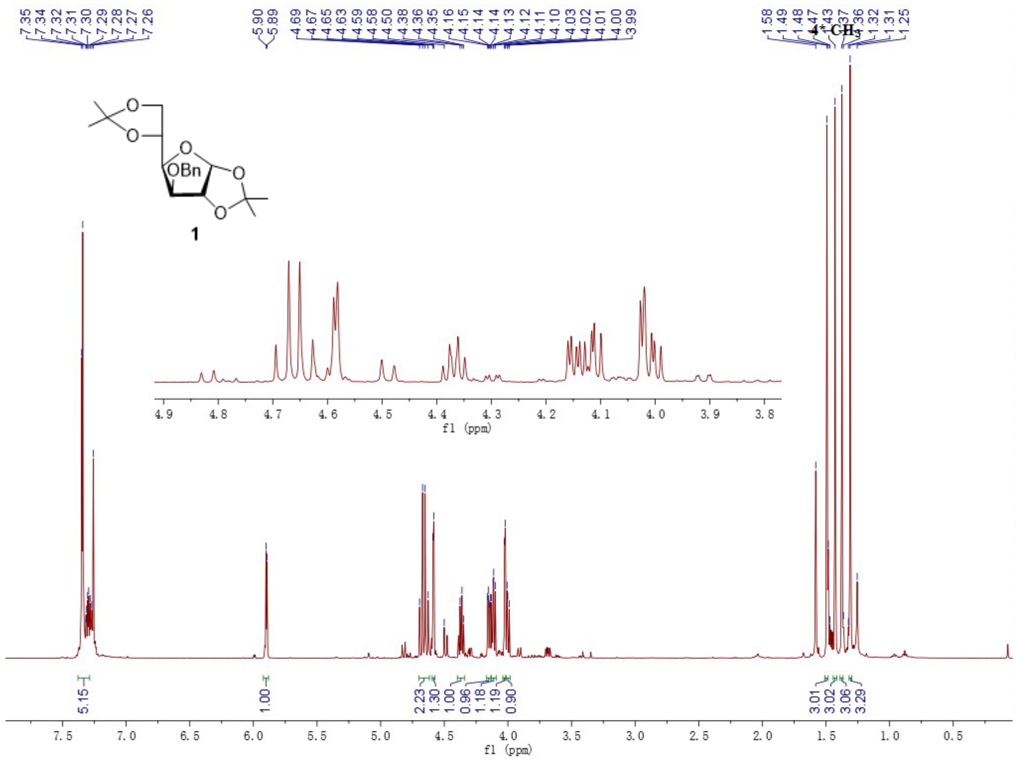

^{1}H NMR spectrum ($CDCl_3$, 500 MHz) of compound 3

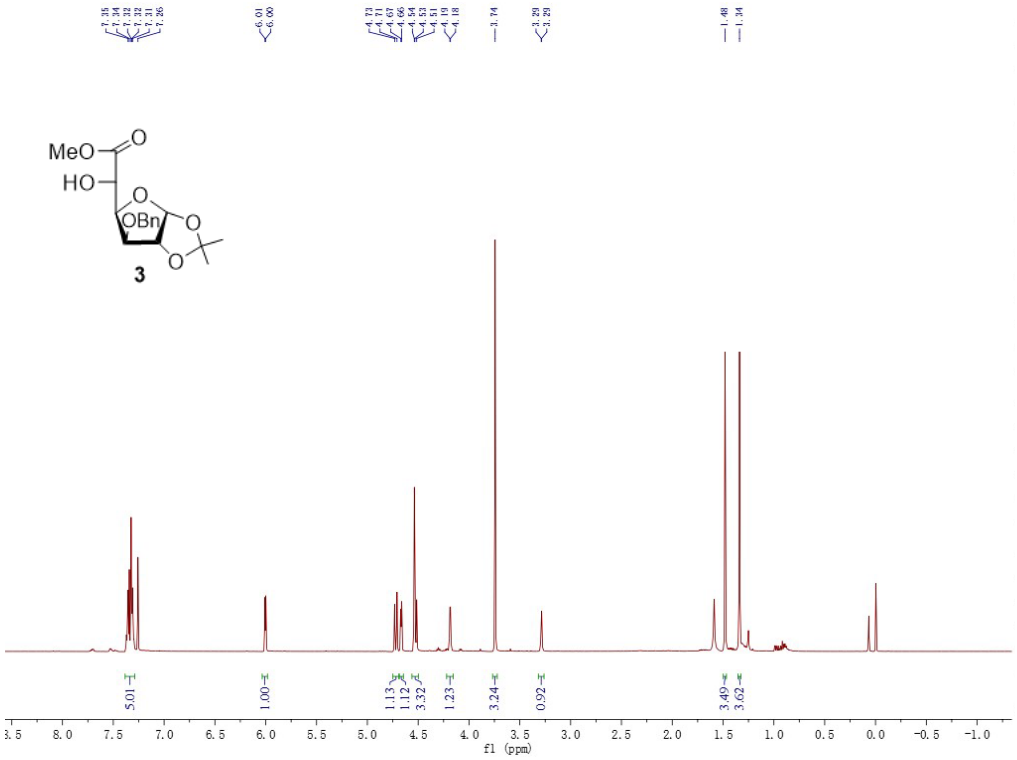

^{1}H NMR spectrum ($CDCl_3$, 500 MHz) of compound 5

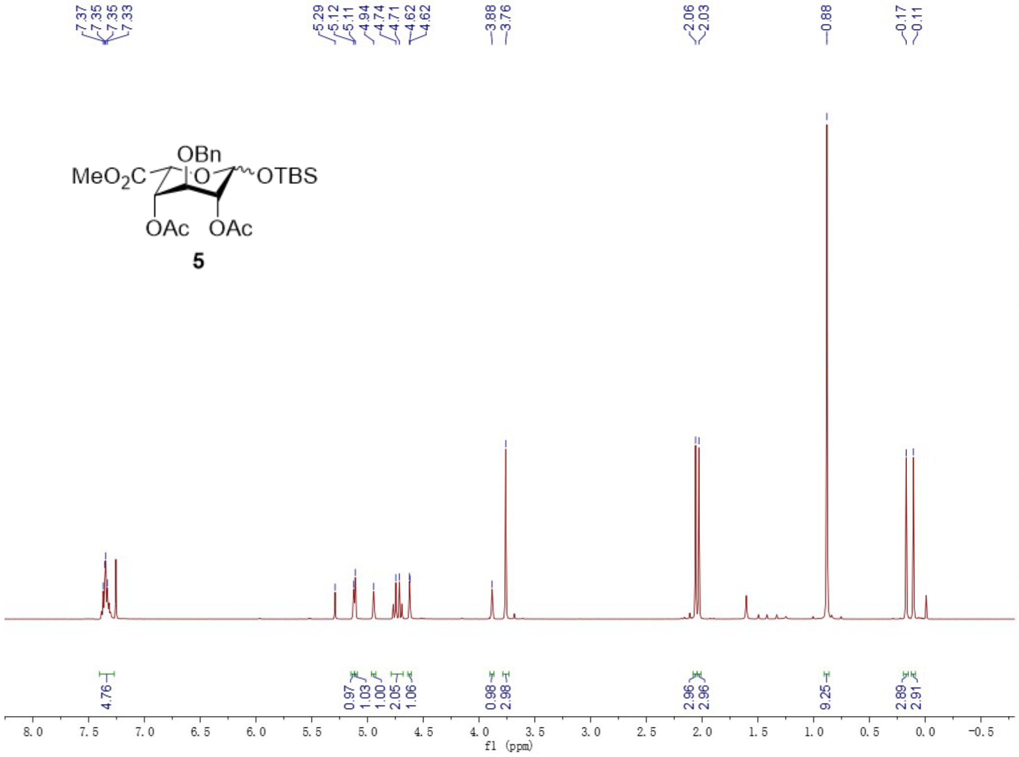

^{13}C NMR spectrum ($CDCl_3$, 126 MHz) of compound 5

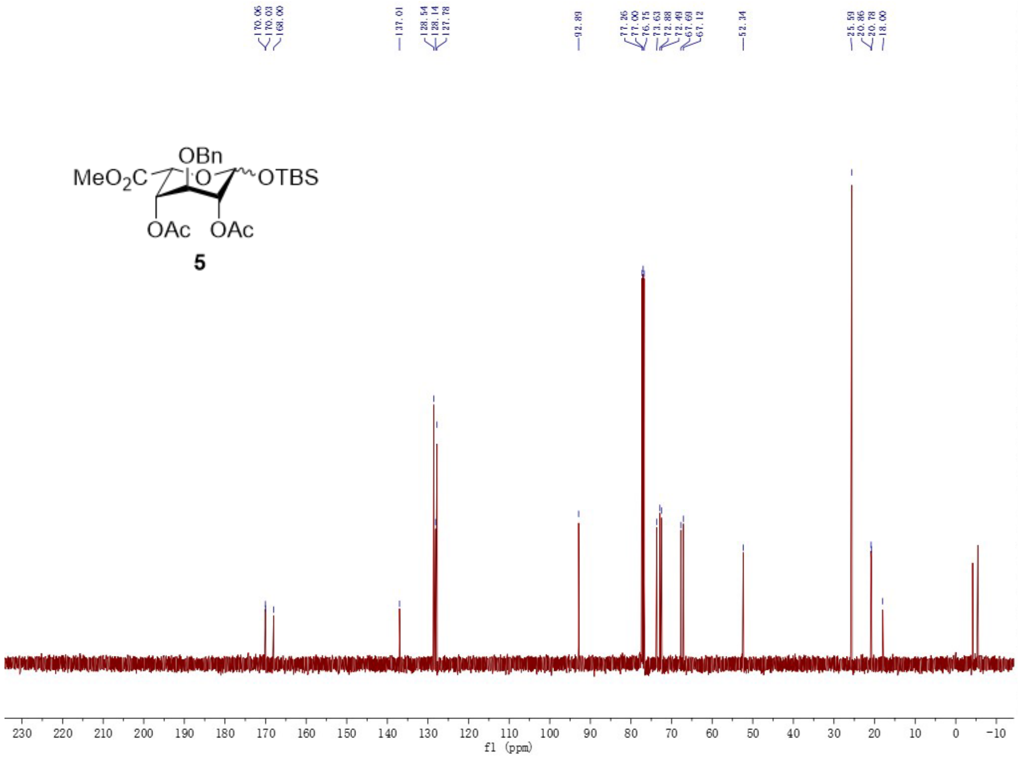

^{1}H NMR spectrum ($CDCl_3$, 500 MHz) of compound 6

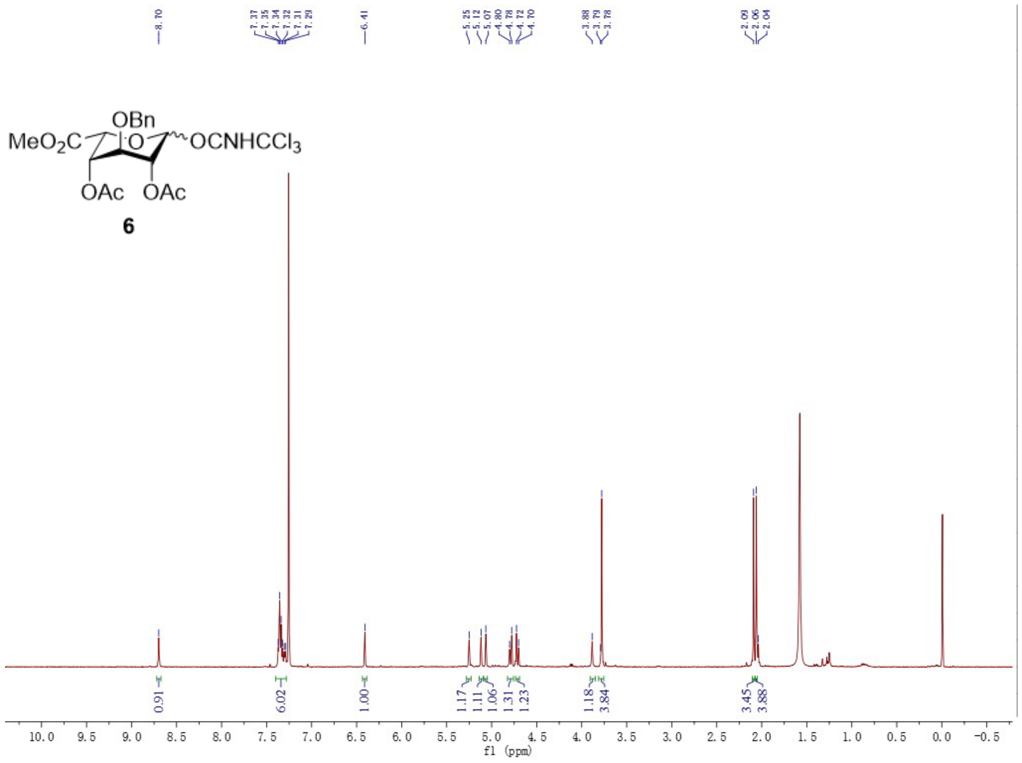

^{13}C NMR spectrum ($CDCl_3$, 126 MHz) of compound 6

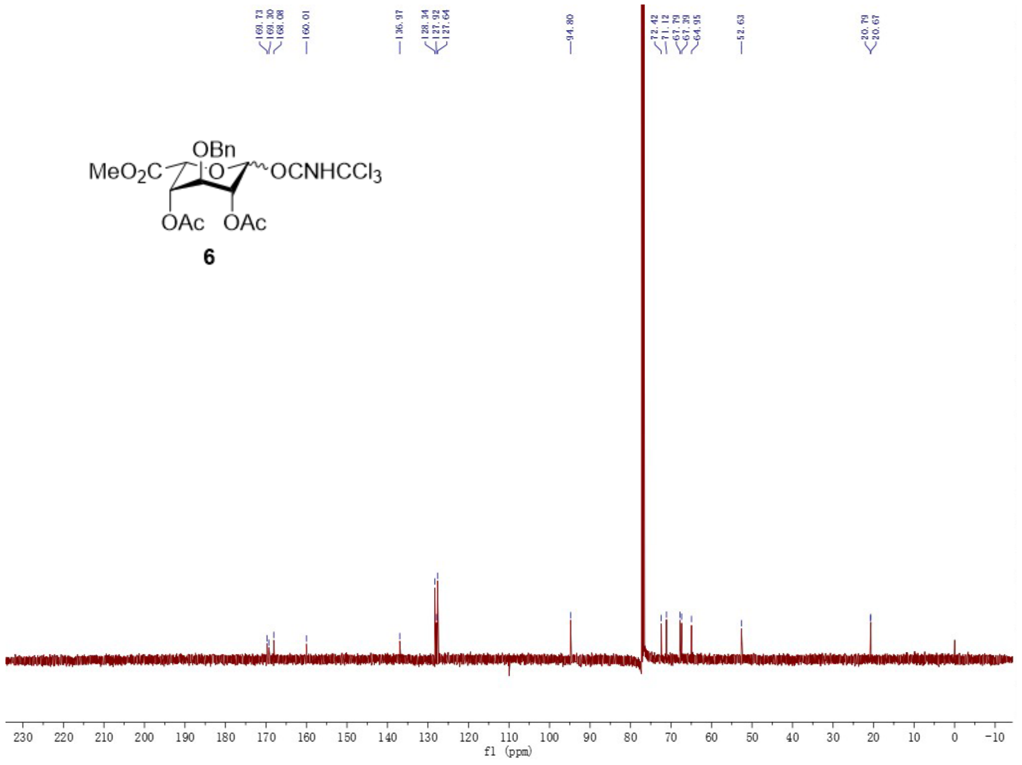

^{1}H NMR spectrum ($CDCl_3$, 500 MHz) of compound 7

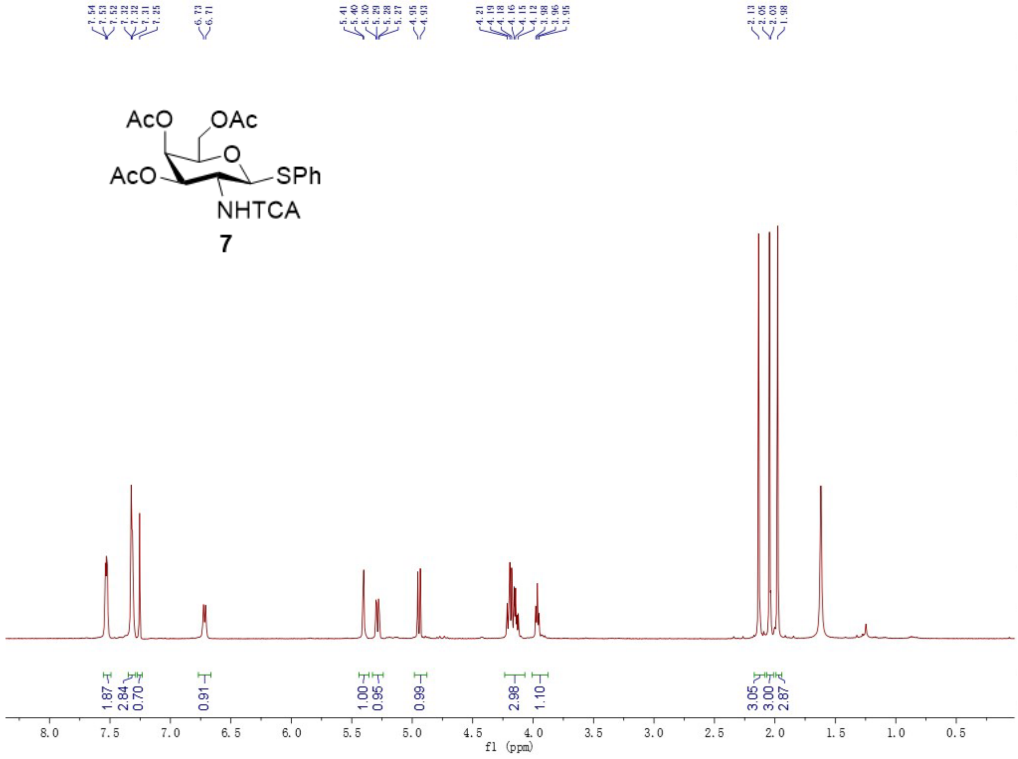

^{13}C NMR spectrum ($CDCl_3$, 126 MHz) of compound 7

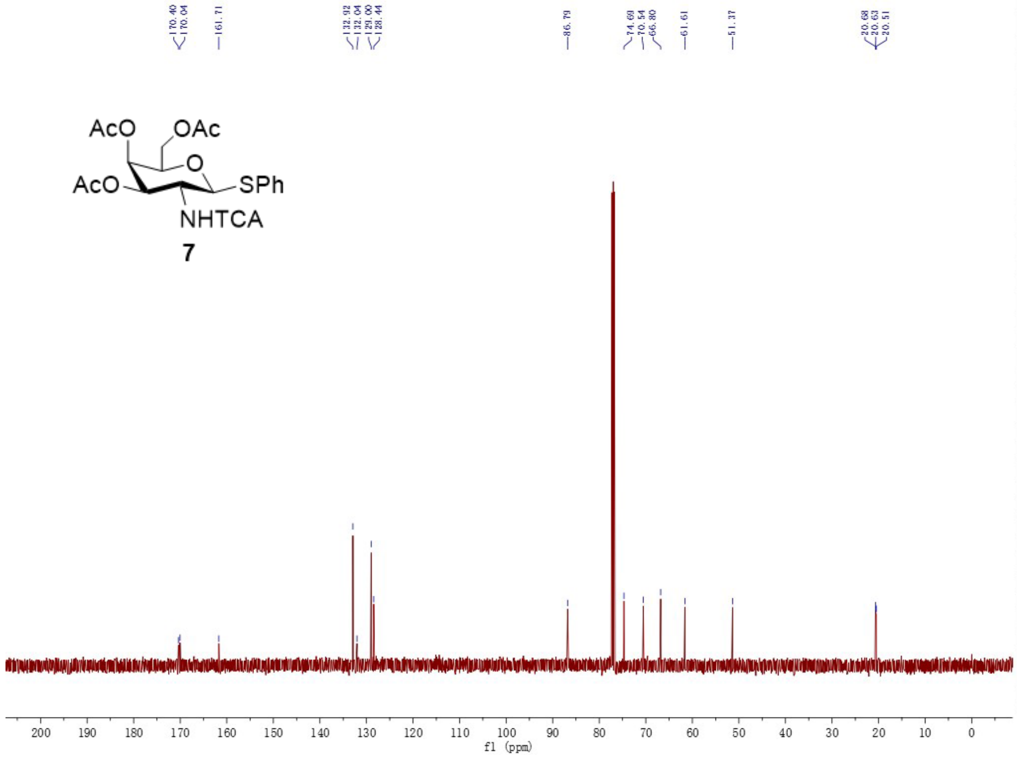

^{1}H NMR spectrum ($CDCl_3$, 500 MHz) of compound 8

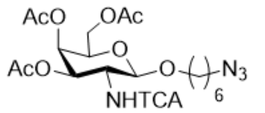

8

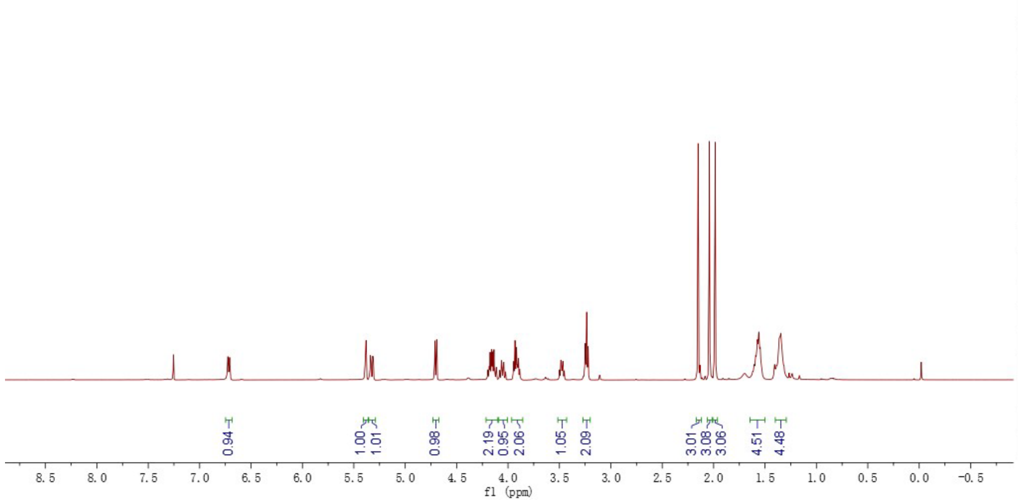

^{13}C NMR spectrum ($CDCl_3$, 126 MHz) of compound 8

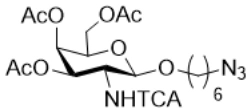

8

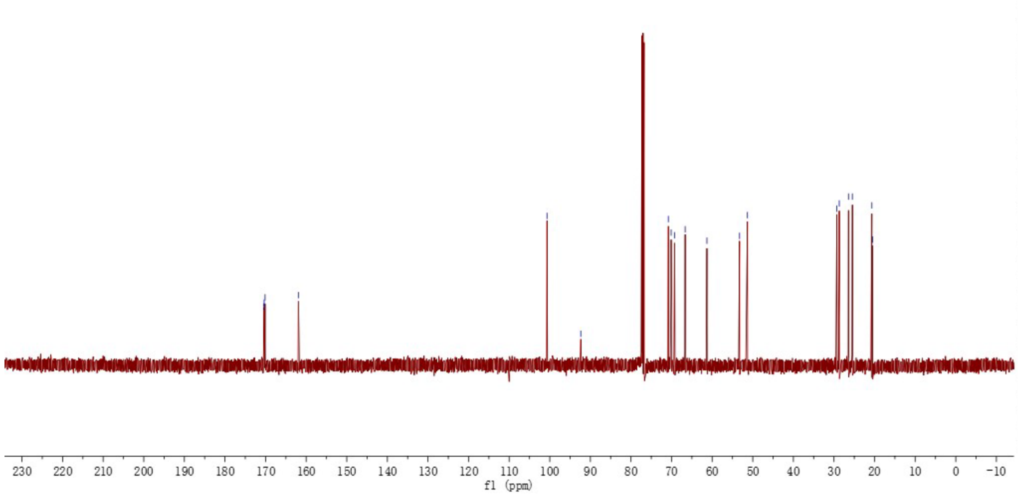

^{1}H NMR spectrum ($CDCl_3$, 500 MHz) of compound 9

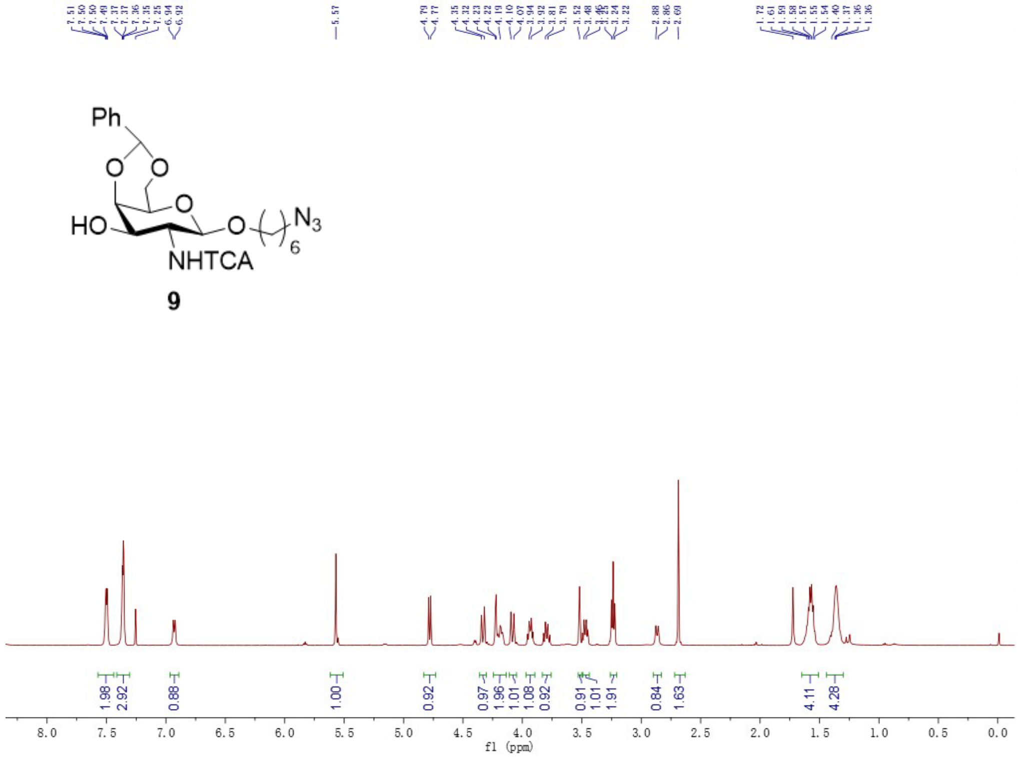

^{13}C NMR spectrum ($CDCl_3$, 126 MHz) of compound 9

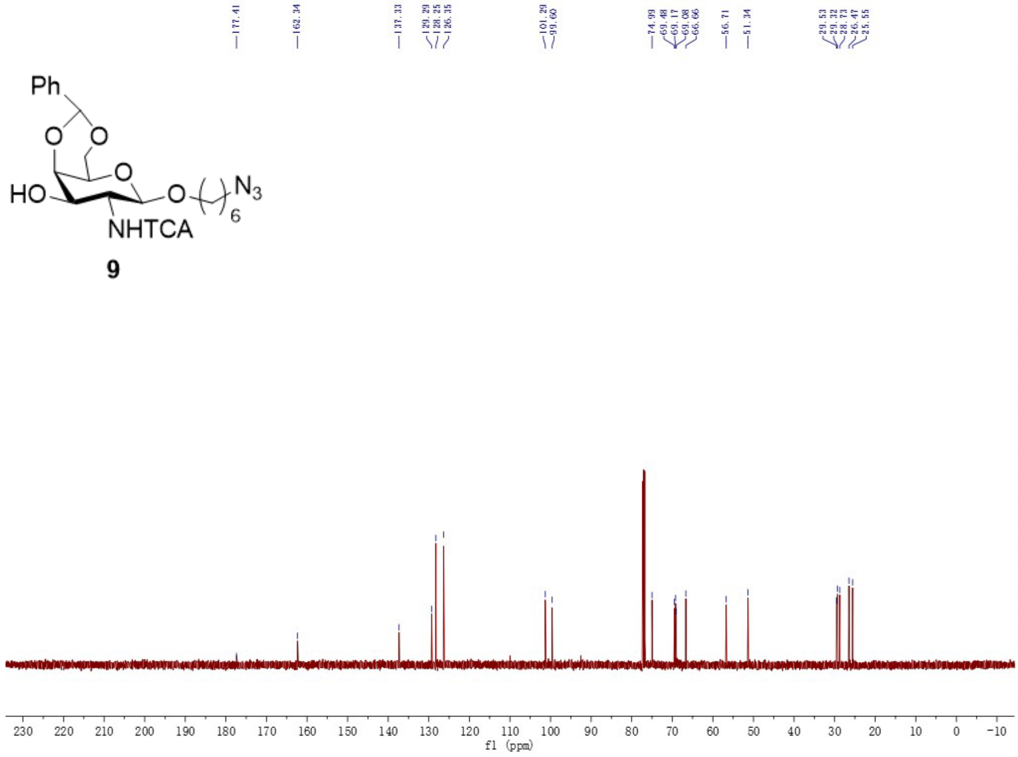

^{1}H NMR spectrum ($CDCl_3$, 500 MHz) of compound 10

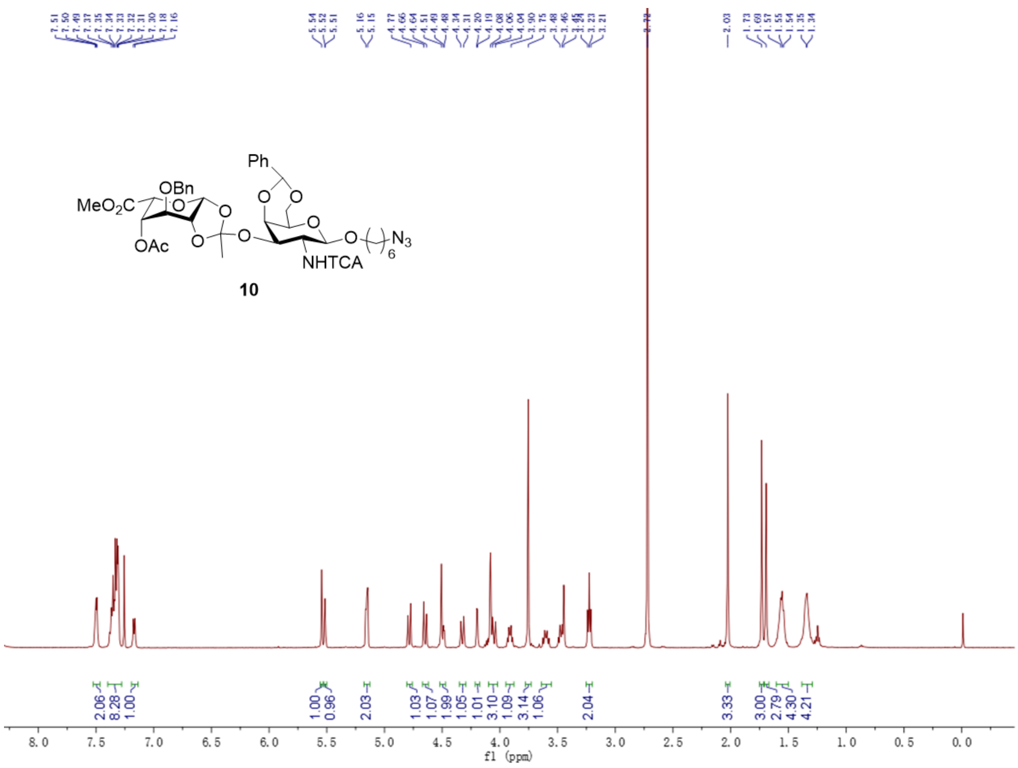

^{13}C NMR spectrum ($CDCl_3$, 126 MHz) of compound 10

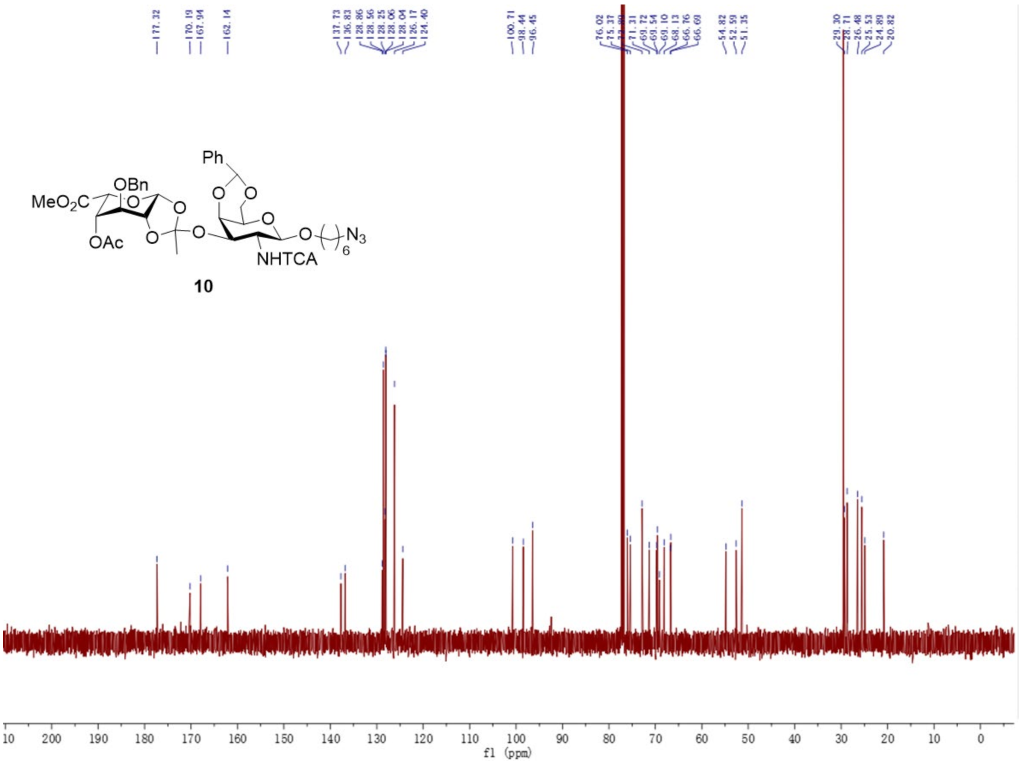

^{1}H-^{1}H COSY NMR spectrum ($CDCl_3$, 500 MHz) of compound 10

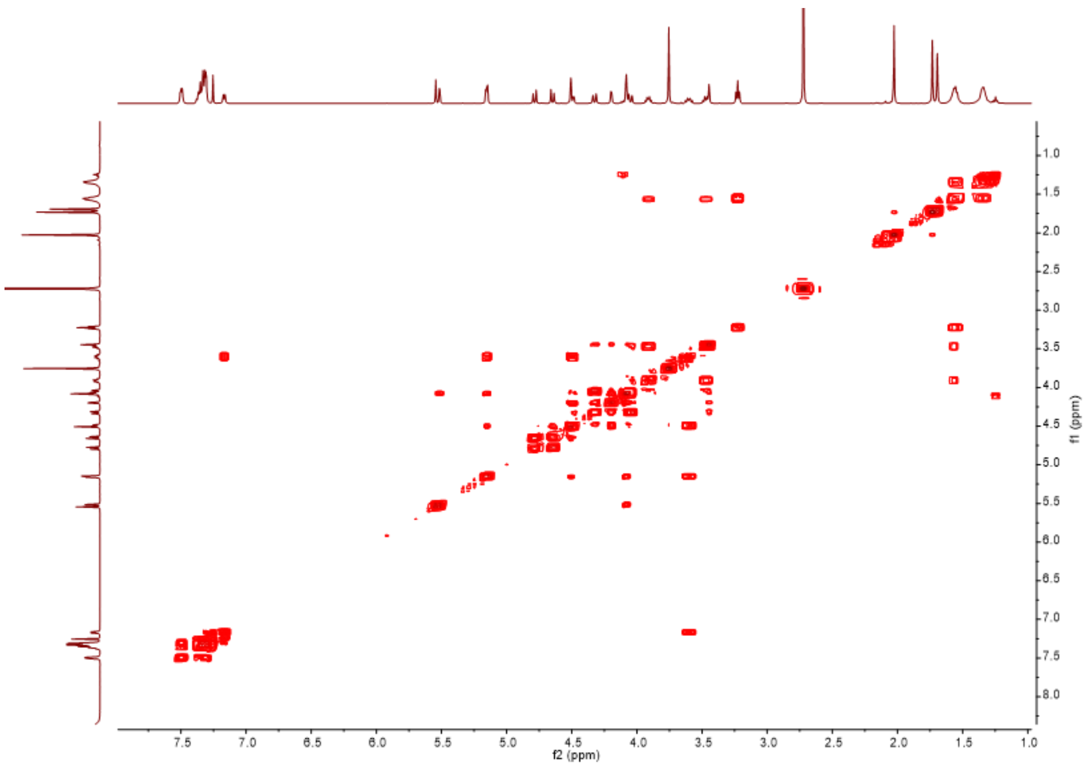

^{1}H-^{13}C HSQC NMR spectrum ($CDCl_3$, 500 MHz) of compound 10

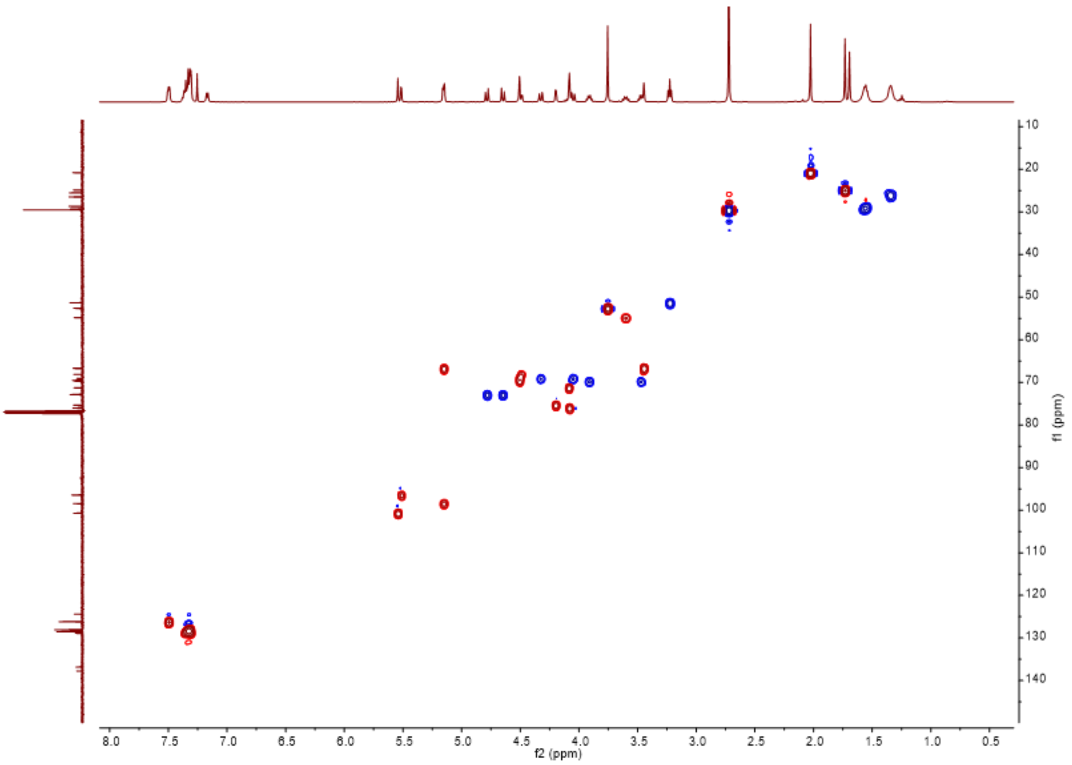

^{1}H NMR spectrum (CDCl$_3$, 500 MHz) of compound 11

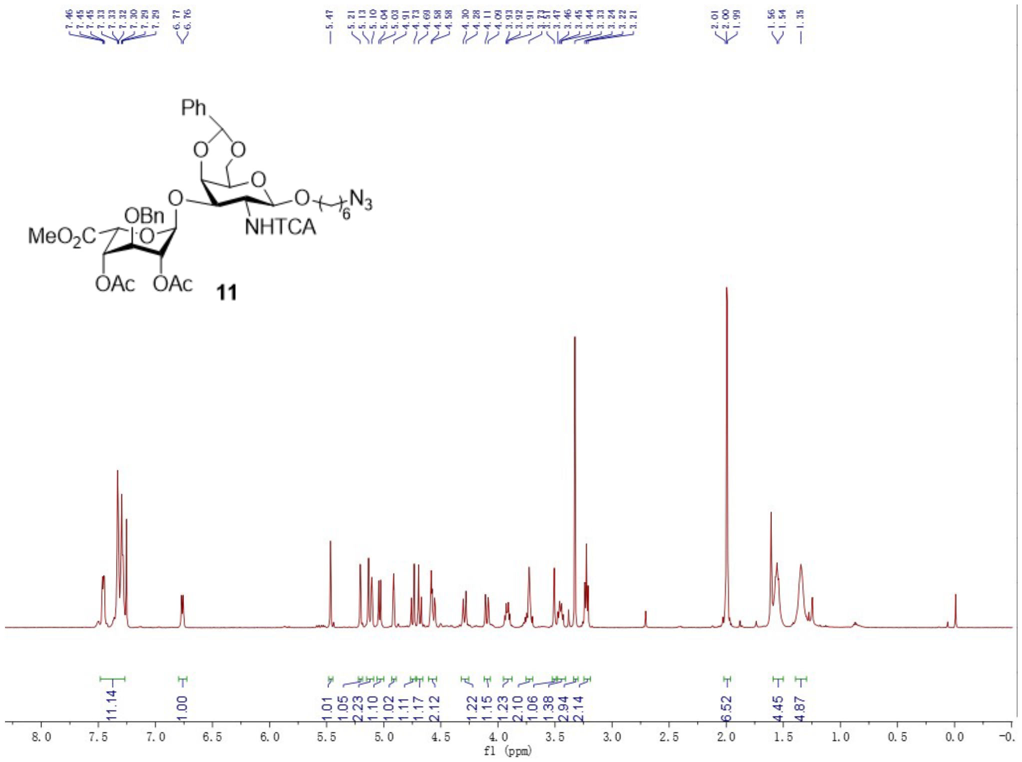

^{13}C NMR spectrum (CDCl$_3$, 126 MHz) of compound 11

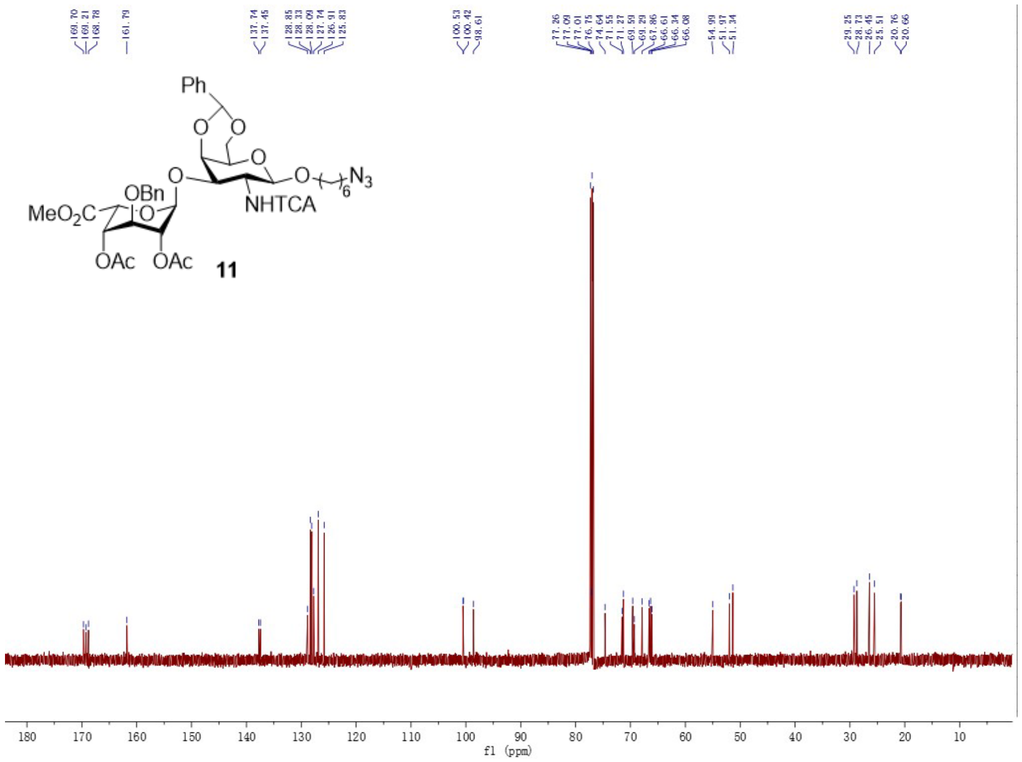

^{1}H-^{1}H COSY NMR spectrum ($CDCl_3$, 500 MHz) of compound 11

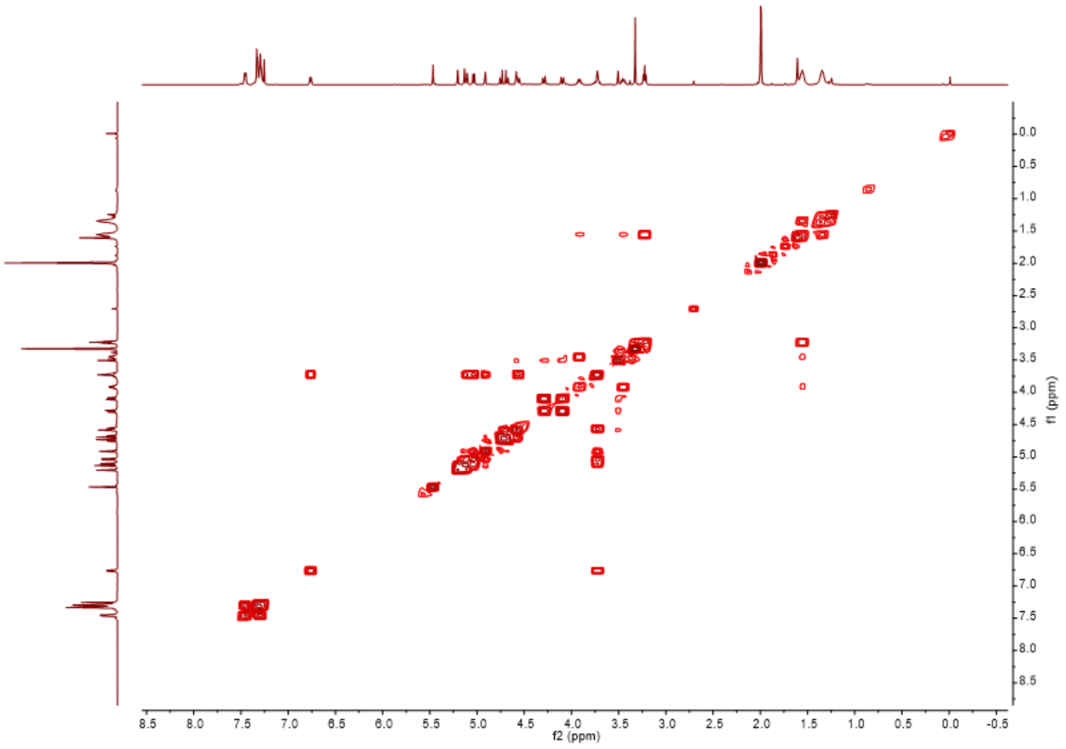

^{1}H-^{13}C HSQC NMR spectrum ($CDCl_3$, 500 MHz) of compound 11

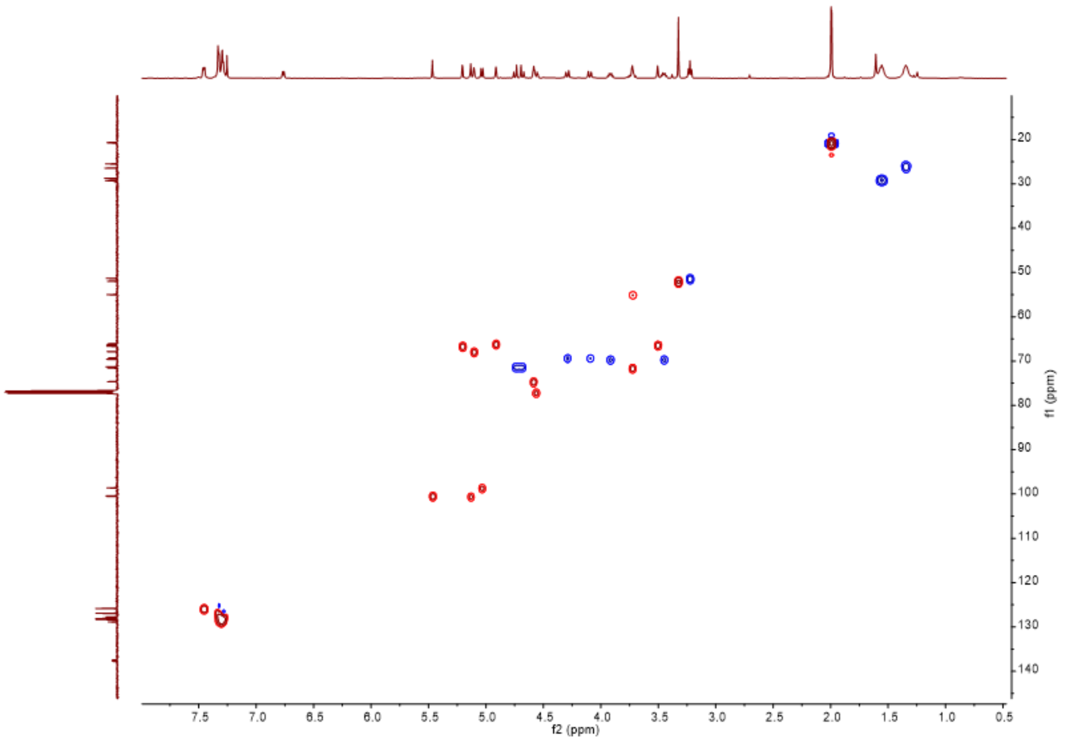

^{1}H NMR spectrum ($CDCl_3$, 500 MHz) of compound 13

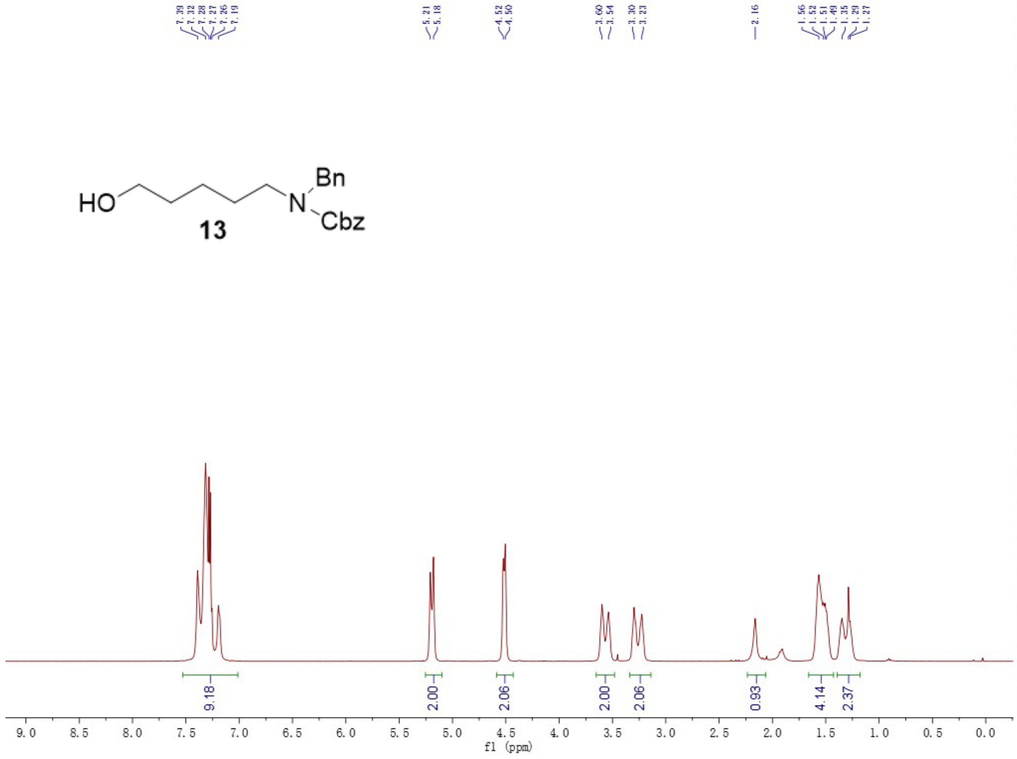

^{1}H NMR spectrum ($CDCl_3$, 500 MHz) of compound 14

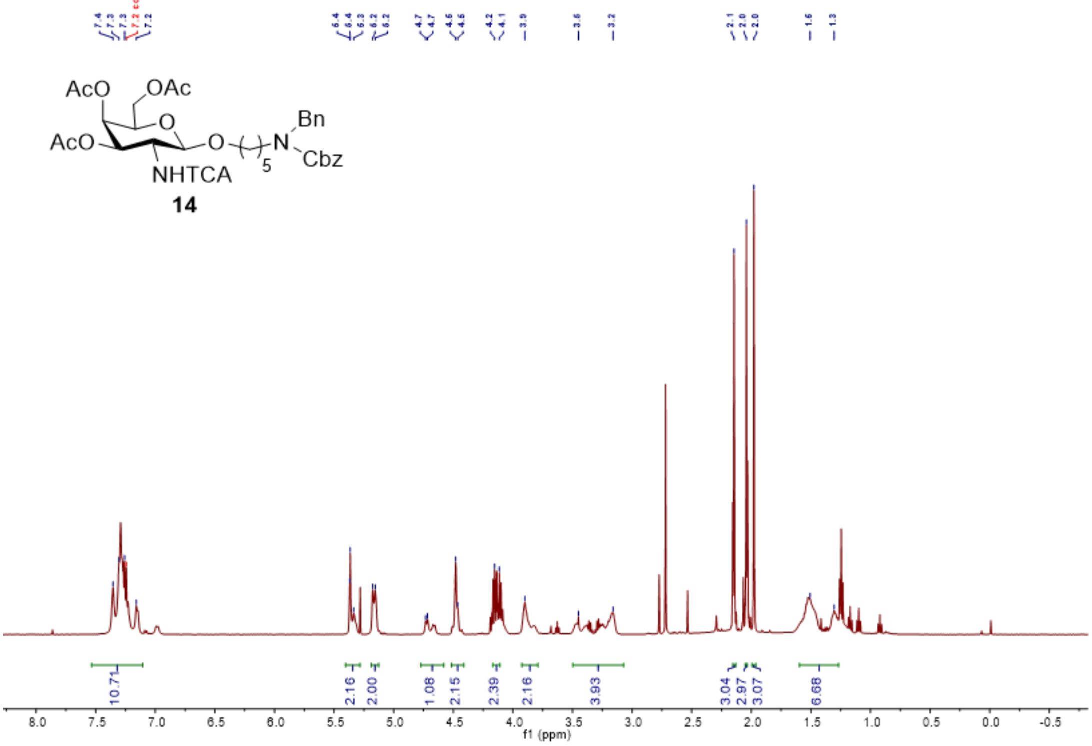

^{13}C NMR spectrum ($CDCl_3$, 126 MHz) of compound 14

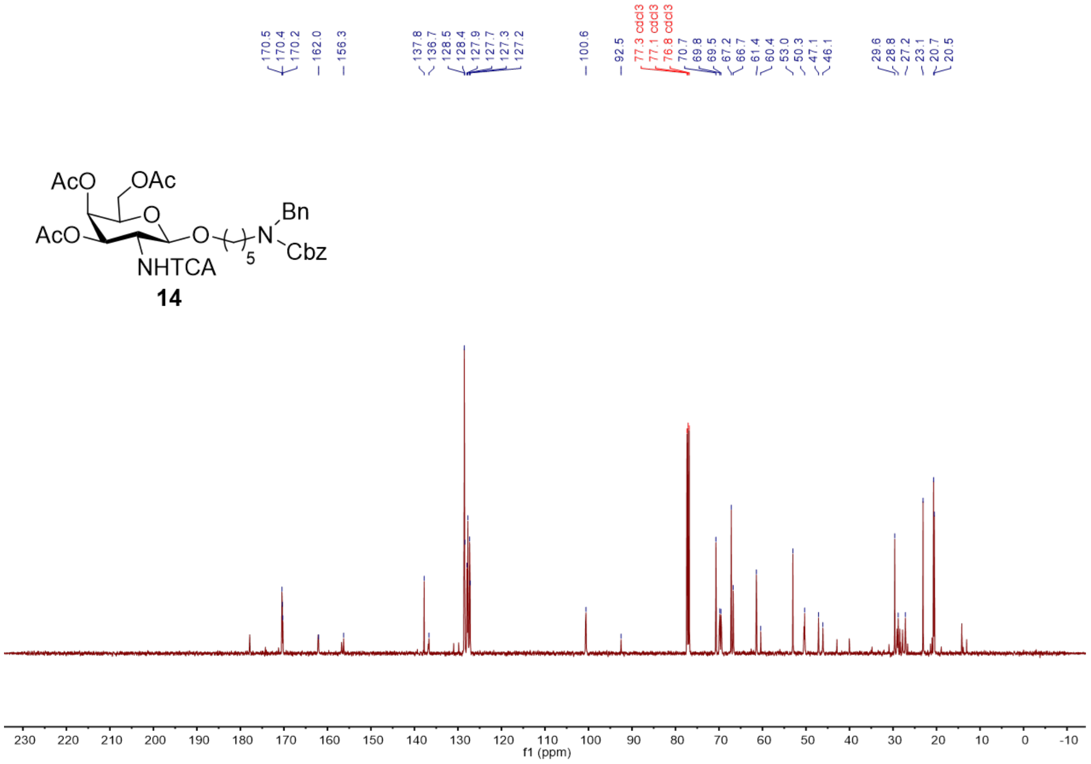

^{1}H NMR spectrum ($CDCl_3$, 500 MHz) of compound 15

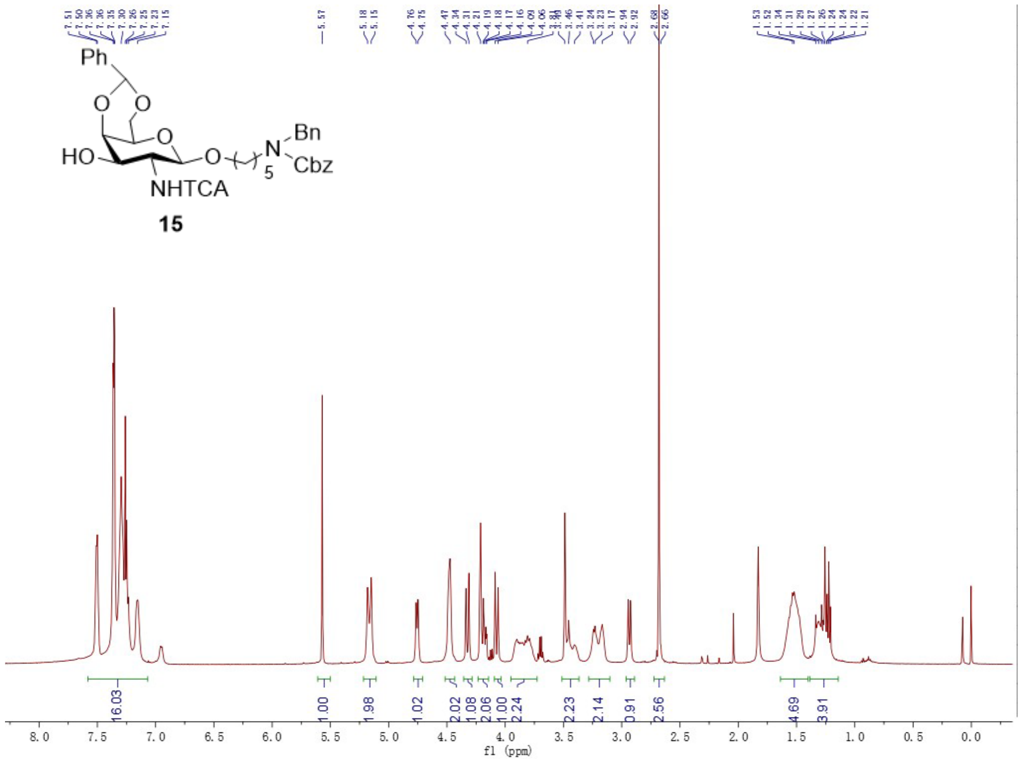

^{13}C NMR spectrum ($CDCl_3$, 126 MHz) of compound 15

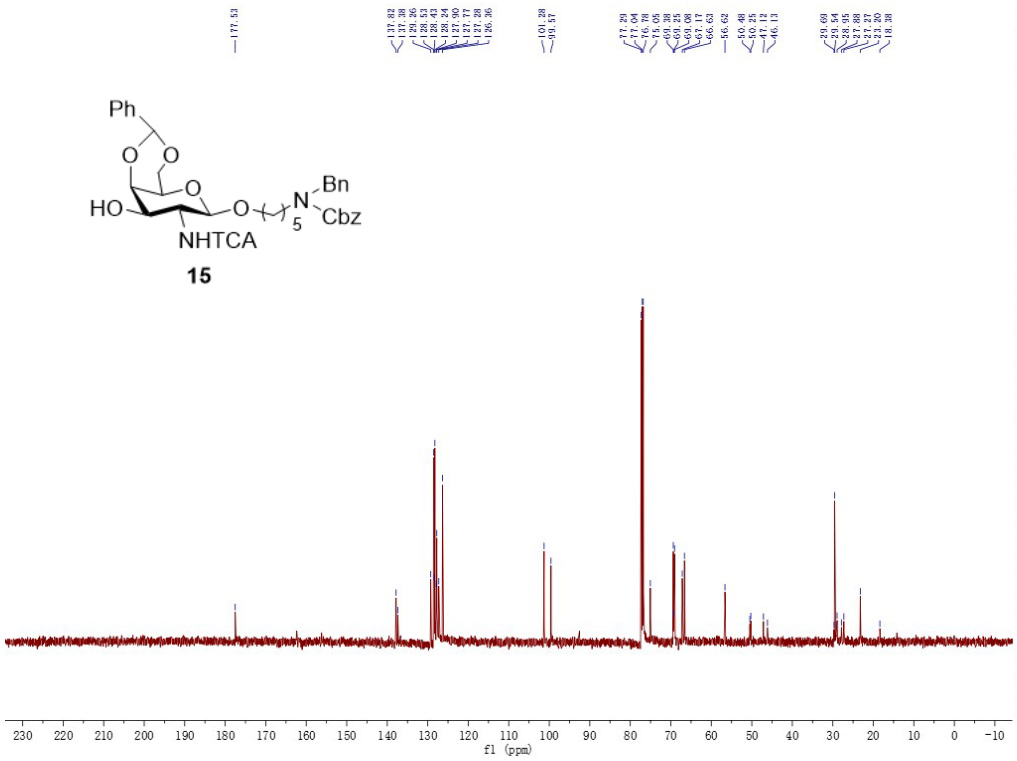

^{1}H-^{1}H COSY NMR spectrum ($CDCl_3$, 500 MHz) of compound 15

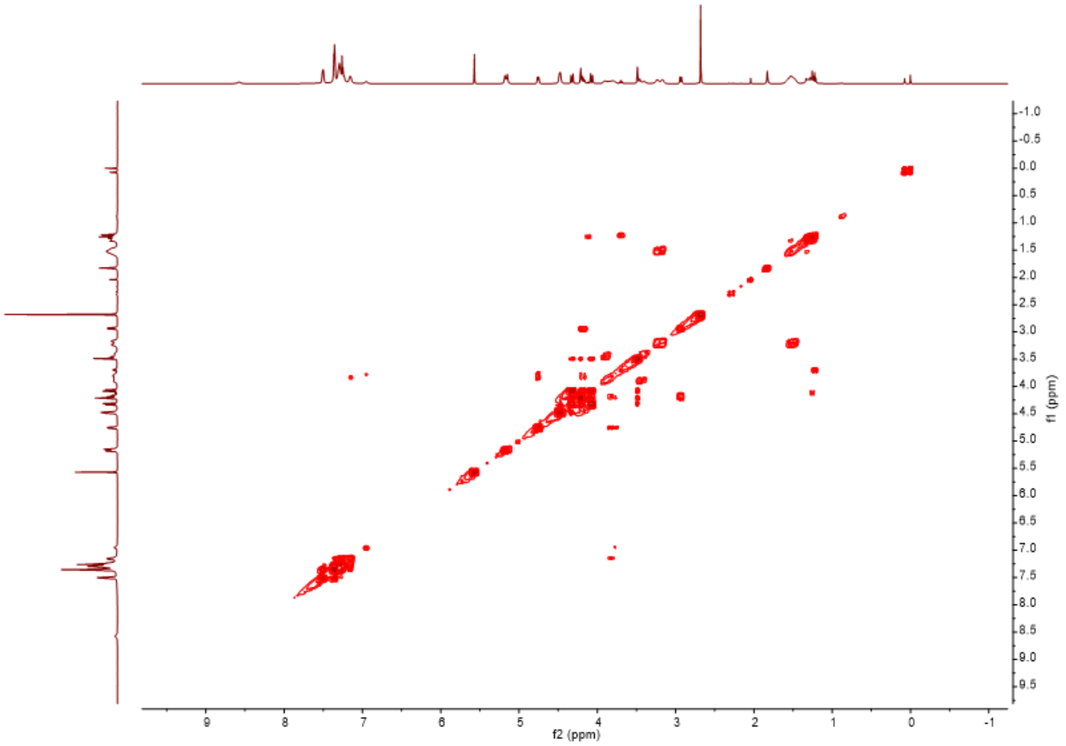

^{1}H-^{13}C HSQC NMR spectrum ($CDCl_3$, 500 MHz) of compound 15

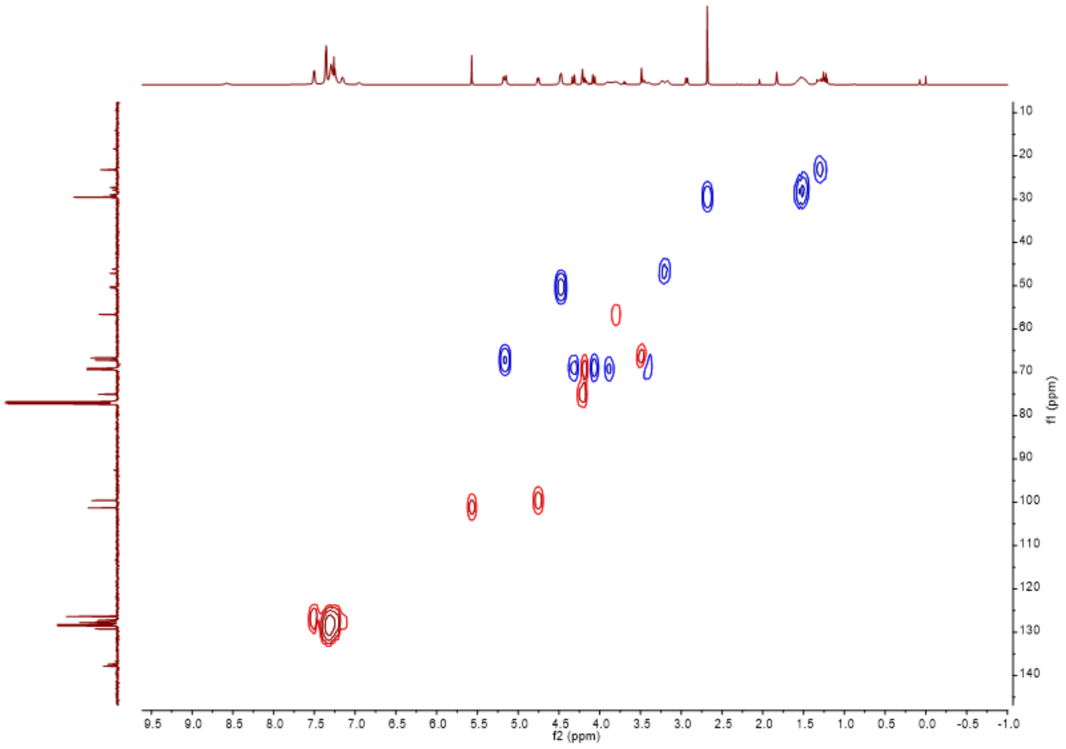

^{1}H NMR spectrum ($CDCl_3$, 500 MHz) of compound 16

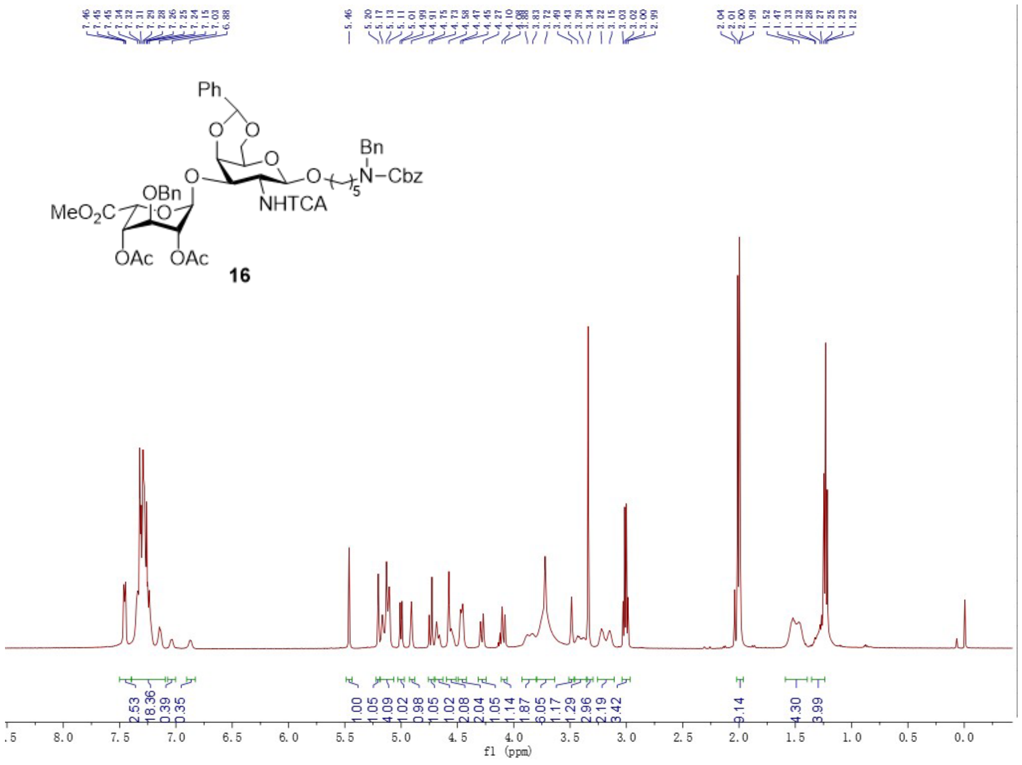

^{13}C NMR spectrum ($CDCl_3$, 126 MHz) of compound 16

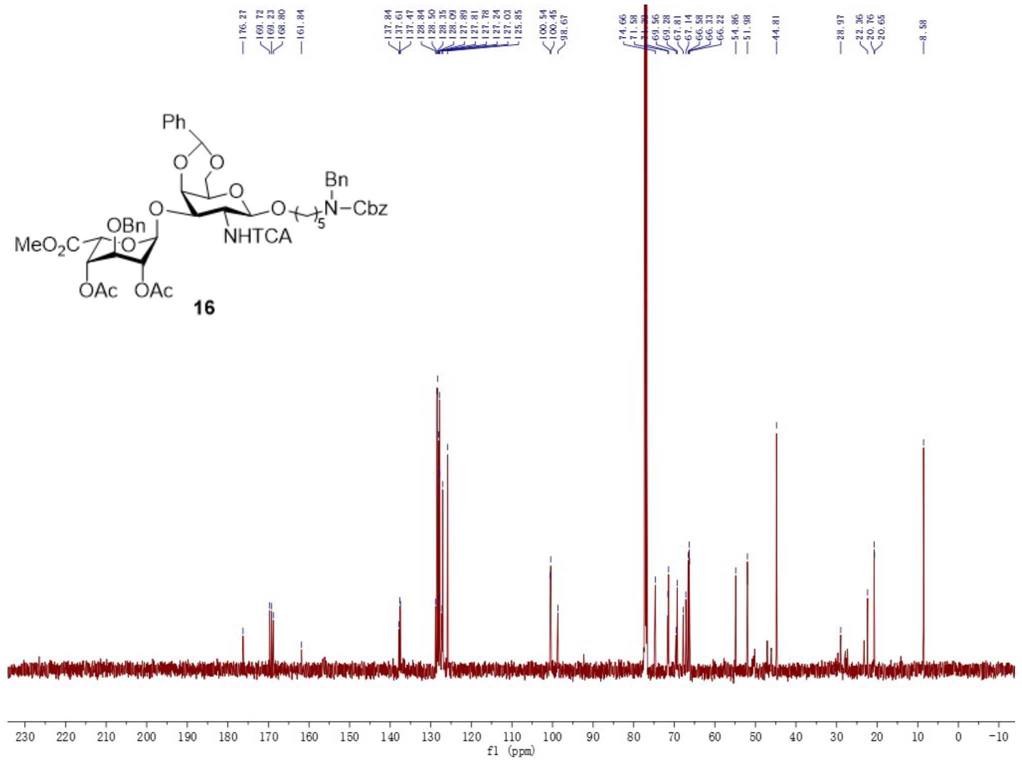

^{1}H-^{1}H COSY NMR spectrum ($CDCl_3$, 500 MHz) of compound 16

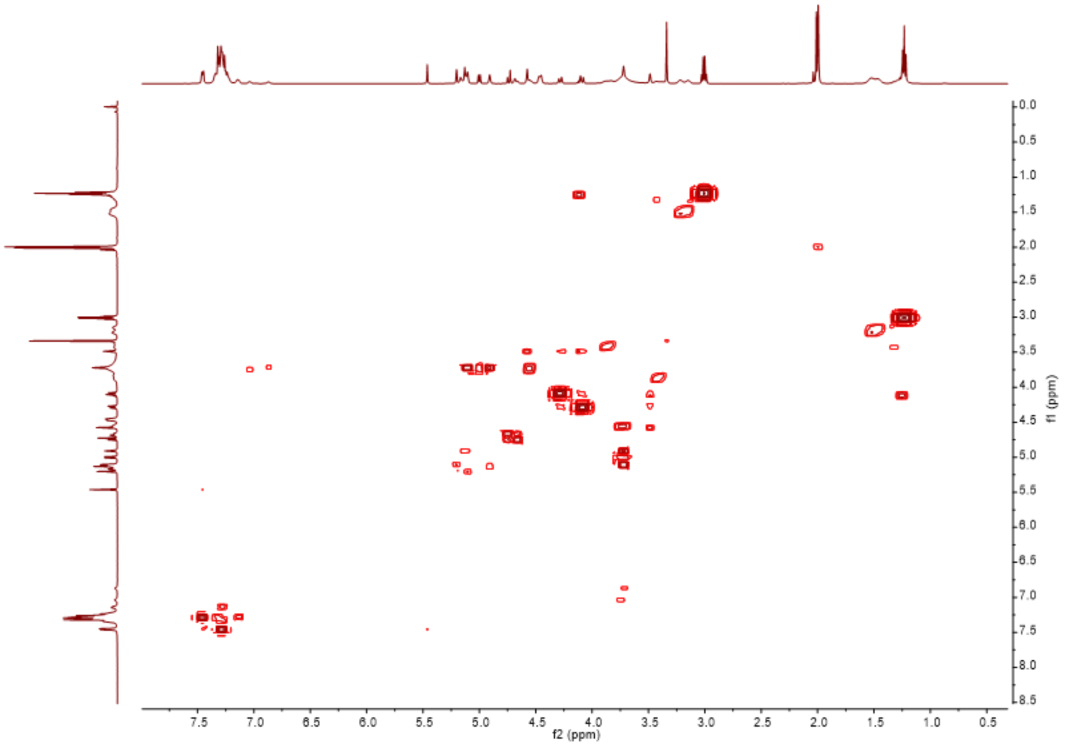

^{1}H-^{13}C HSQC NMR spectrum ($CDCl_3$, 500 MHz) of compound 16

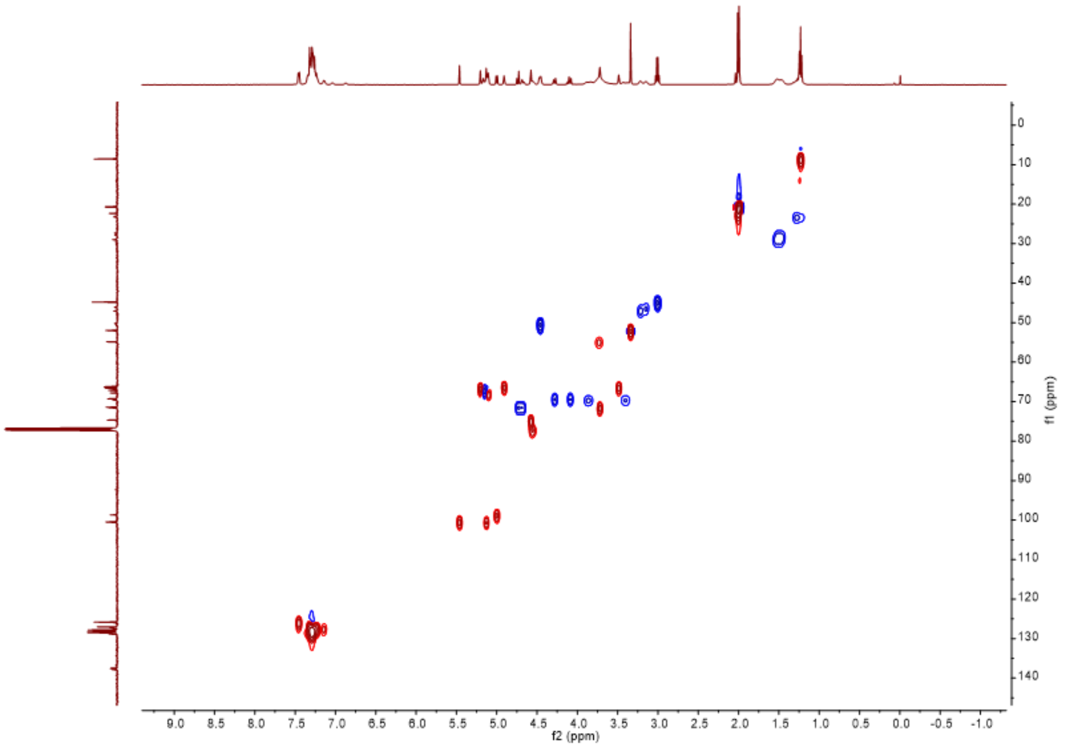

^{1}H-^{13}C HMBC NMR spectrum ($CDCl_3$, 500 MHz) of compound 16

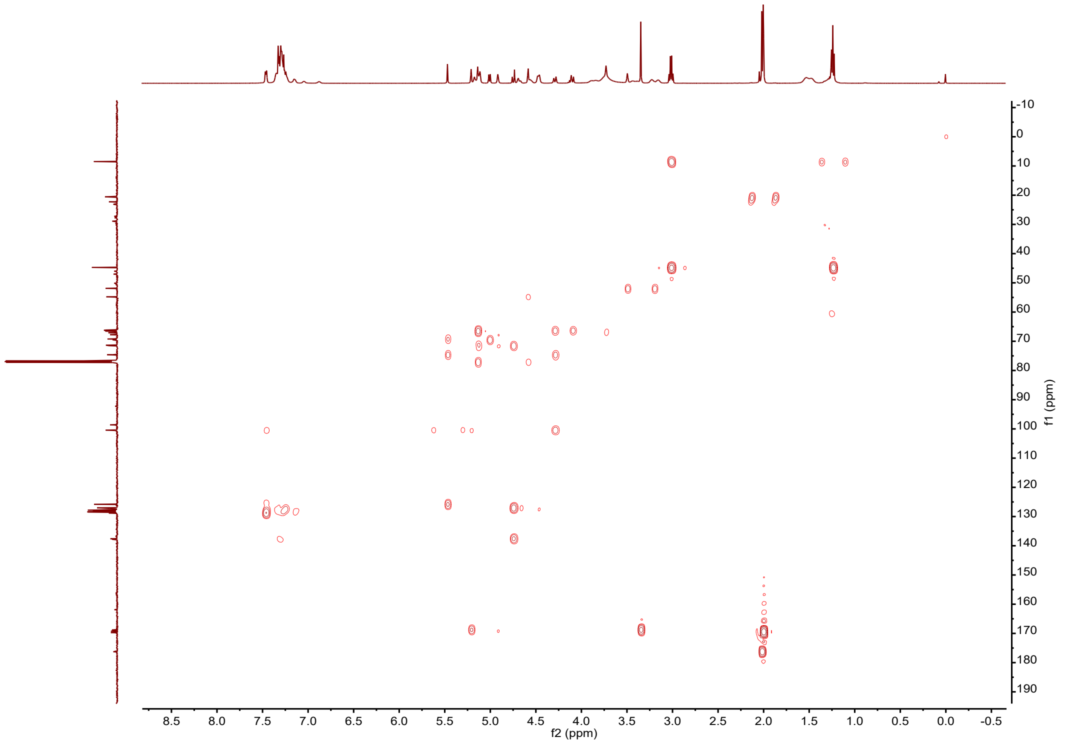

^{1}H NMR spectrum ($CDCl_3$, 500 MHz) of compound 17

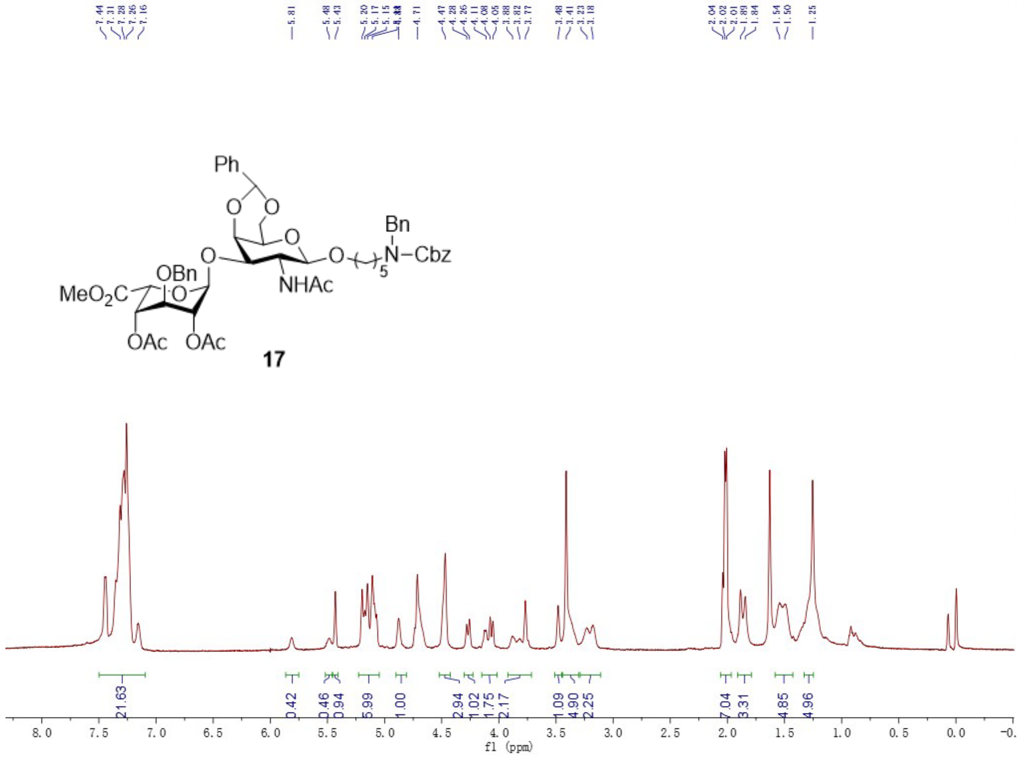

^{13}C NMR spectrum ($CDCl_3$, 126 MHz) of compound 17

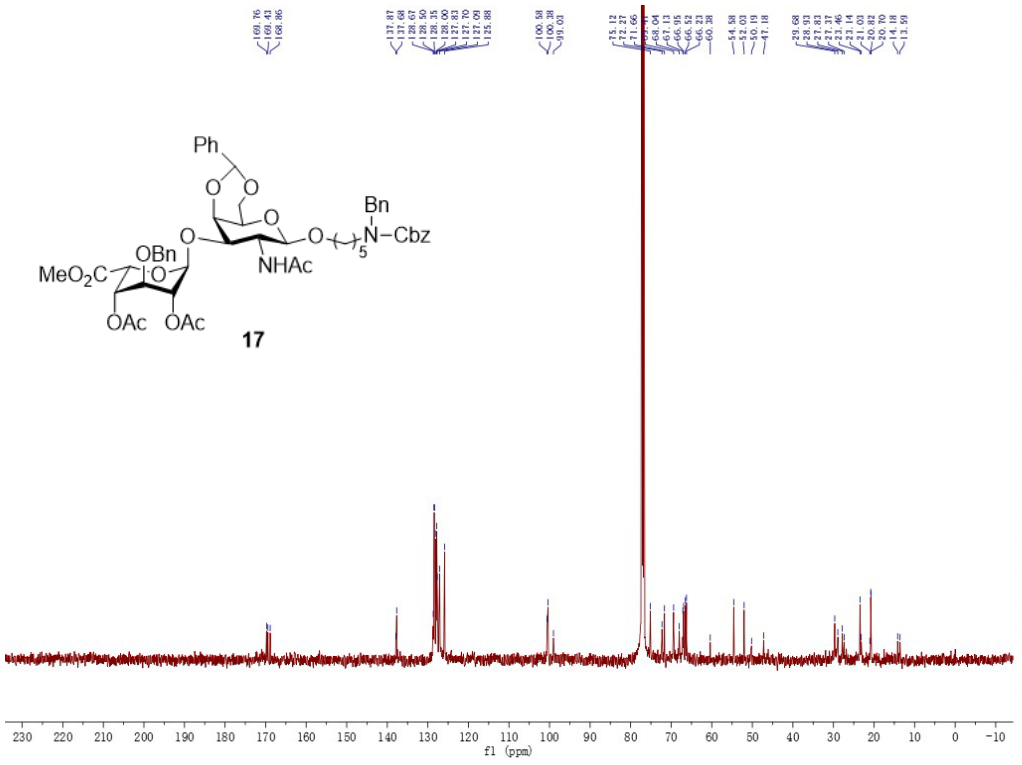

^{1}H-^{1}H COSY NMR spectrum ($CDCl_3$, 500 MHz) of compound 17

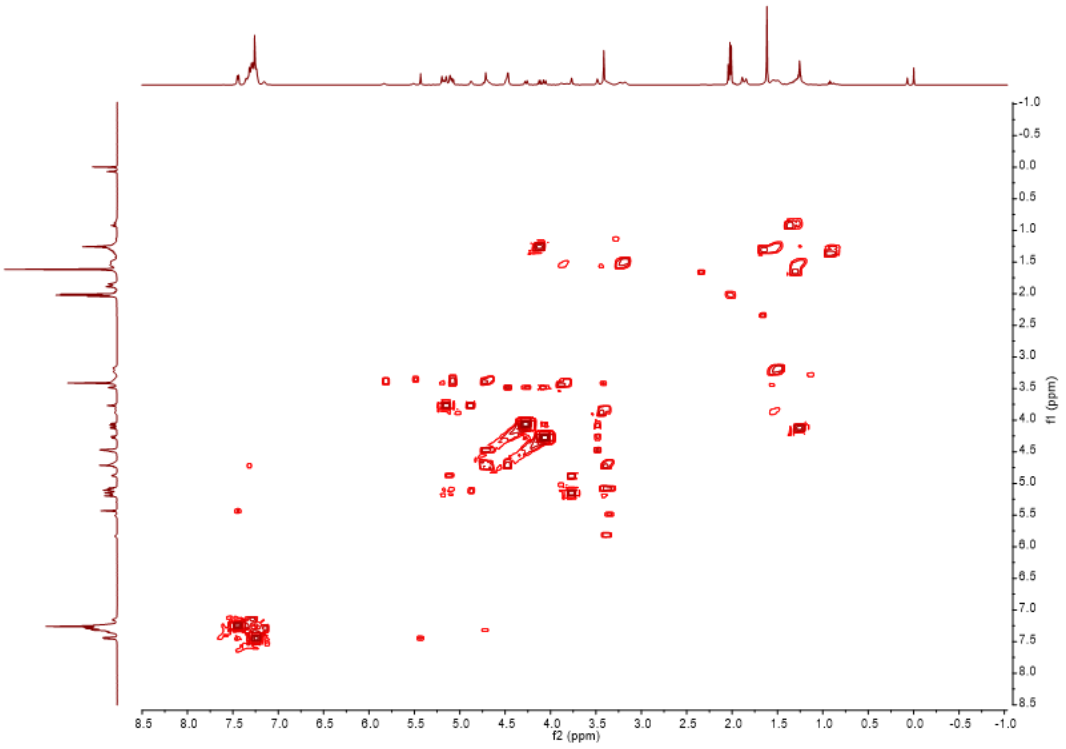

^{1}H-^{13}C HSQC NMR spectrum ($CDCl_3$, 500 MHz) of compound 17

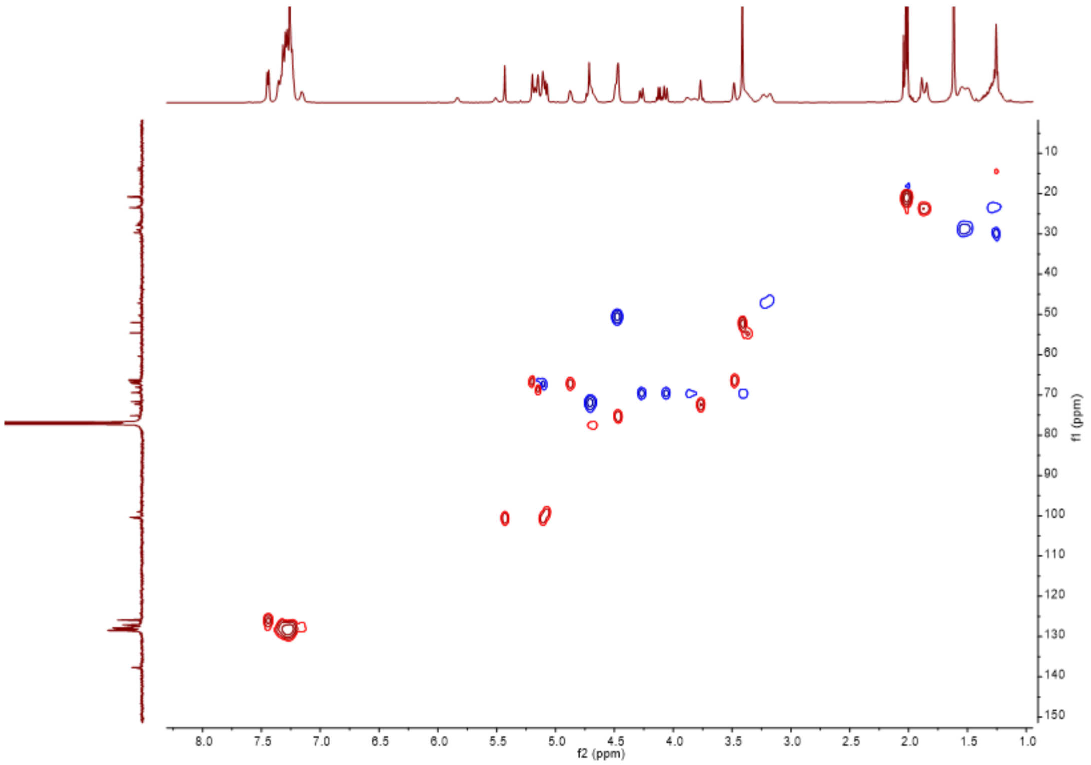

^{1}H-^{13}C HMBC NMR spectrum ($CDCl_3$, 500 MHz) of compound 17

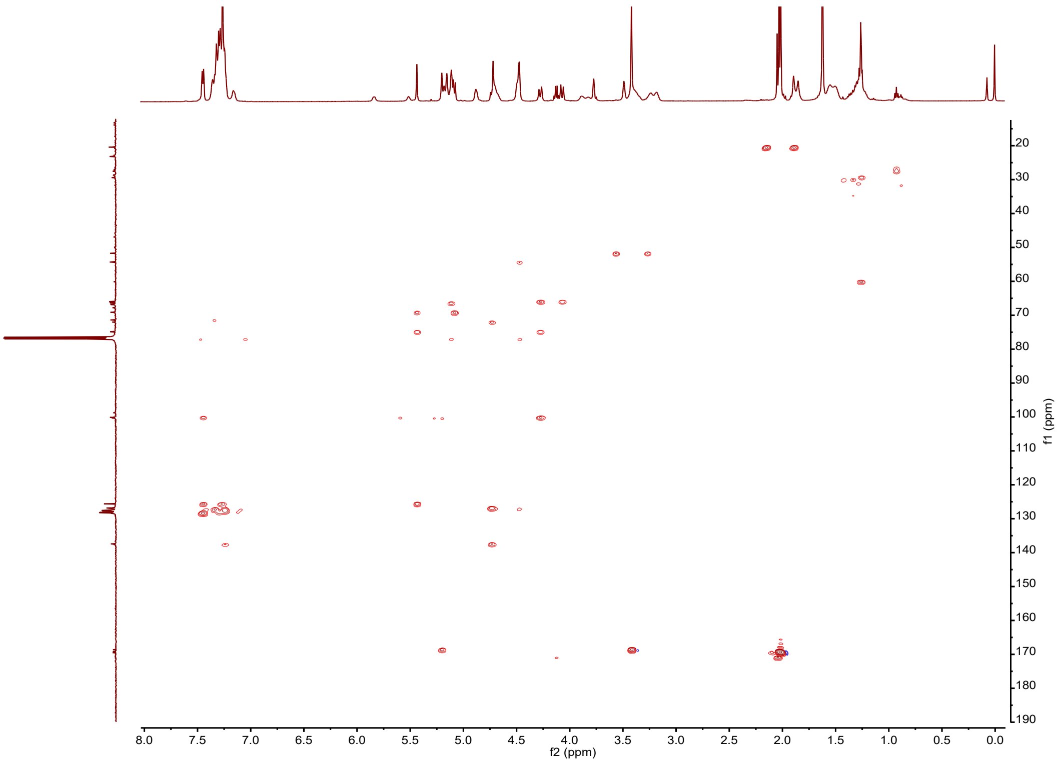

^{1}H NMR spectrum ($CDCl_3$, 500 MHz) of compound 18

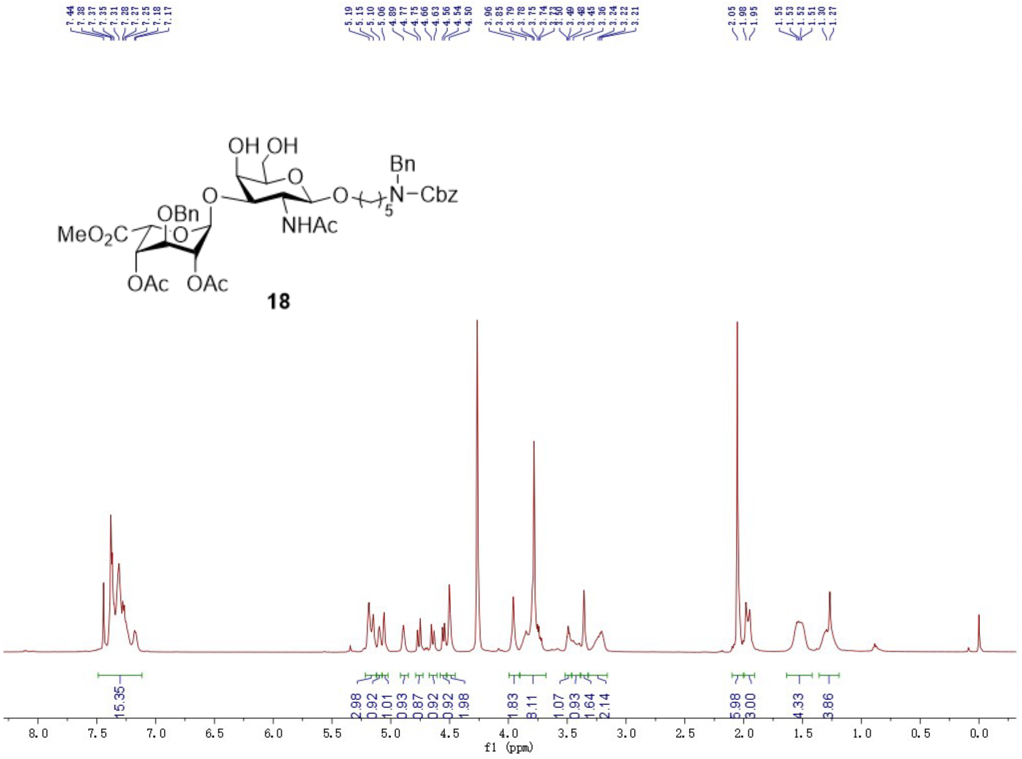

^{13}C NMR spectrum ($CDCl_3$, 126 MHz) of compound 18

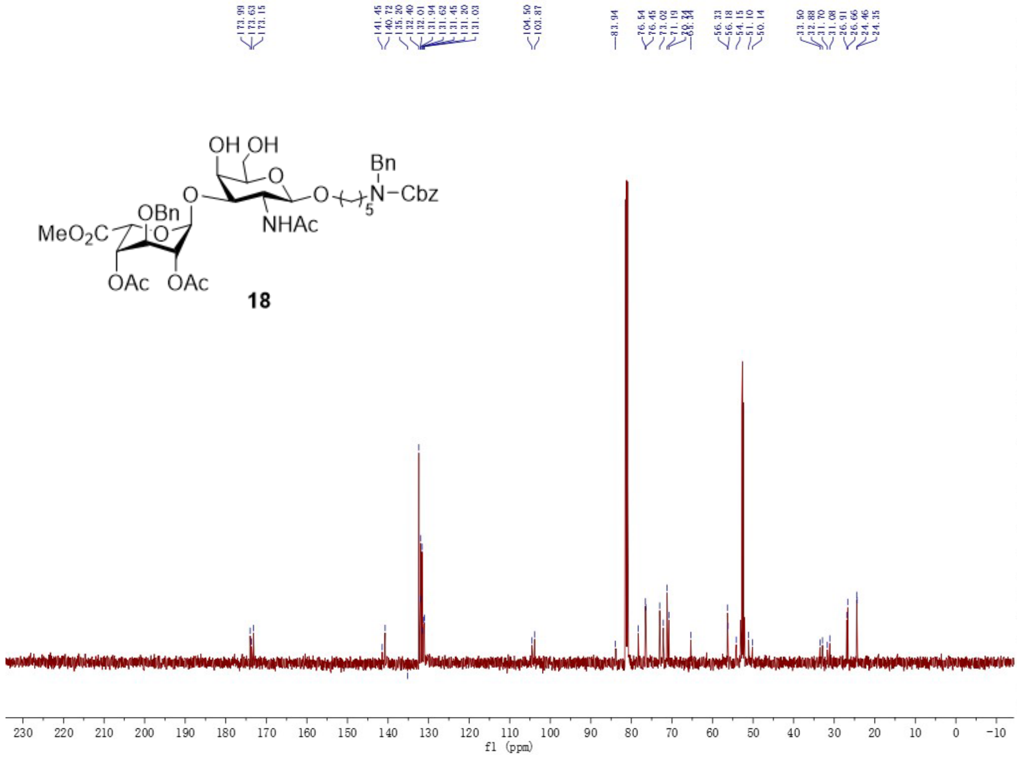

^{1}H-^{1}H COSY NMR spectrum ($CDCl_3$, 500 MHz) of compound 18

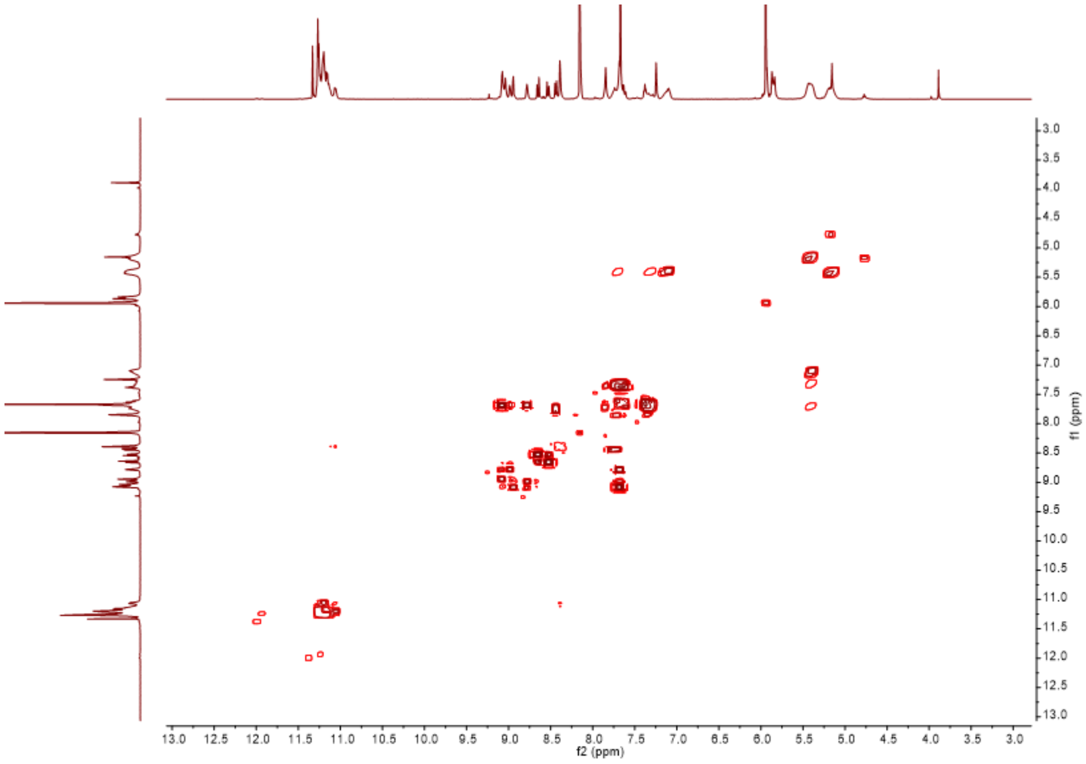

^{1}H-^{13}C HSQC NMR spectrum ($CDCl_3$, 500 MHz) of compound 18

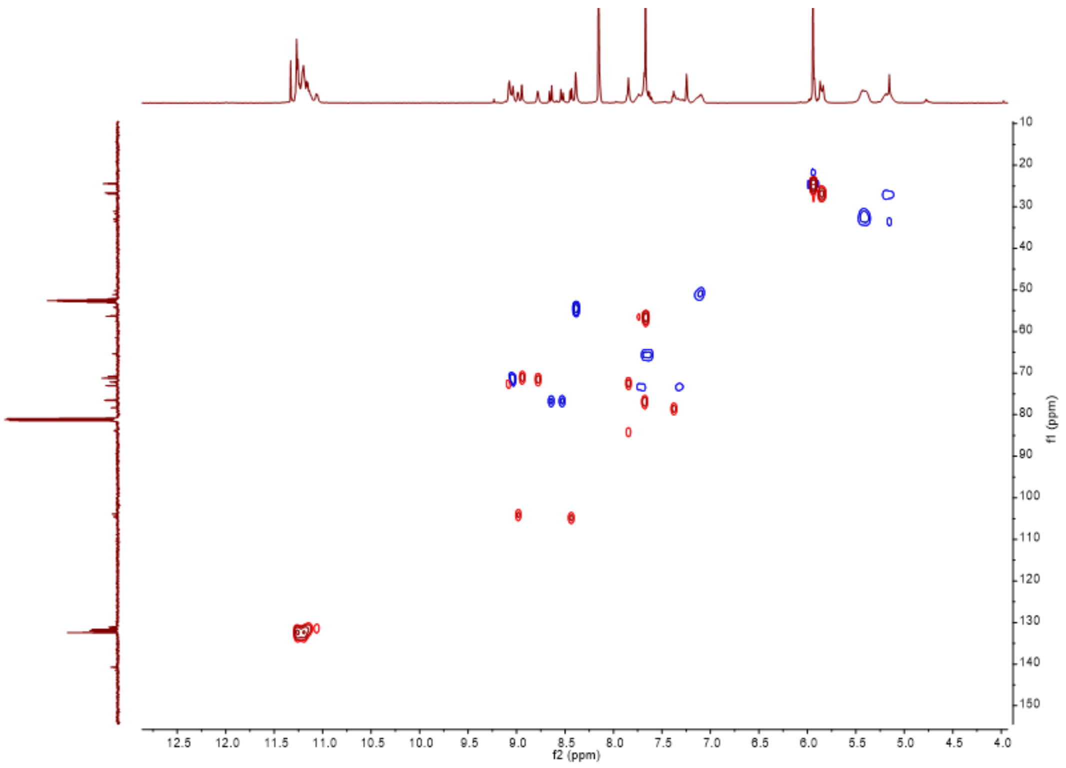

^{1}H-^{13}C HMBC NMR spectrum ($CDCl_3$, 500 MHz) of compound 18

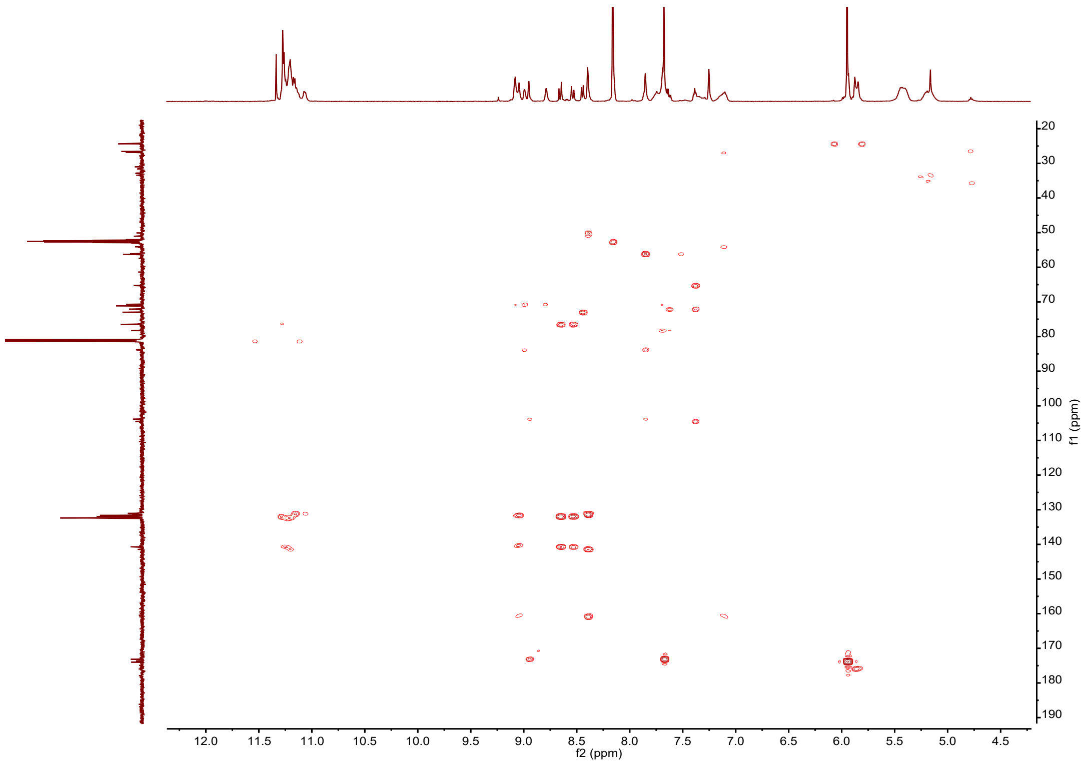

^{1}H NMR spectrum (CD_3OD, 500 MHz) of compound 19

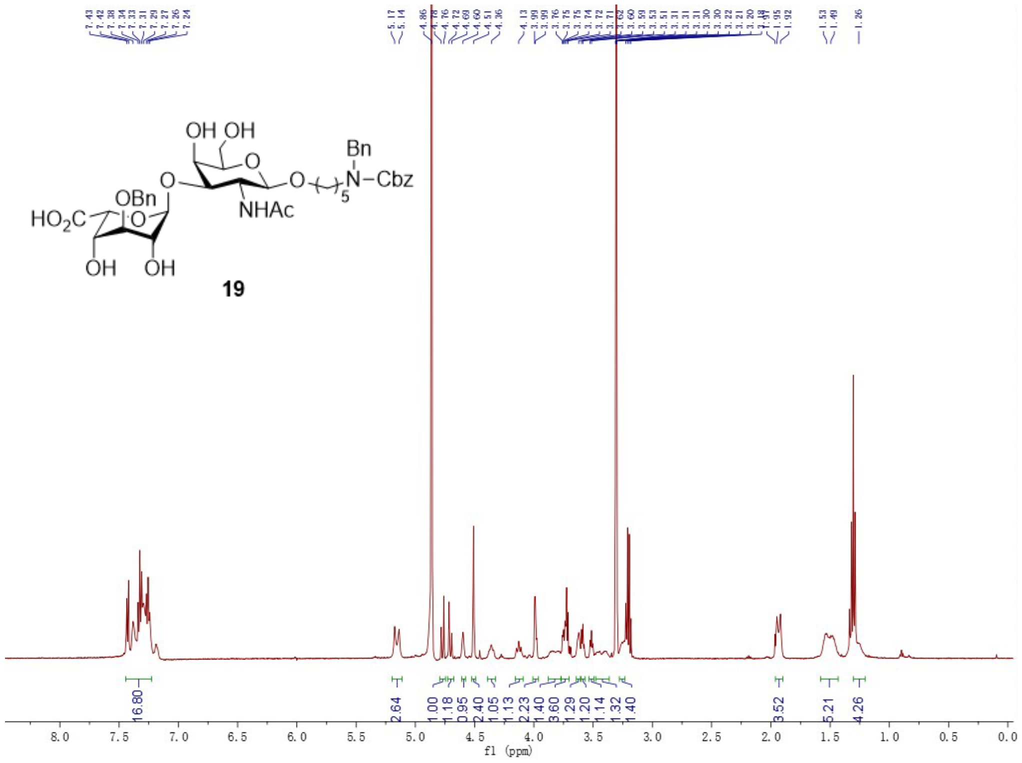

^{13}C NMR spectrum (CD_3OD, 126 MHz) of compound 19

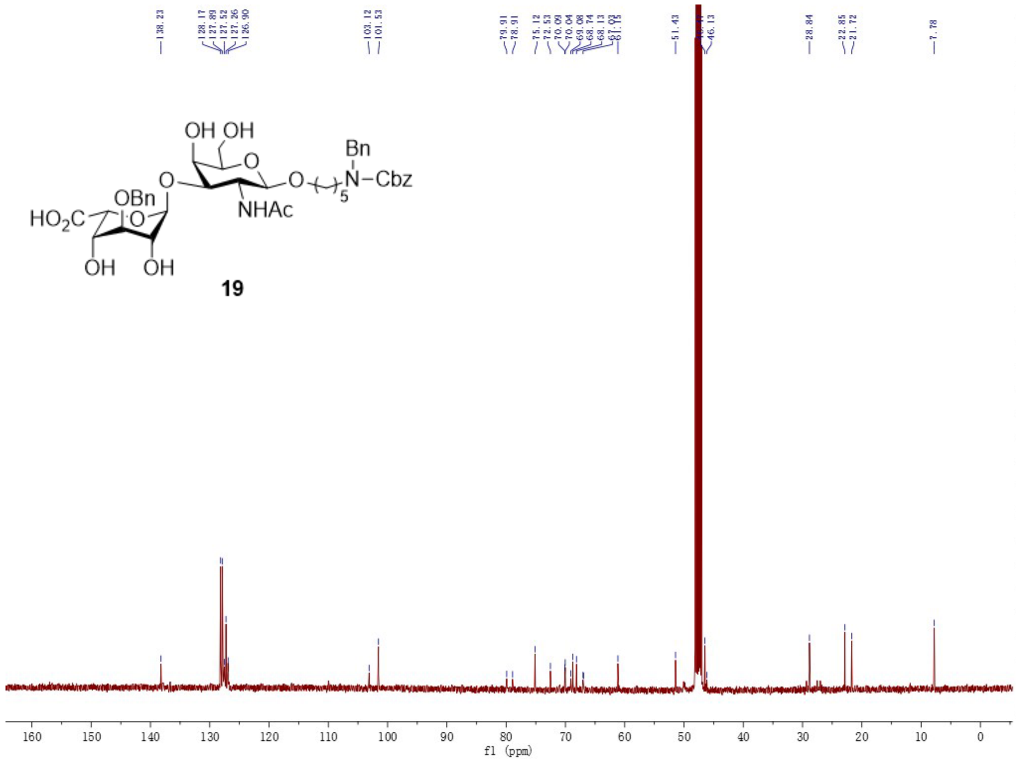

^{1}H-^{1}H COSY NMR spectrum (CD_3OD, 500 MHz) of compound 19

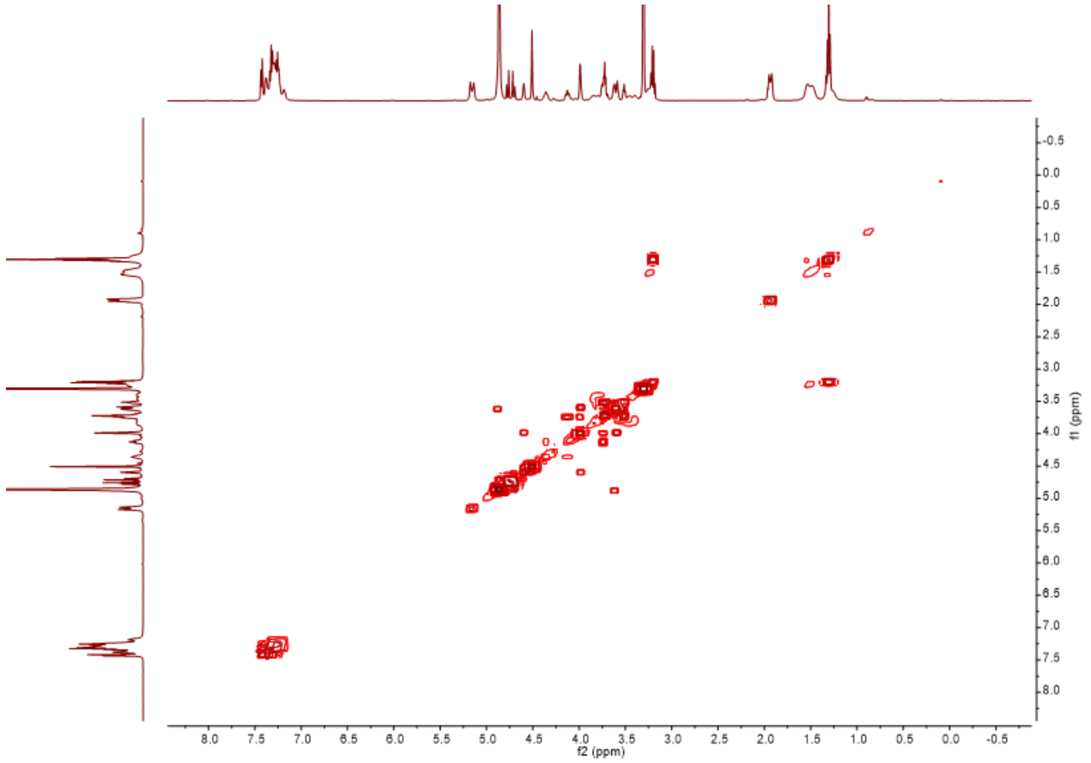

^{1}H-^{13}C HSQC NMR spectrum (CD_3OD, 500 MHz) of compound 19

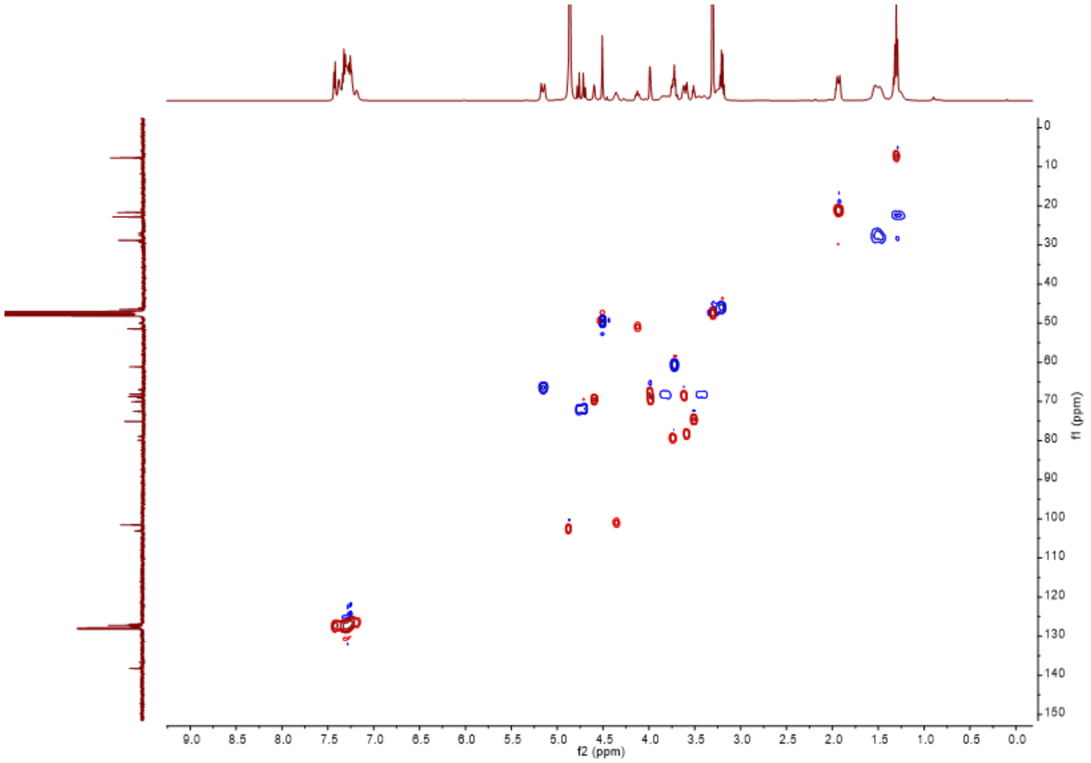

^{1}H NMR spectrum (D_2O, 500 MHz) of compound 20 (DM1)

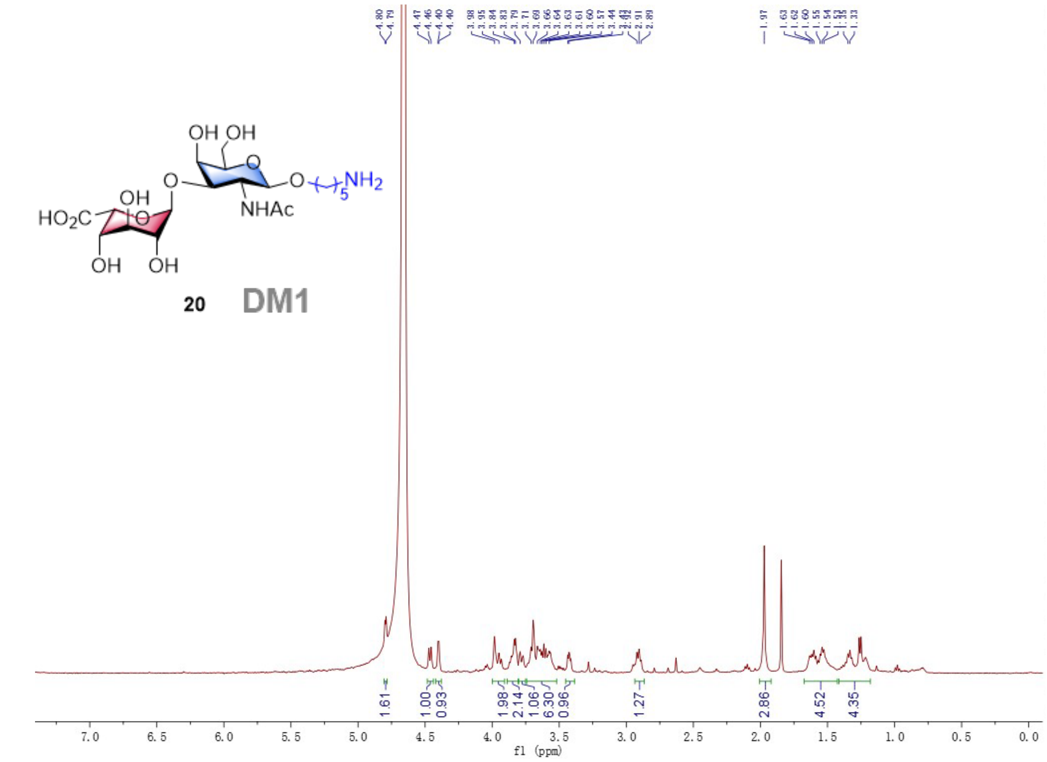

^{1}H-^{1}H COSY NMR spectrum (D_2O, 500 MHz) of compound 20 (DM1)

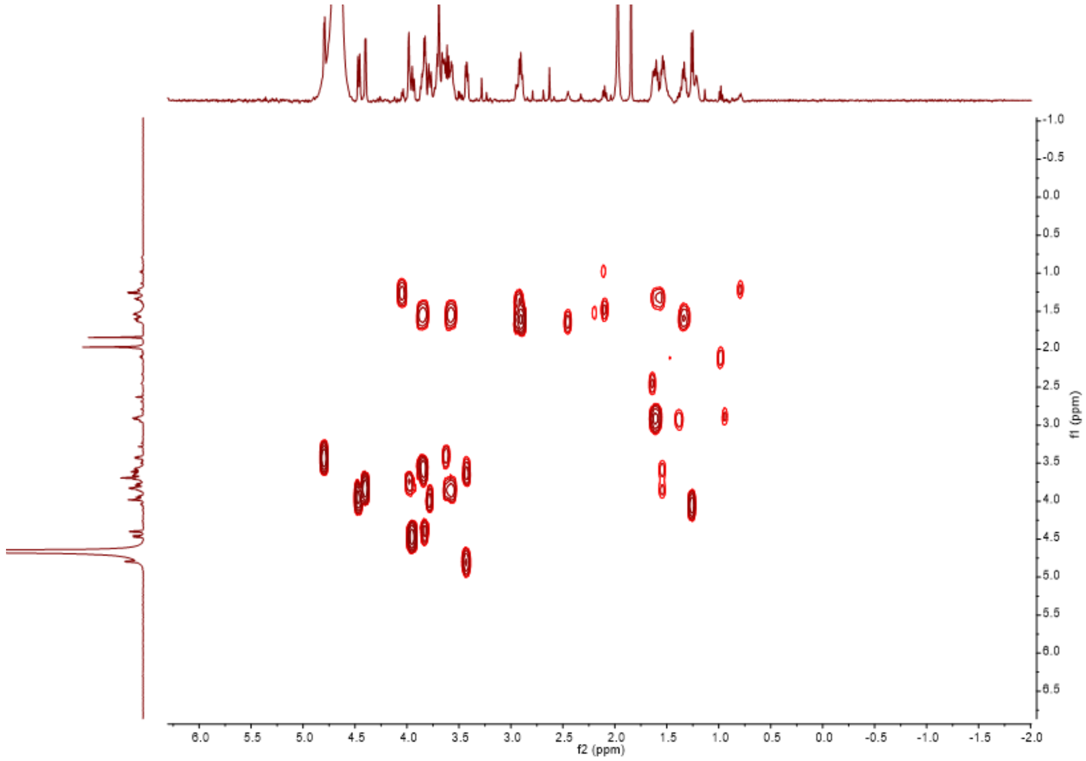

^{1}H NMR spectrum (CD_3OD, 500 MHz) of compound 21

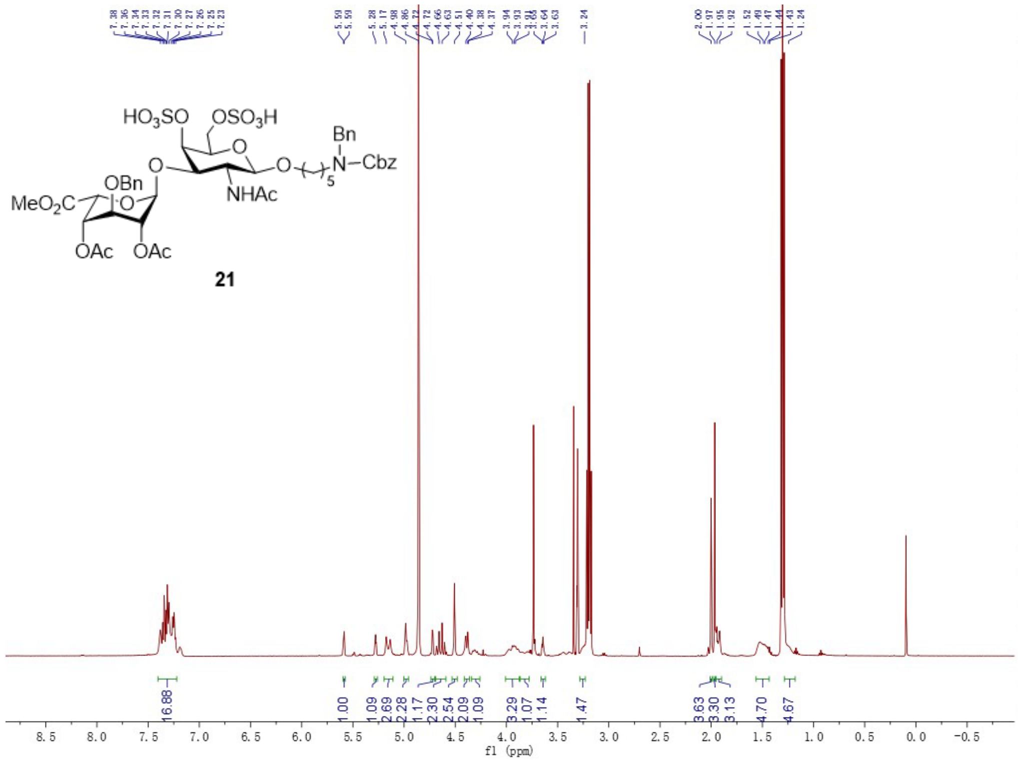

^{13}C NMR spectrum (CD_3OD, 126 MHz) of compound 21

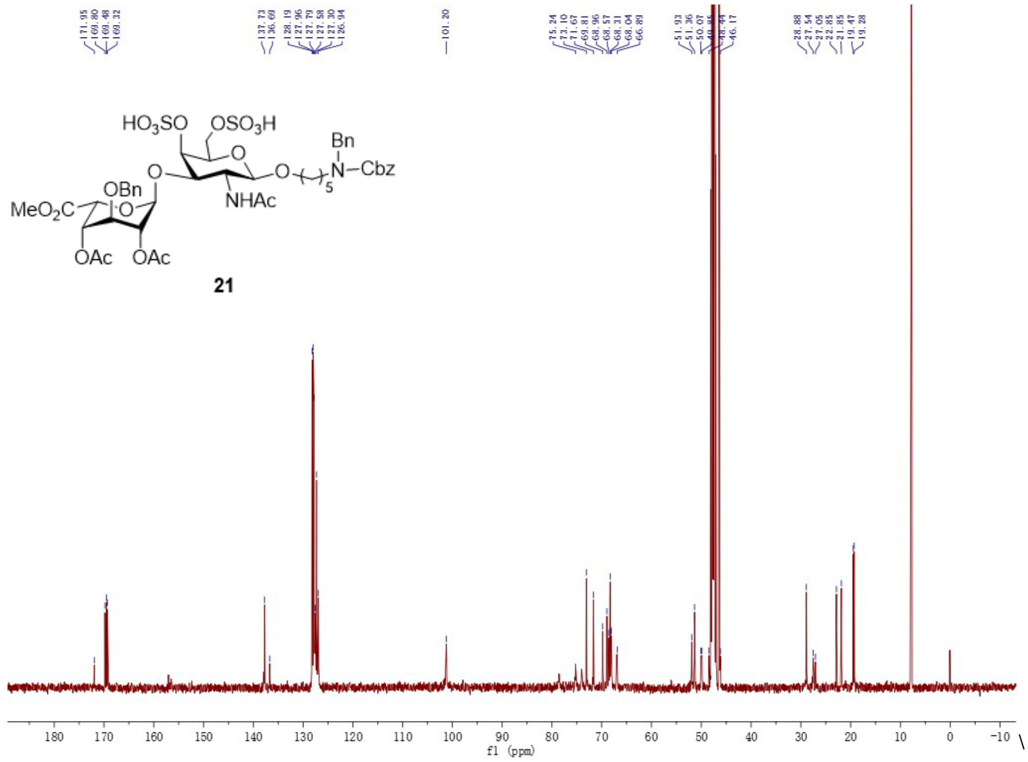

^{1}H-^{1}H COSY NMR spectrum (CD_3OD, 500 MHz) of compound 21

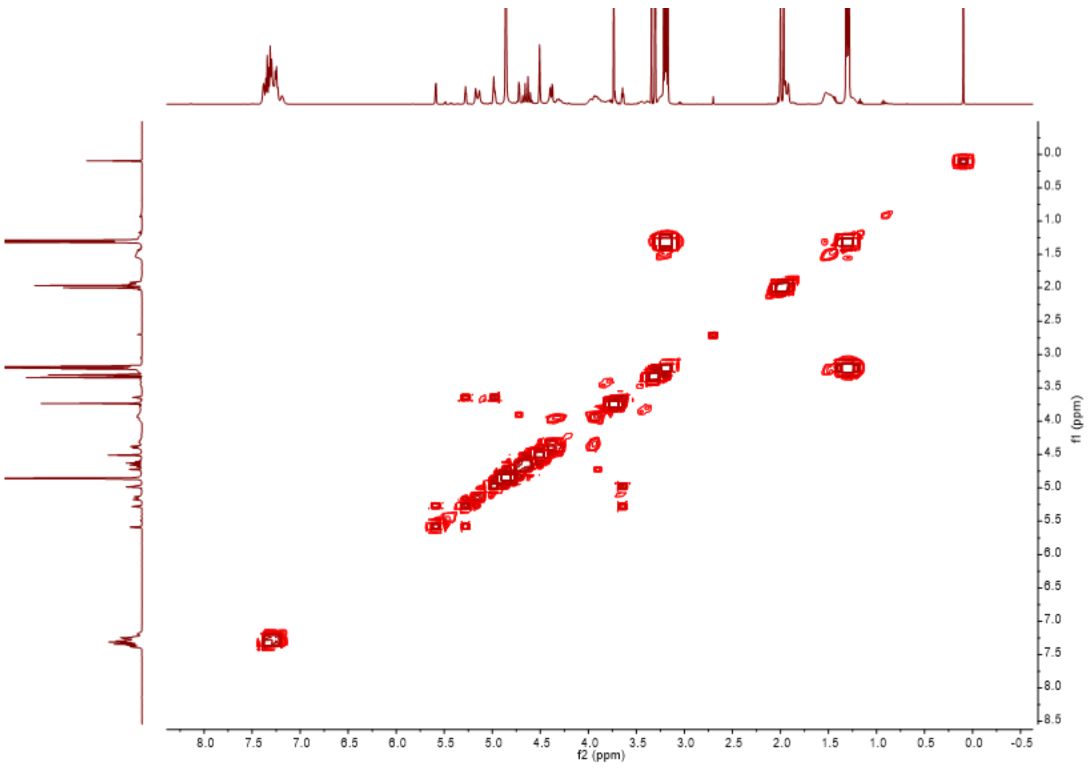

^{1}H-^{13}C HSQC NMR spectrum (CD_3OD, 500 MHz) of compound 21

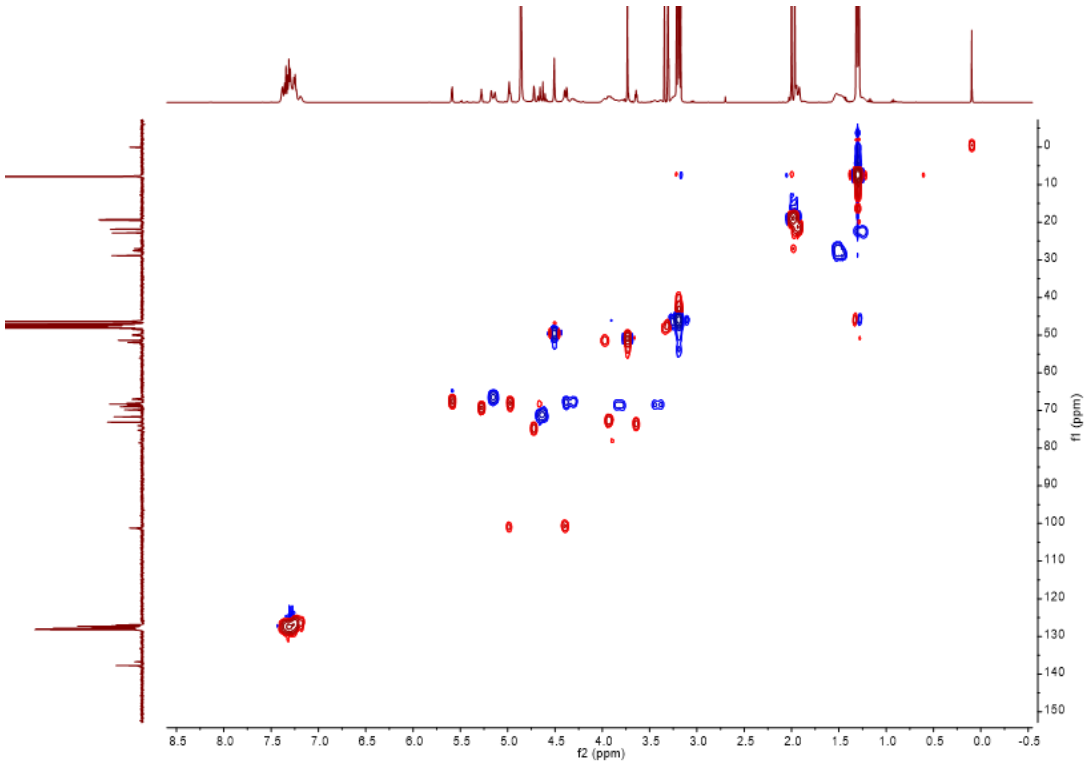

^{1}H NMR spectrum (CD_3OD, 500 MHz) of compound 22

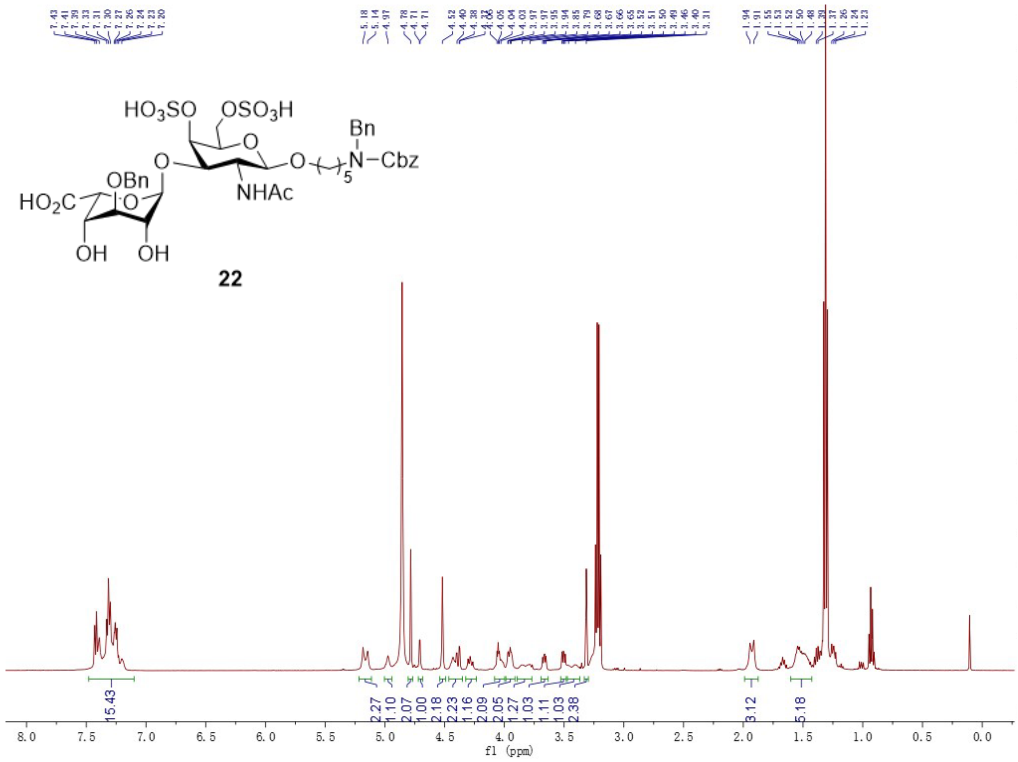

^{13}C NMR spectrum (CD_3OD, 126 MHz) of compound 22

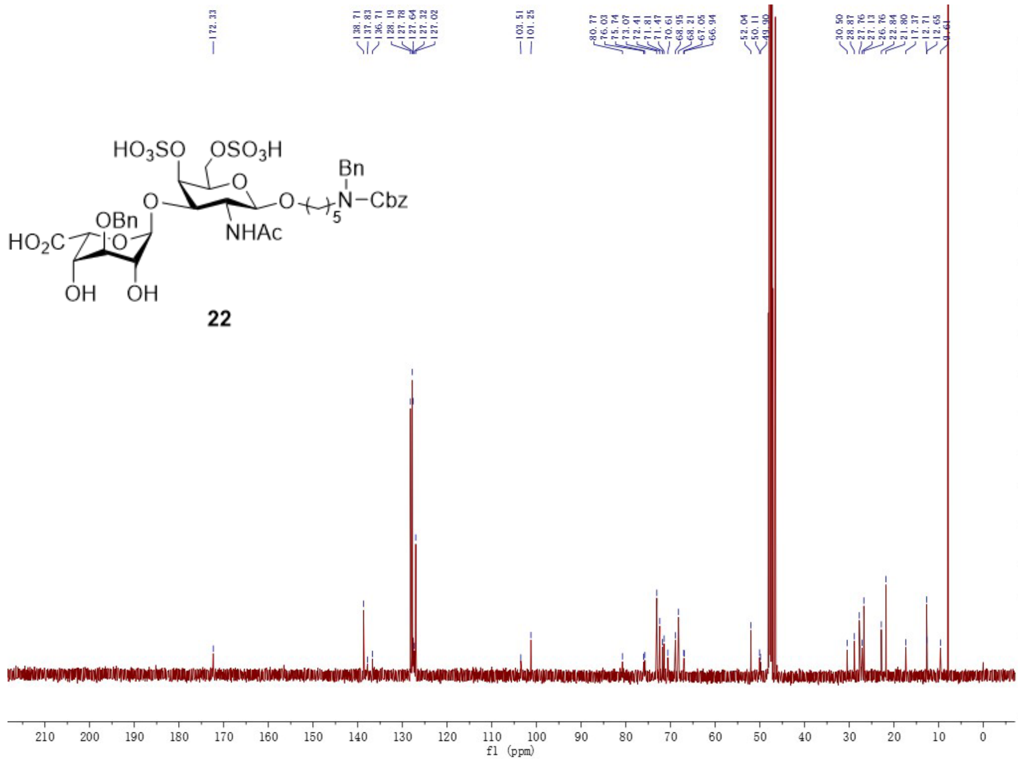

^{1}H-^{1}H COSY NMR spectrum (CD_3OD, 500 MHz) of compound 22

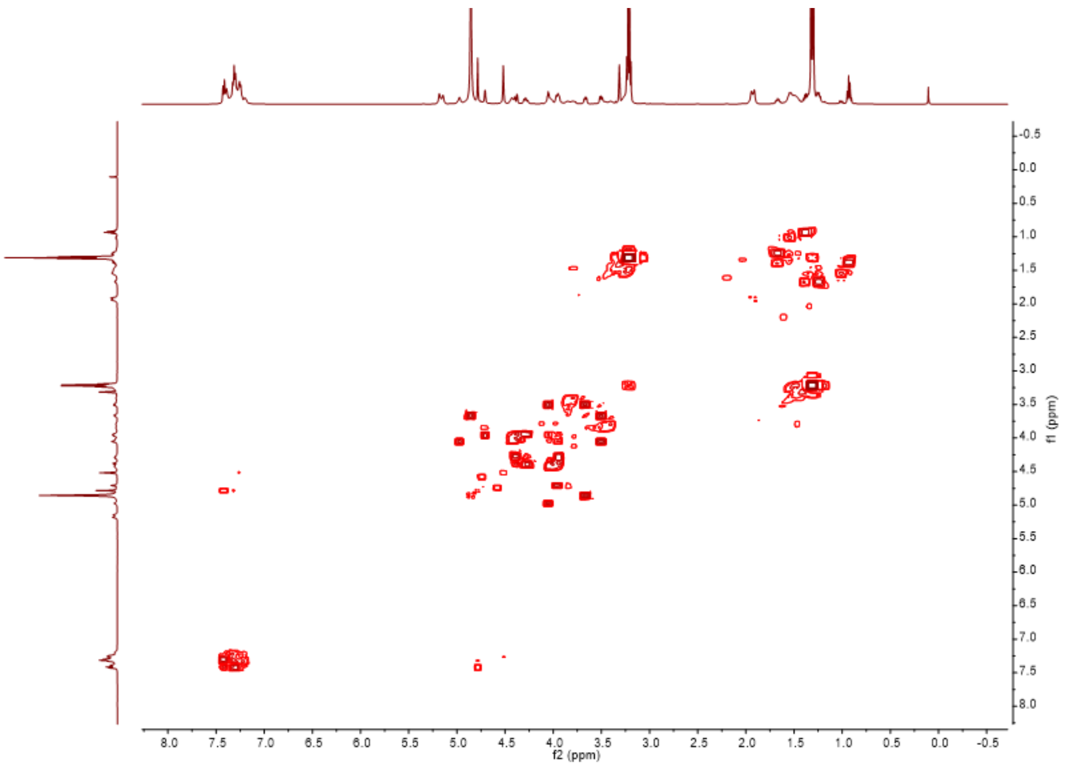

^{1}H-^{13}C HSQC NMR spectrum (CD_3OD, 500 MHz) of compound 22

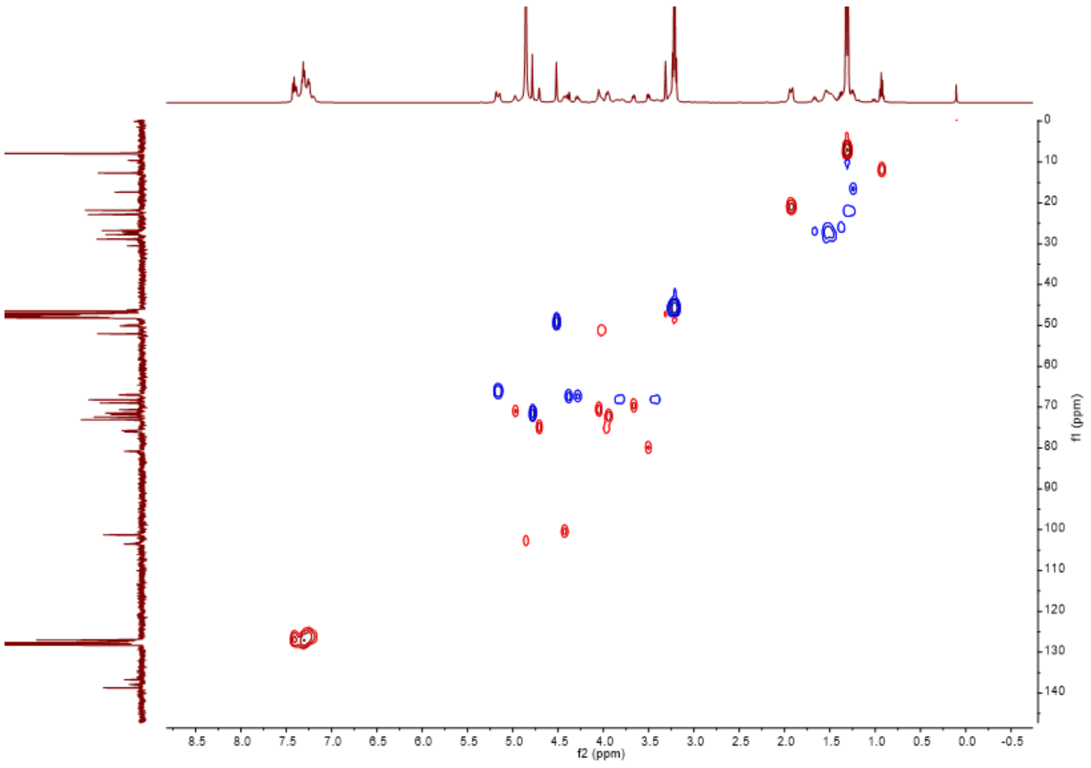

^{1}H NMR spectrum (D_2O, 500 MHz) of compound 23 (DM2)

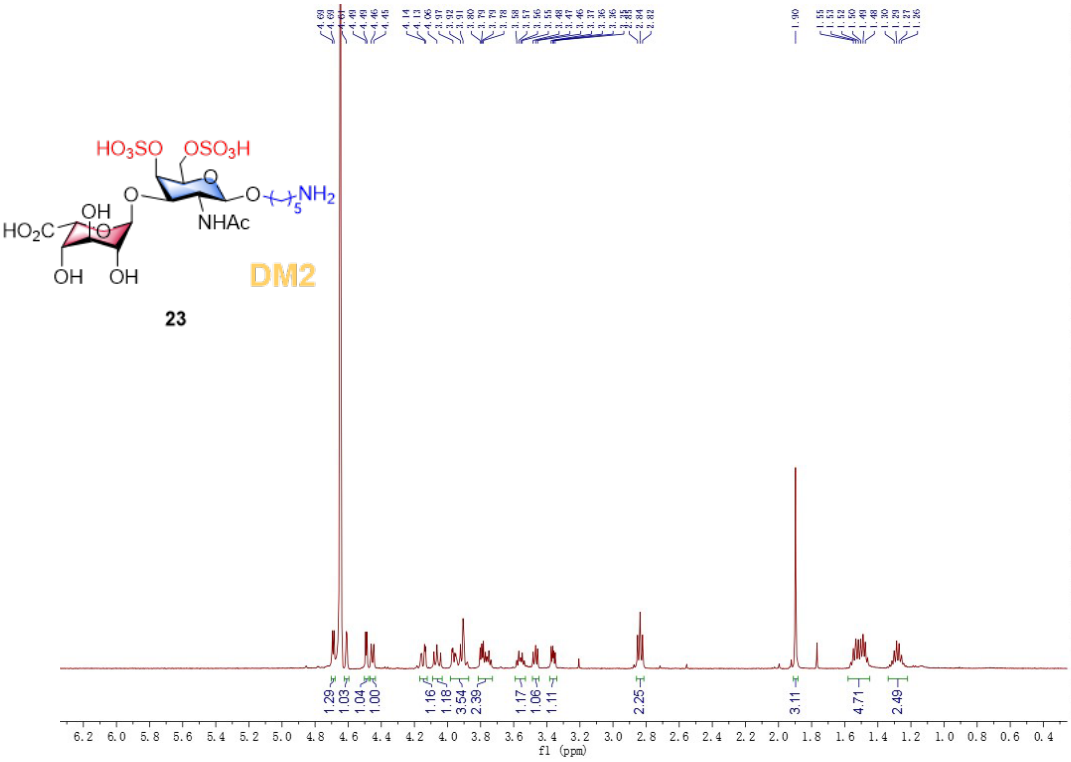

^{13}C NMR spectrum (D_2O, 126 MHz) of compound 23 (DM2)

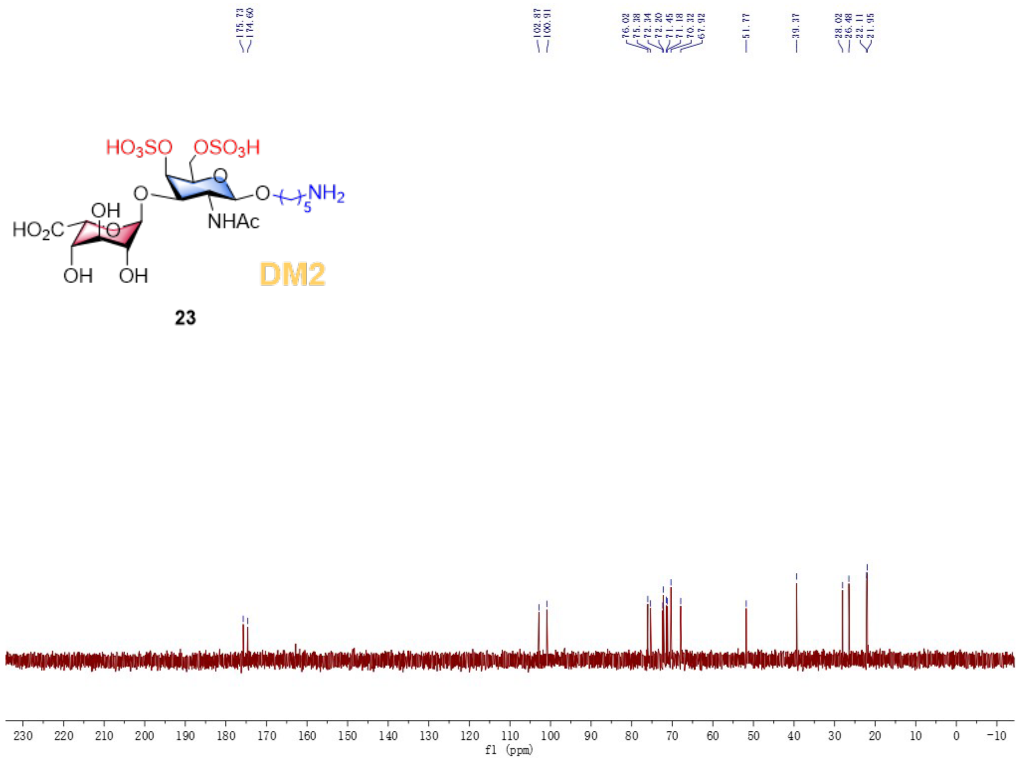

^{1}H-^{1}H COSY NMR spectrum (D_2O, 500 MHz) of compound 23 (DM2)

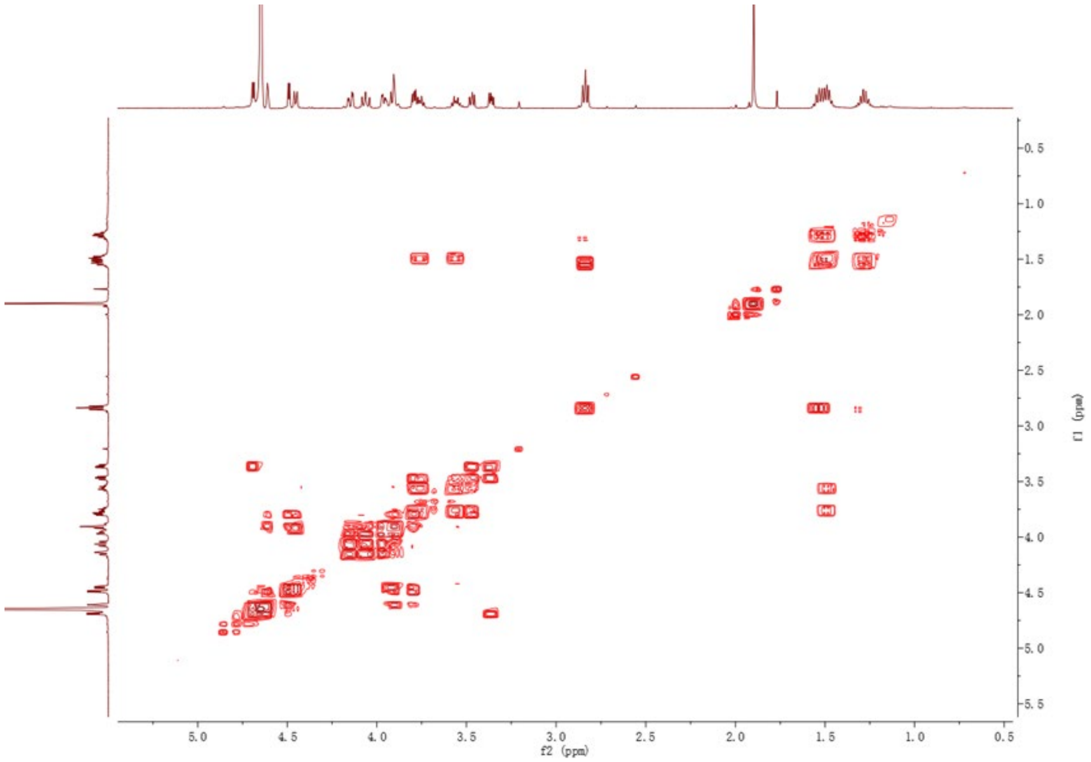

^{1}H-^{13}C HSQC NMR spectrum (D_2O, 500 MHz) of compound 23 (DM2)

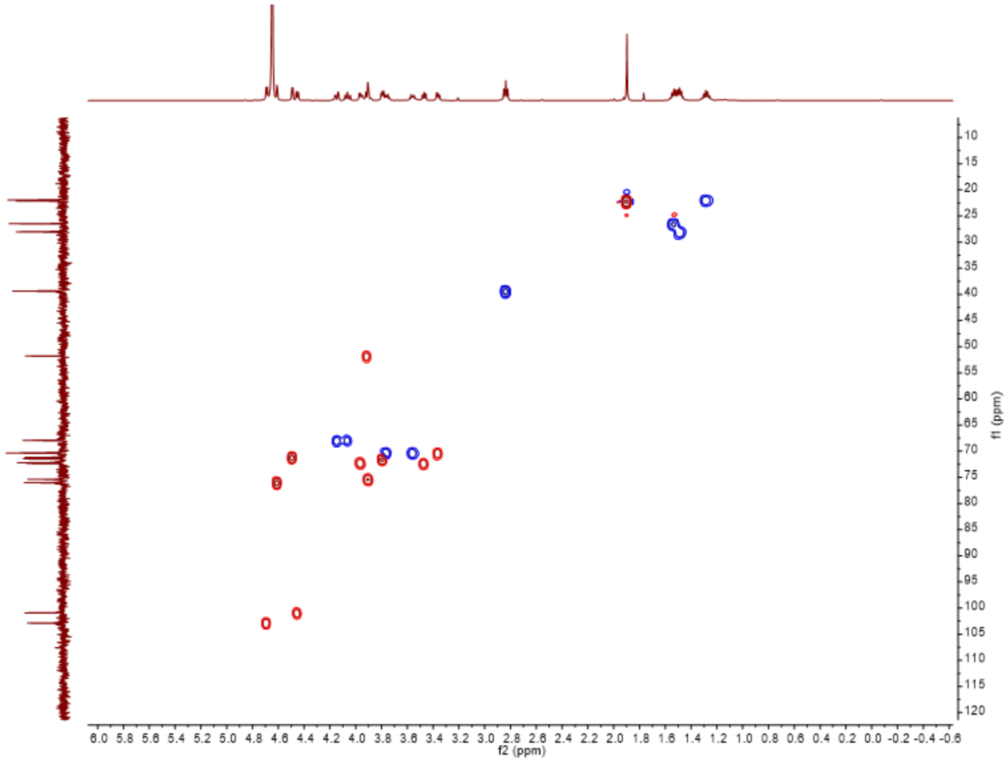

^{1}H-^{13}C HMBC NMR spectrum (D_2O, 500 MHz) of compound 23 (DM2)

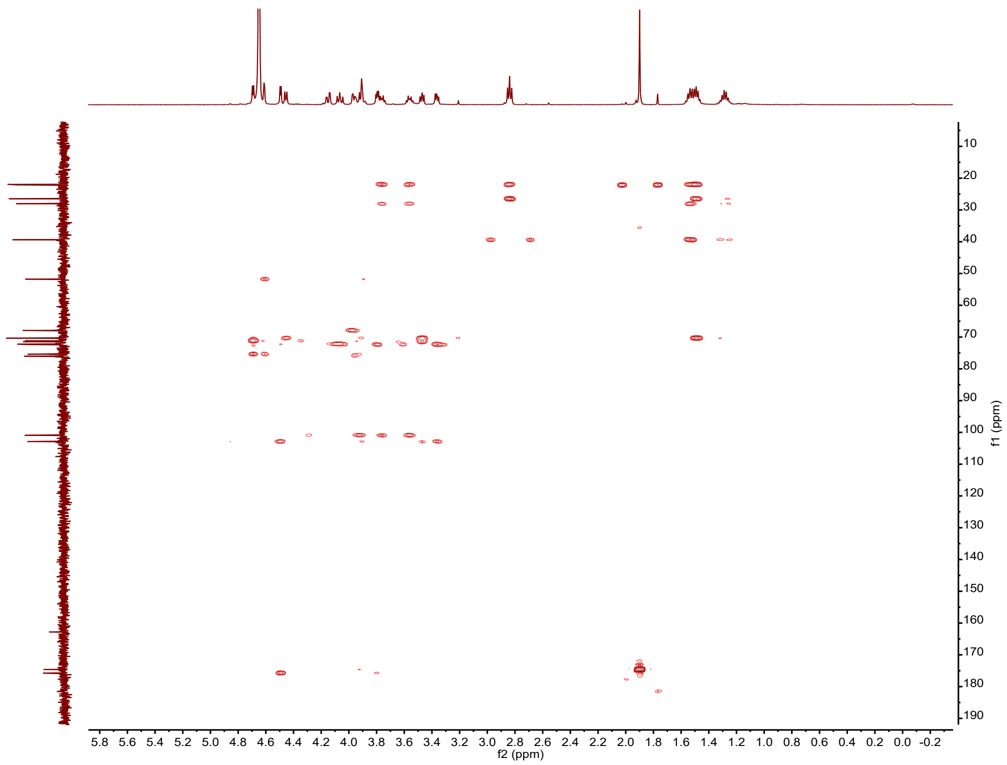

^{1}H NMR spectrum (CD_3OD, 500 MHz) of compound 24

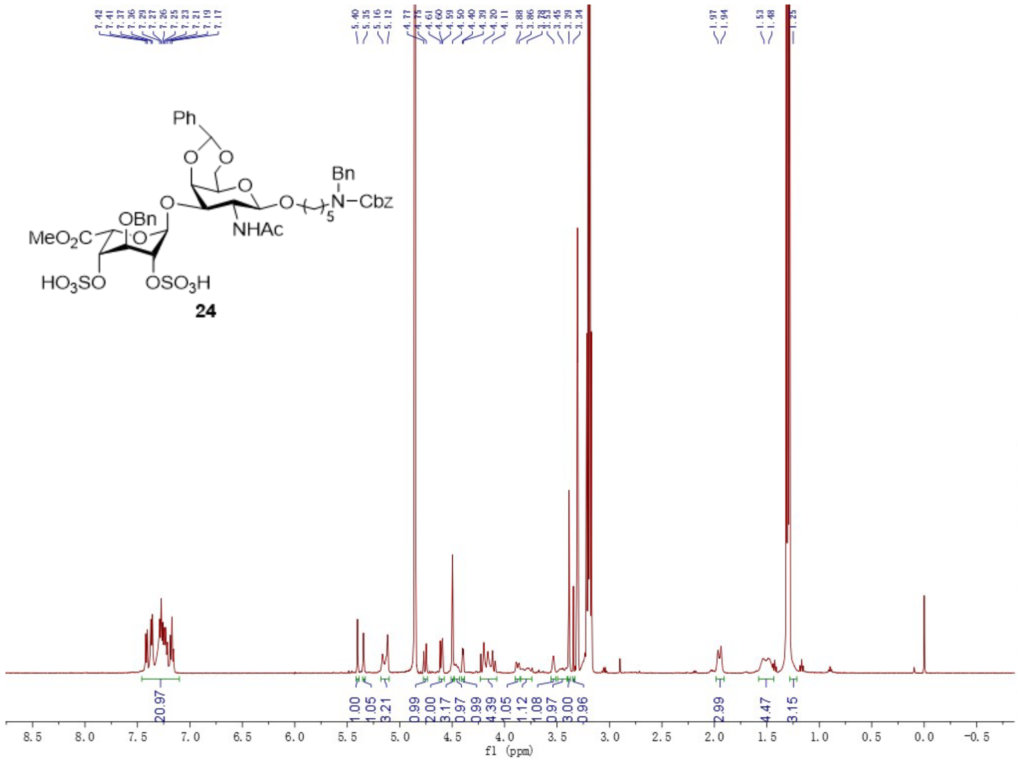

^{13}C NMR spectrum (CD_3OD, 126 MHz) of compound 24

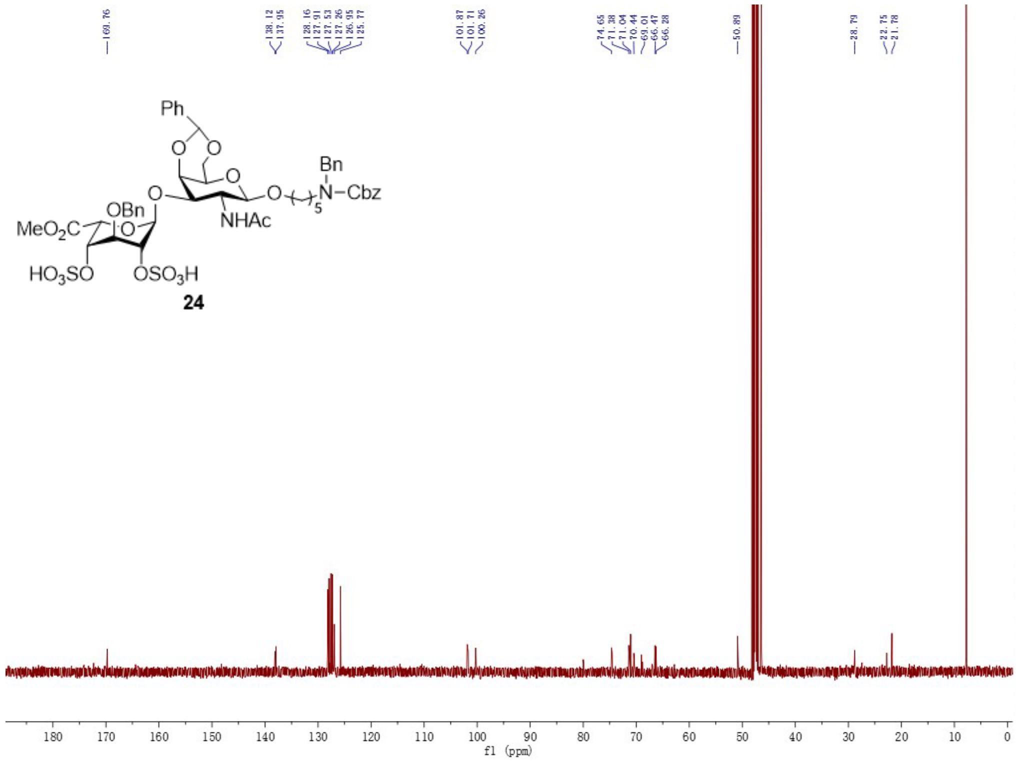

^1H-^1H COSY NMR spectrum (CD_3OD, 500 MHz) of compound 24

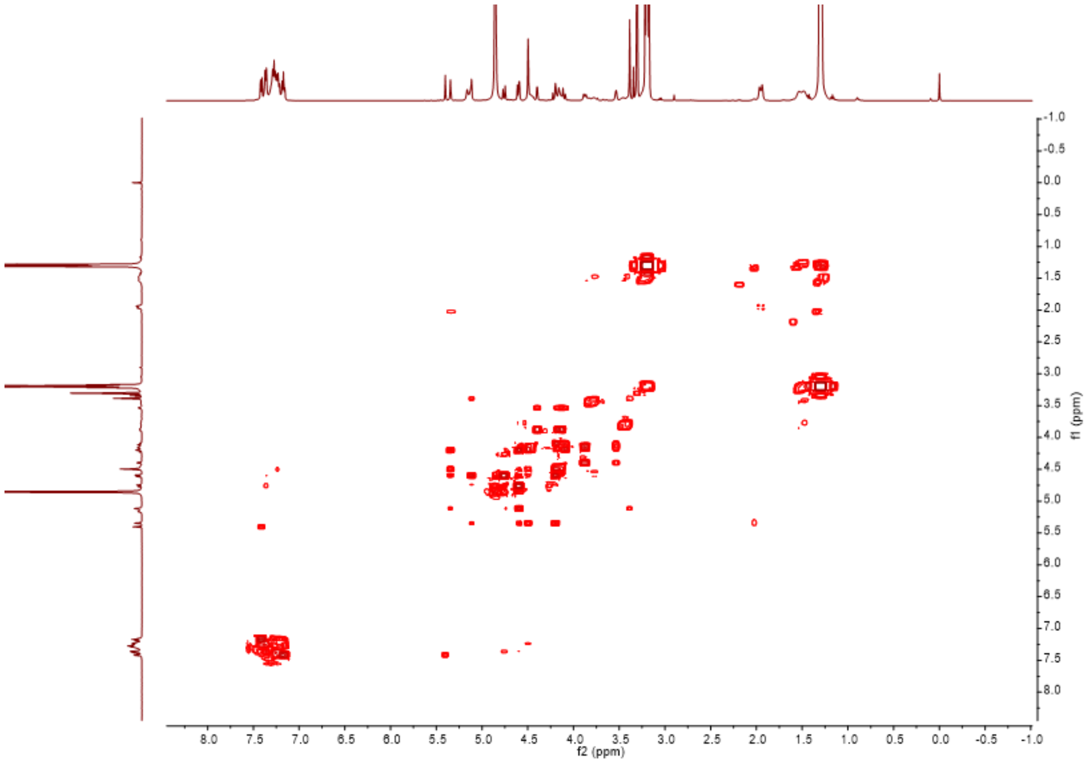

^{1}H-^{13}C HSQC NMR spectrum (CD_3OD, 500 MHz) of compound 24

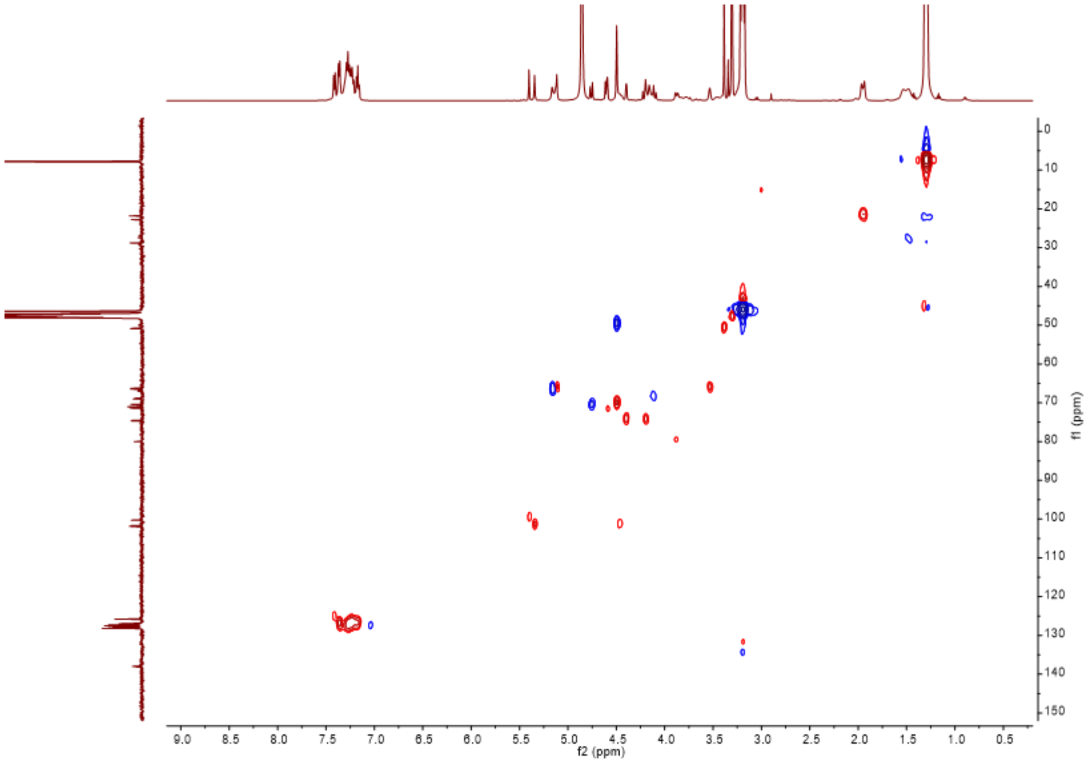

^{1}H-^{13}C HMBC NMR spectrum (CD_3OD, 500 MHz) of compound 24

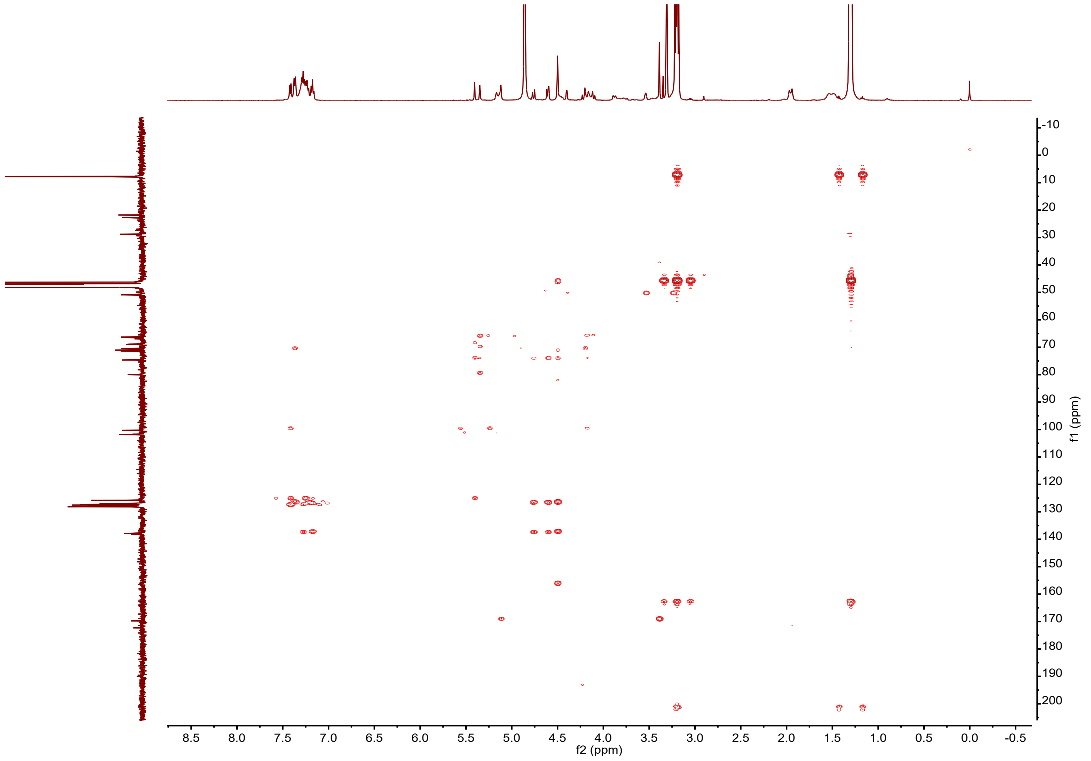

^{1}H NMR spectrum (CD_3OD, 500 MHz) of compound 25

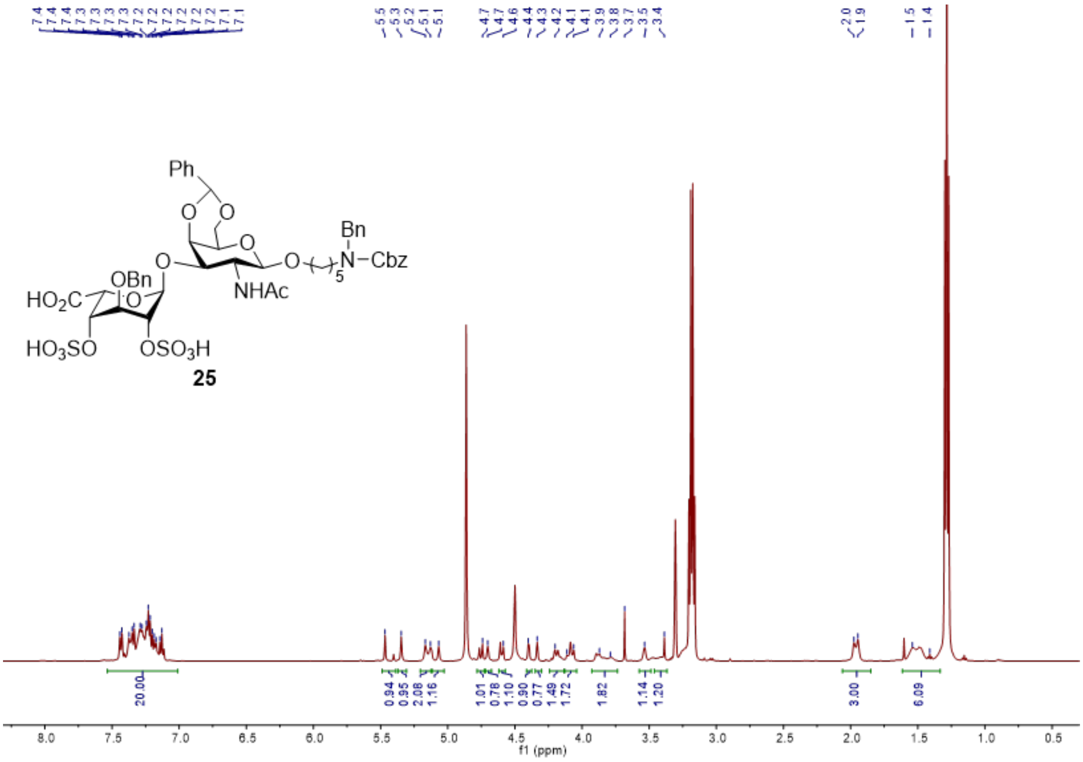

^{13}C NMR spectrum (CD_3OD, 126 MHz) of compound 25

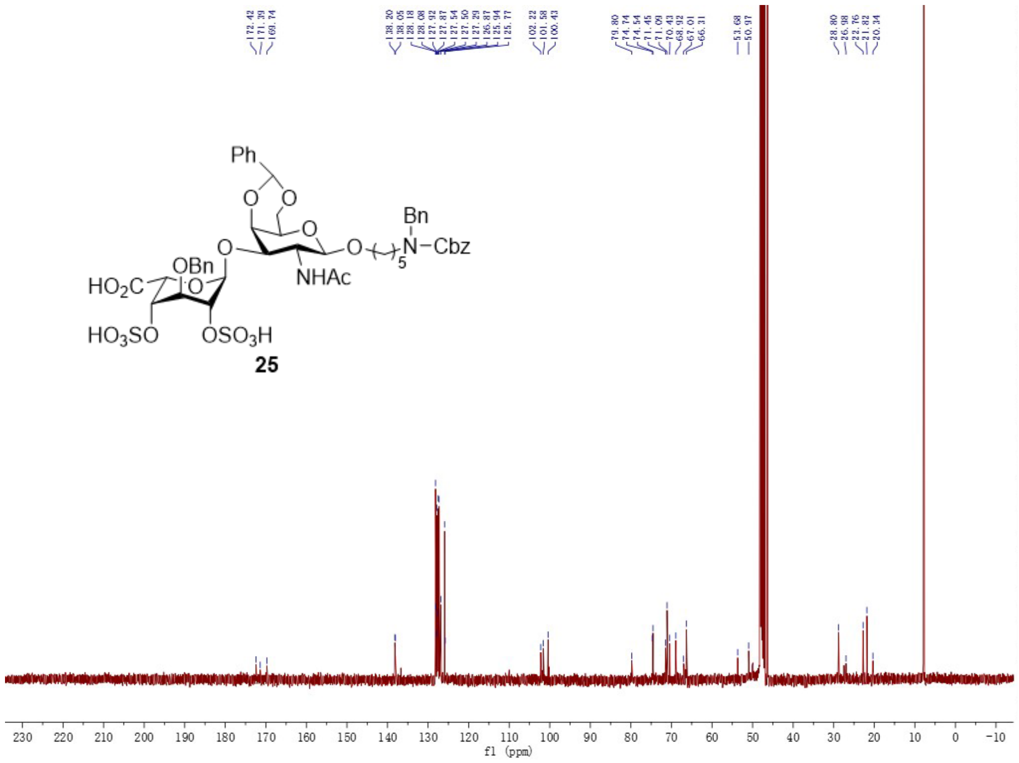

^{1}H-^{1}H COSY NMR spectrum (CD_3OD, 500 MHz) of compound 25

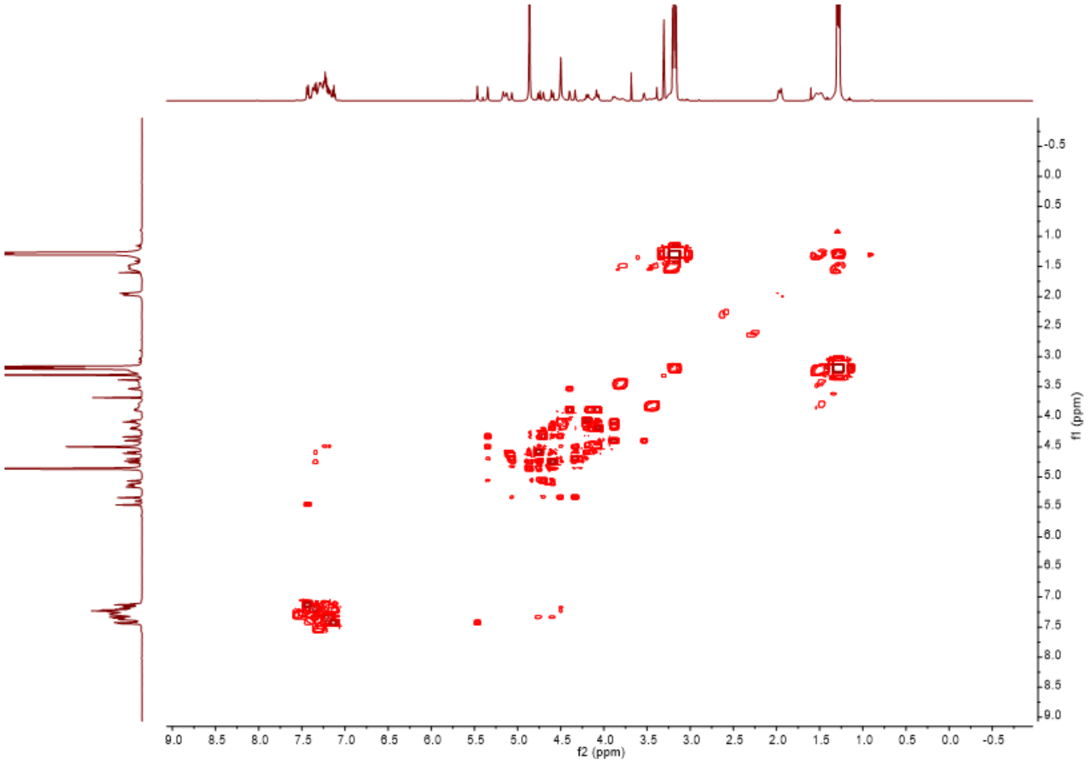

^{1}H-^{13}C HSQC NMR spectrum (CD_3OD, 500 MHz) of compound 25

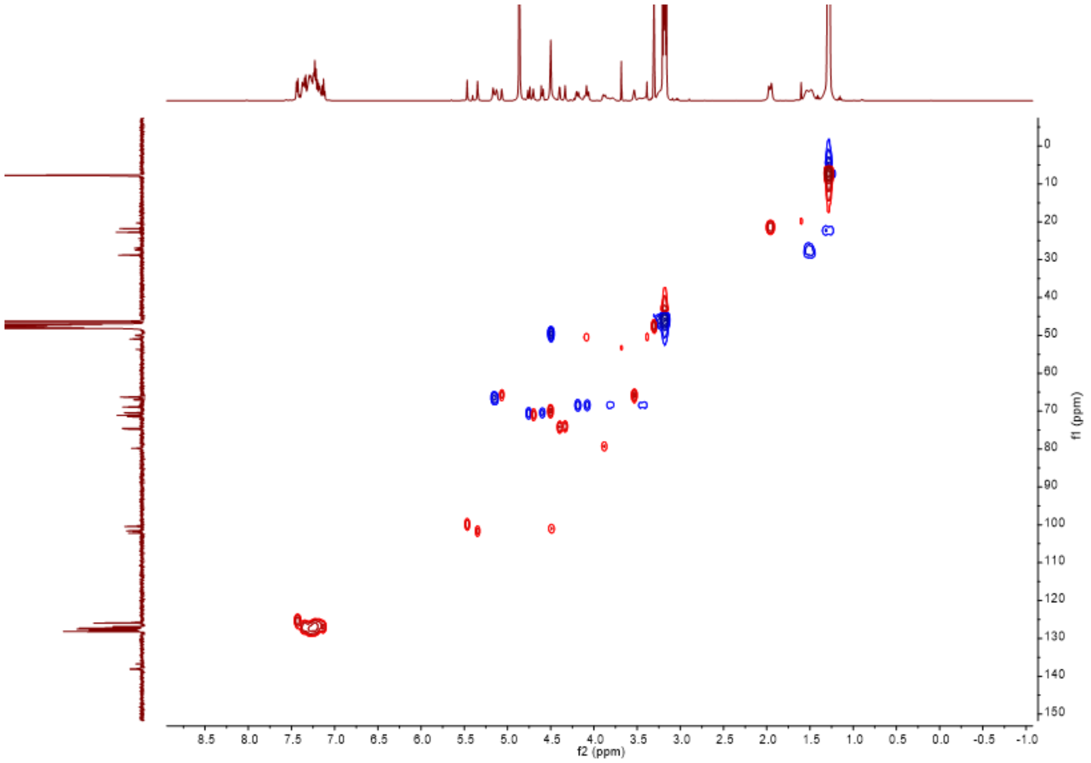

^{1}H NMR spectrum (D_2O, 500 MHz) of compound 26 (DM3)

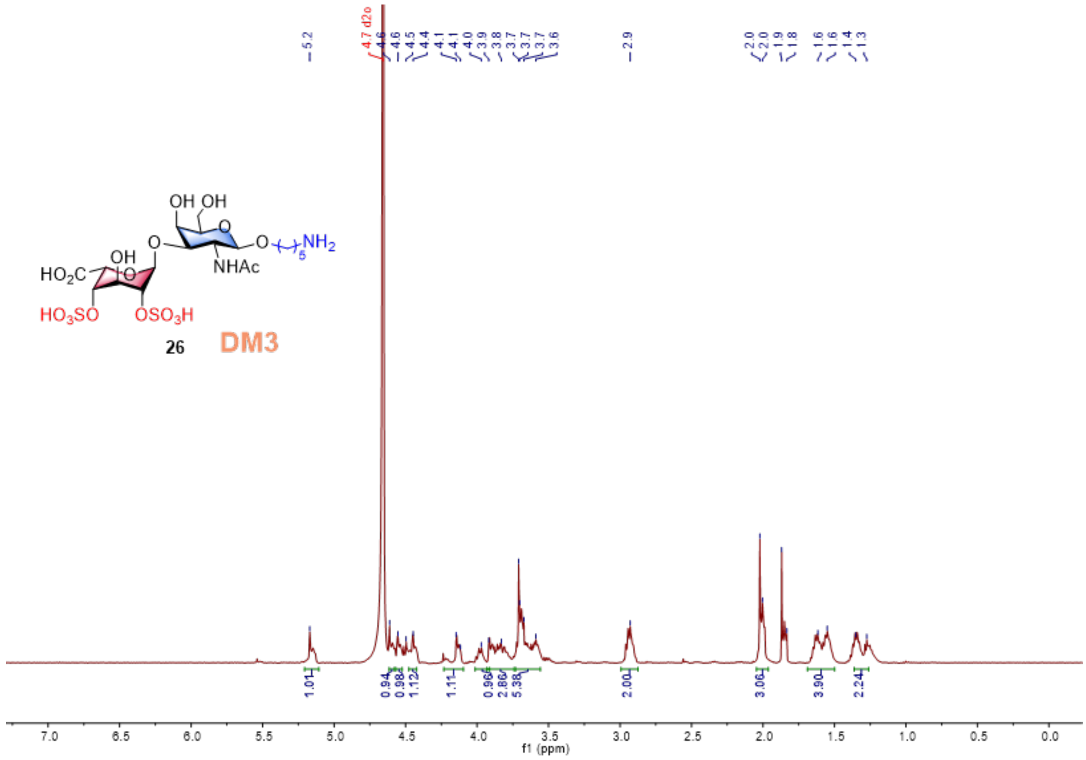

^{13}C NMR spectrum (D_2O, 126 MHz) of compound 26 (DM3)

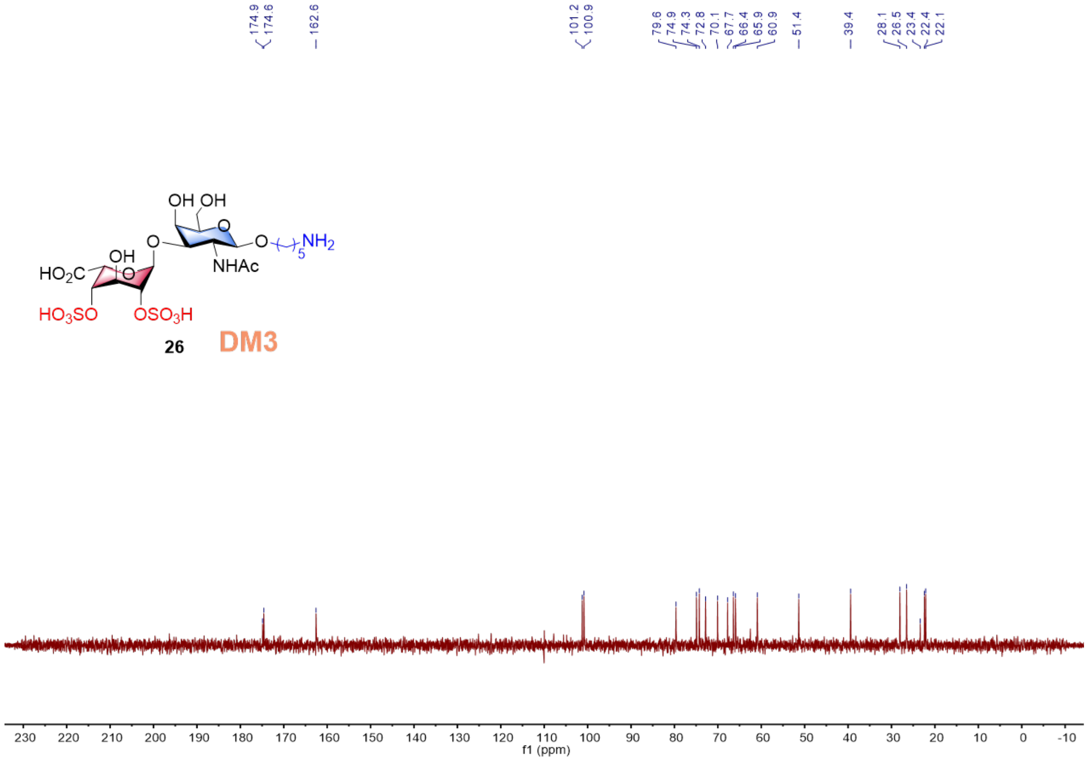

^{1}H NMR spectrum (CD_3OD, 500 MHz) of compound 27

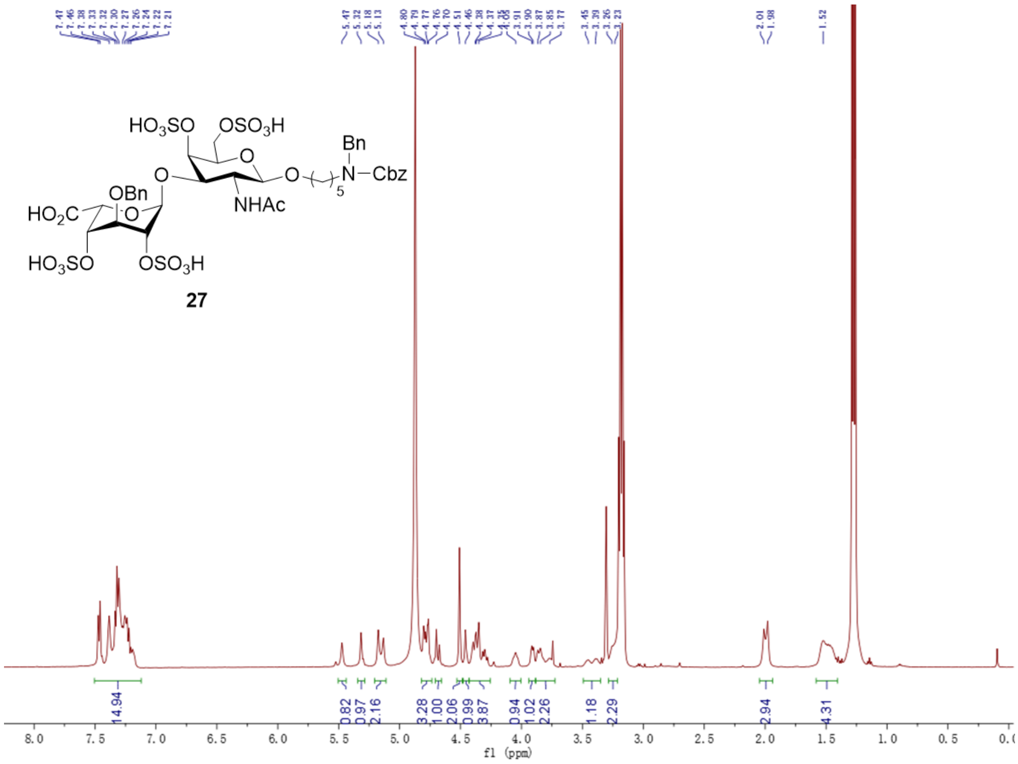

^{13}C NMR spectrum (CD_3OD, 126 MHz) of compound 27

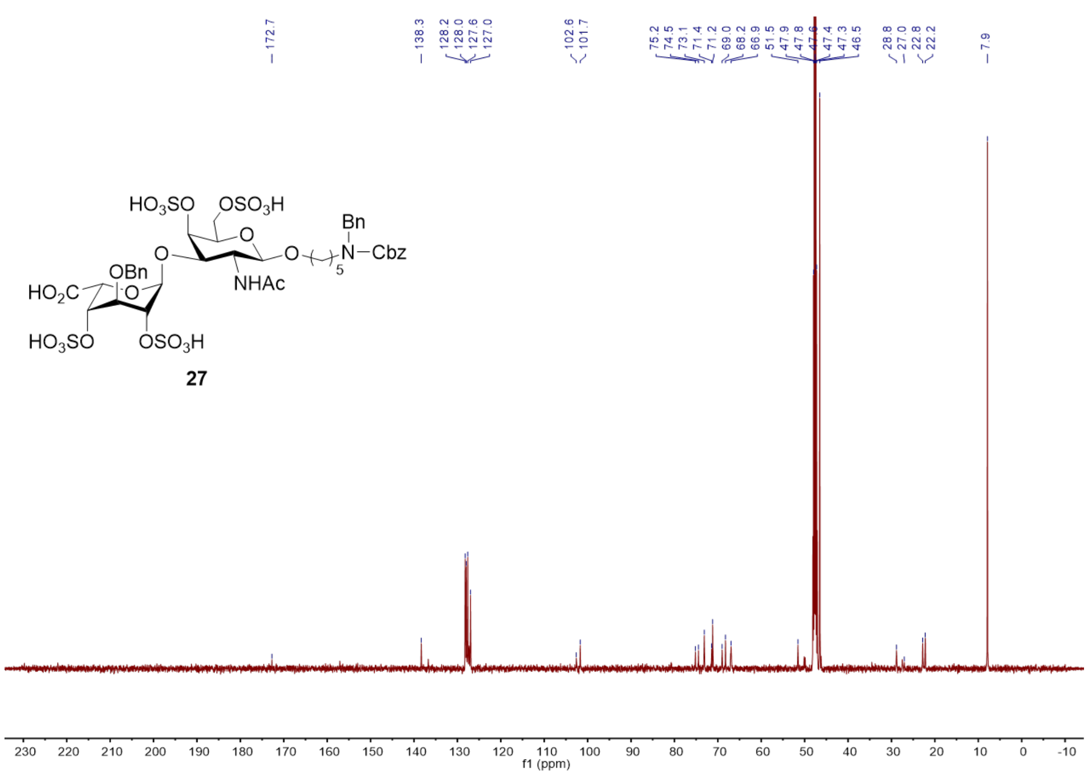

^{1}H-^{1}H COSY NMR spectrum (CD_3OD, 500 MHz) of compound 27

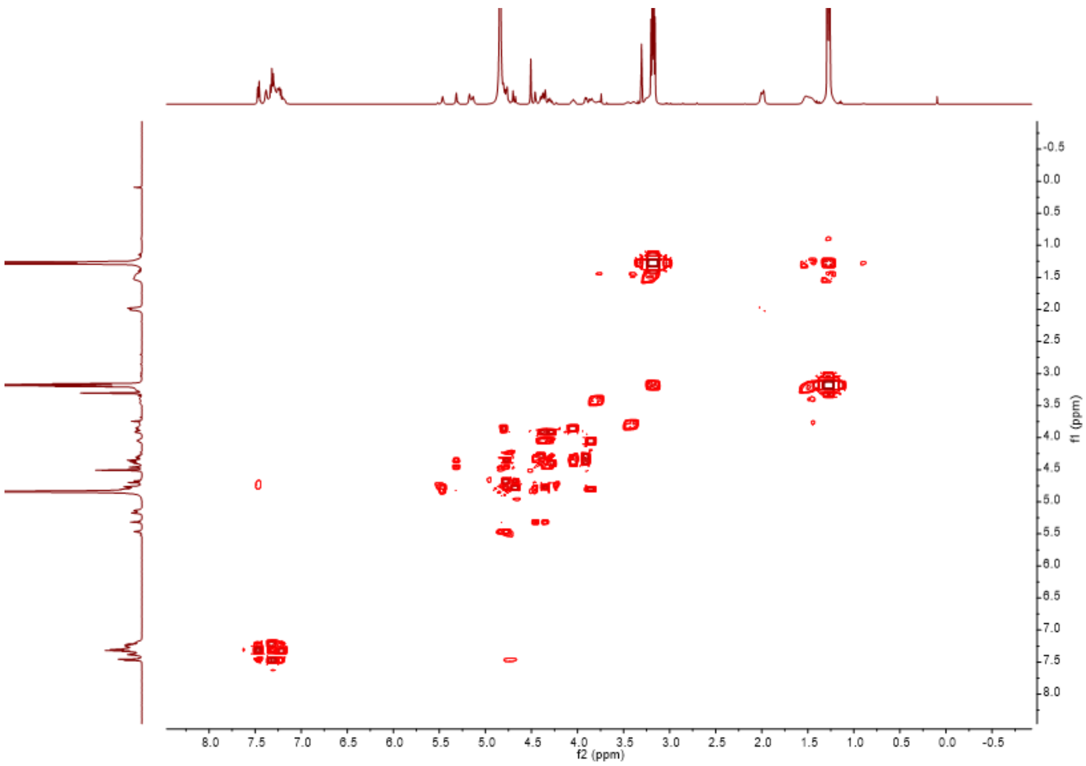

^{1}H-^{13}C HSQC NMR spectrum (CD_3OD, 500 MHz) of compound 27

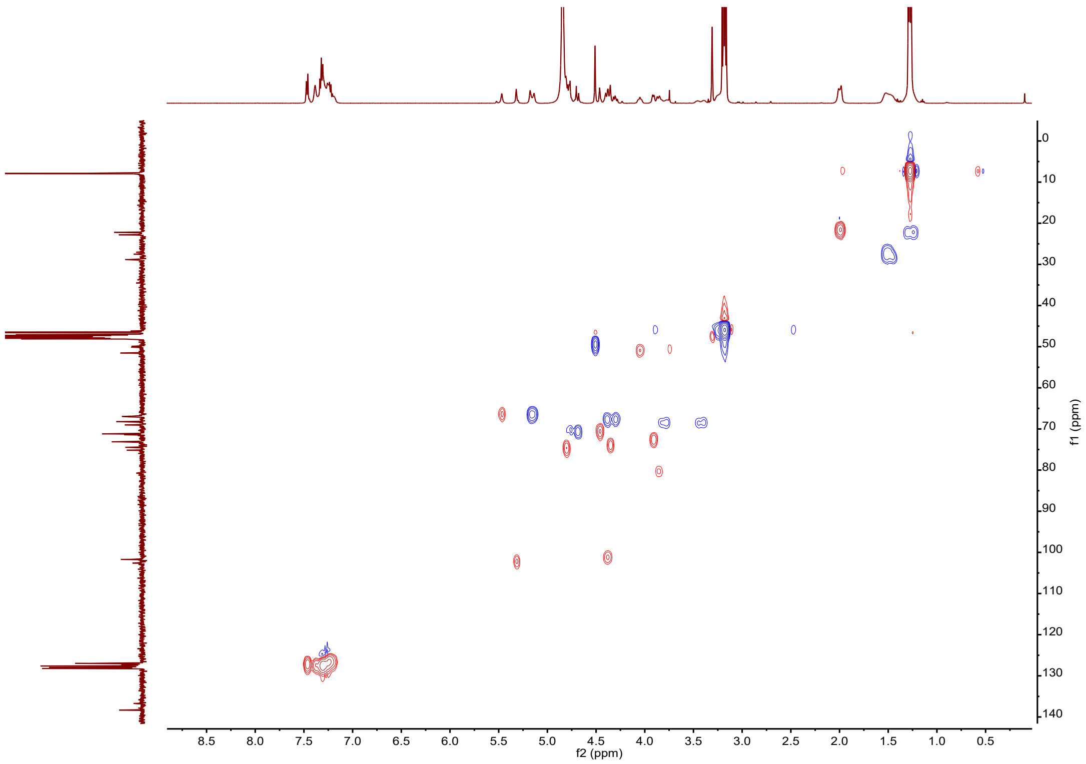

^{1}H NMR spectrum (D_2O, 500 MHz) of compound 28 (DM4)

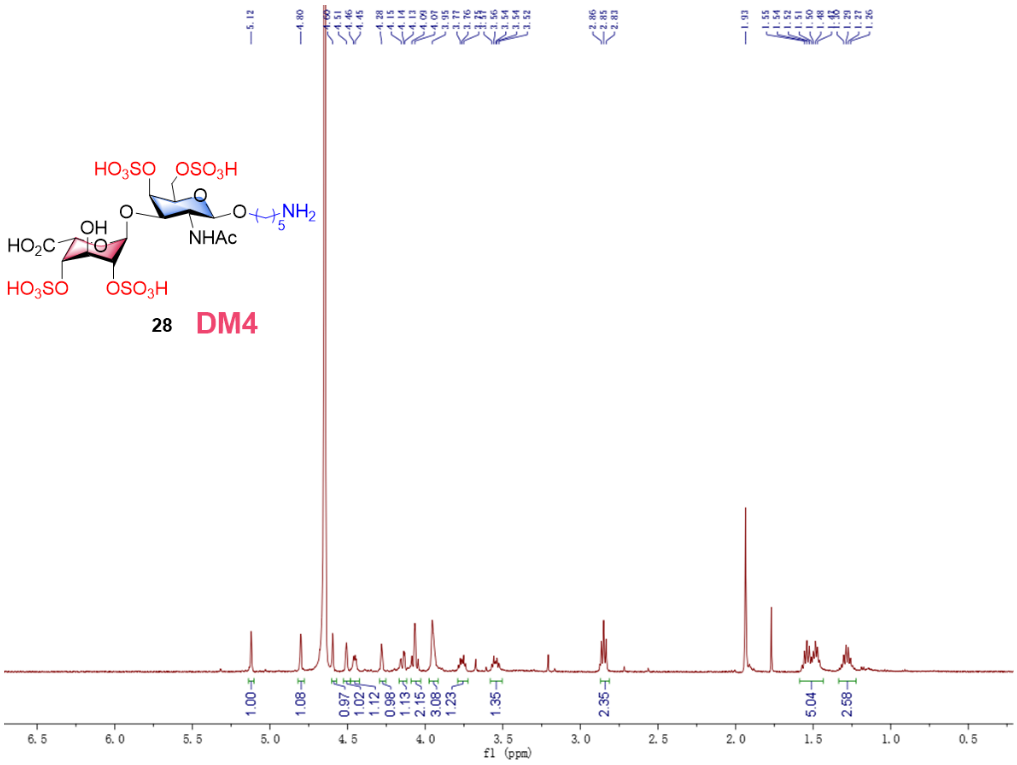

^{13}C NMR spectrum (D_2O, 126 MHz) of compound 28 (DM4)

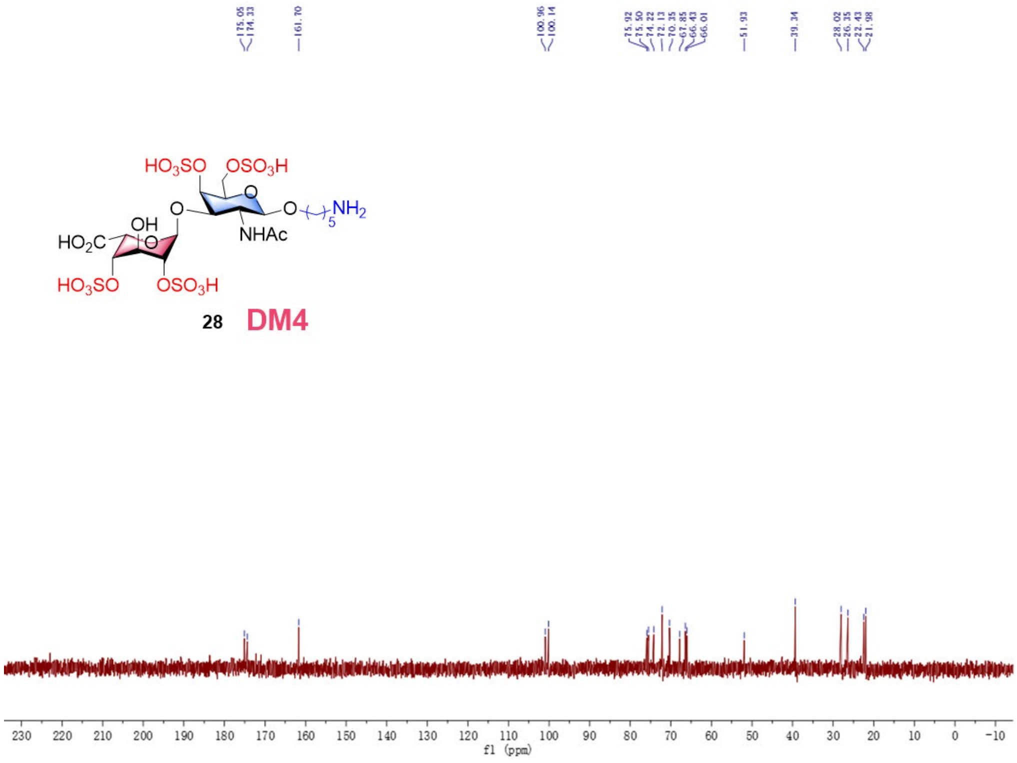

^1H-^1H COSY NMR spectrum (D_2O, 500 MHz) of compound 28 (DM4)

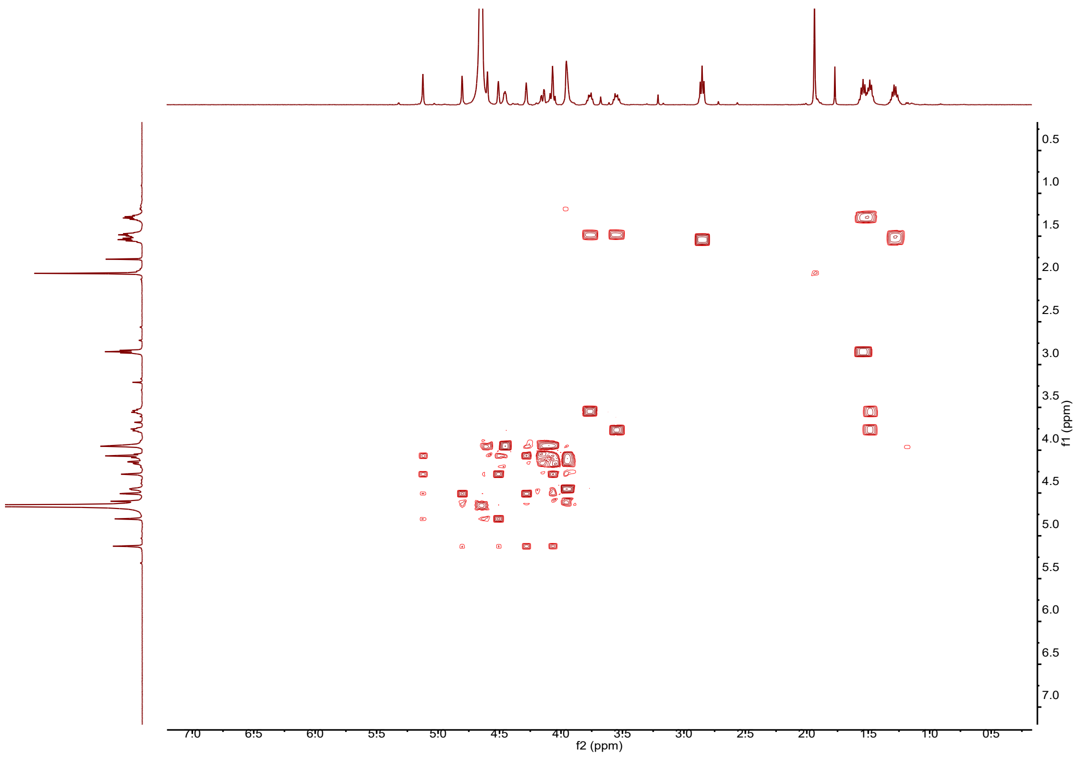

^1H-^{13}C HSQC NMR spectrum (D_2O, 500 MHz) of compound 28 (DM4)

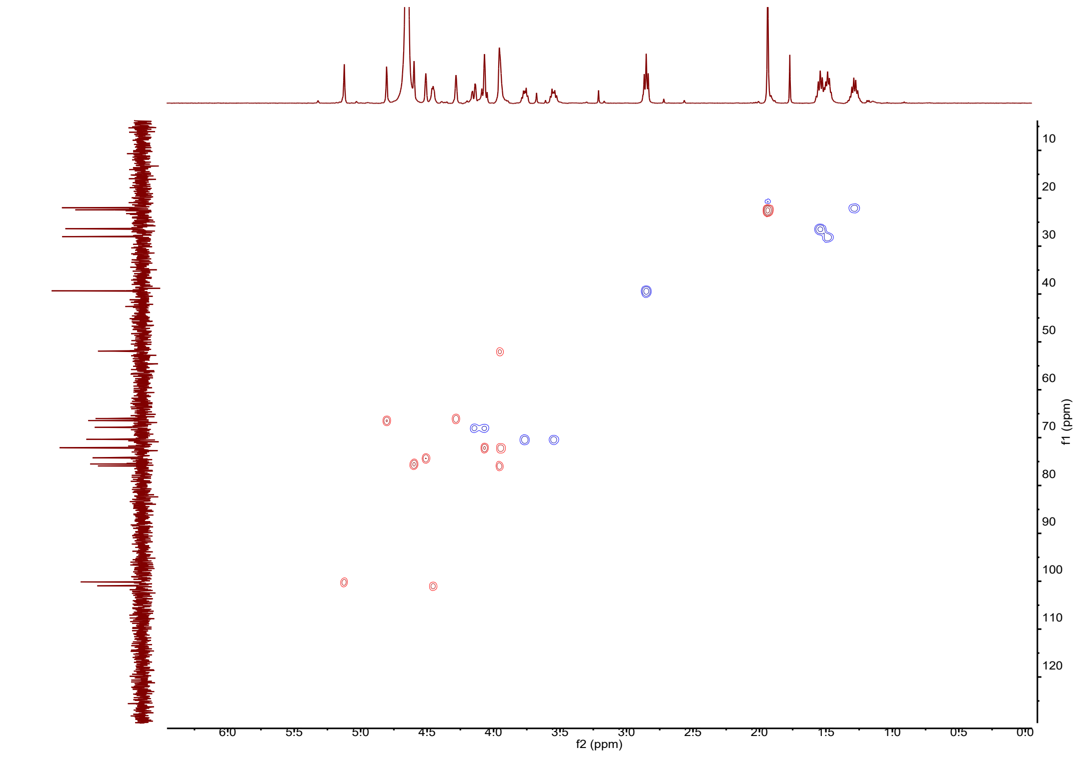

^{1}H-^{13}C HMBC NMR spectrum (D_2O, 500 MHz) of compound 28 (DM4)

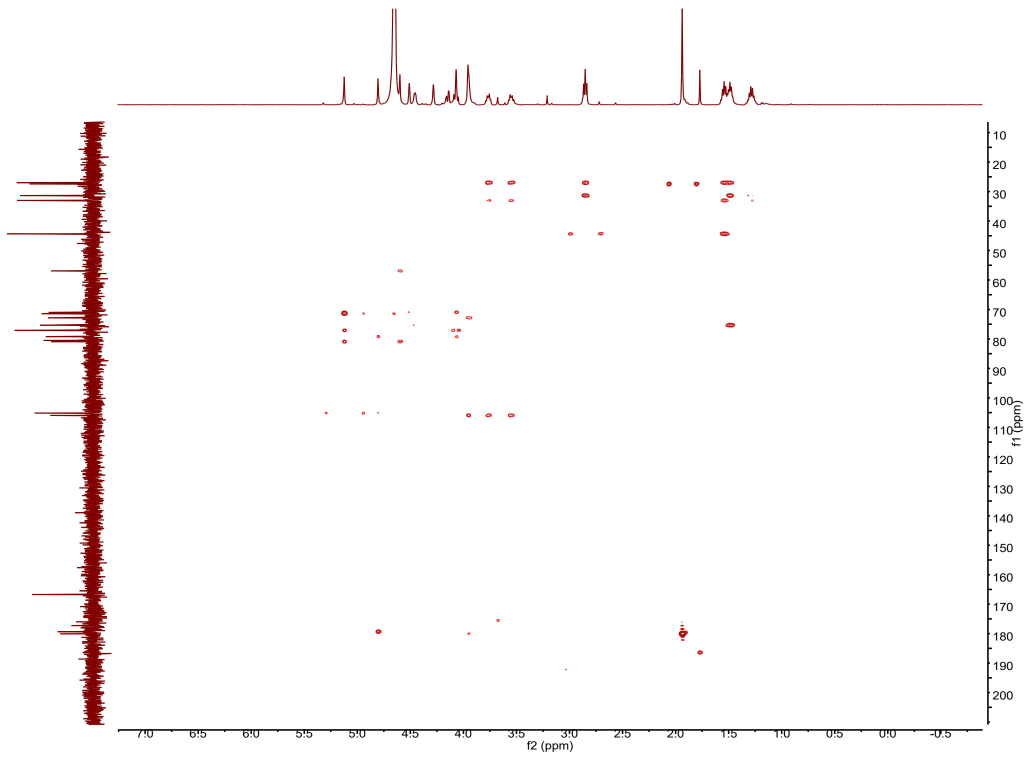

^{1}H NMR spectrum (DMF-d_7, 500 MHz) of compound polymer backbone 30

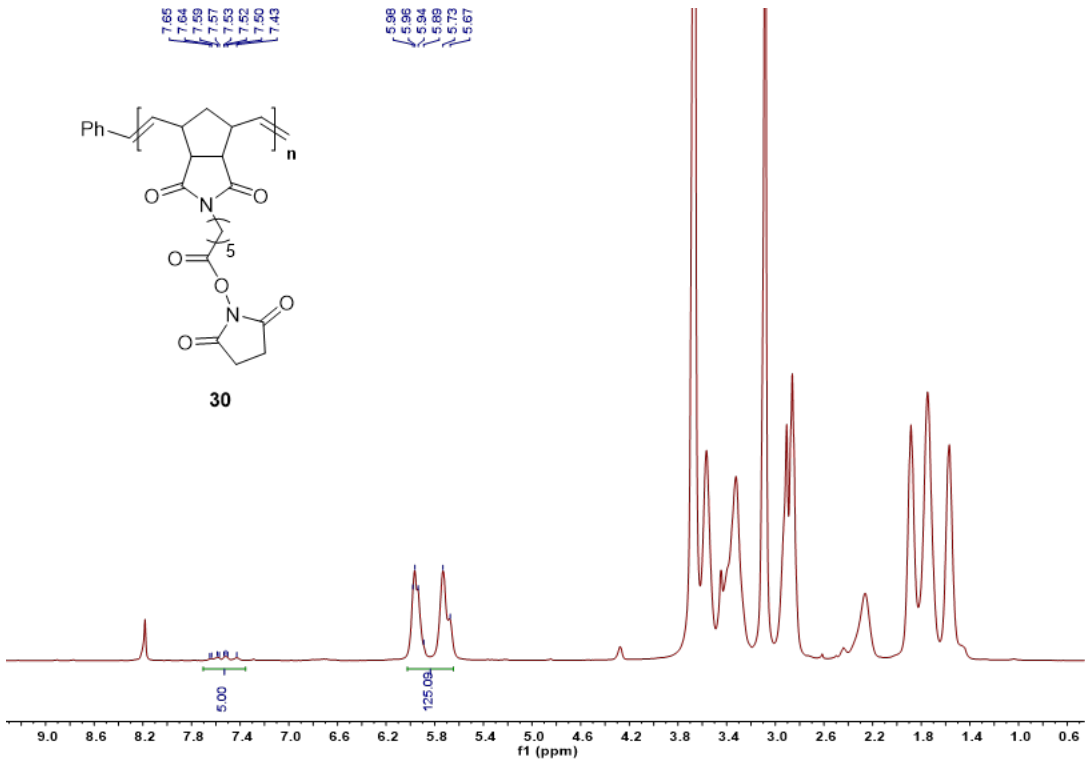

^1H NMR spectrum (D_2O, 500 MHz) of compound DMP1

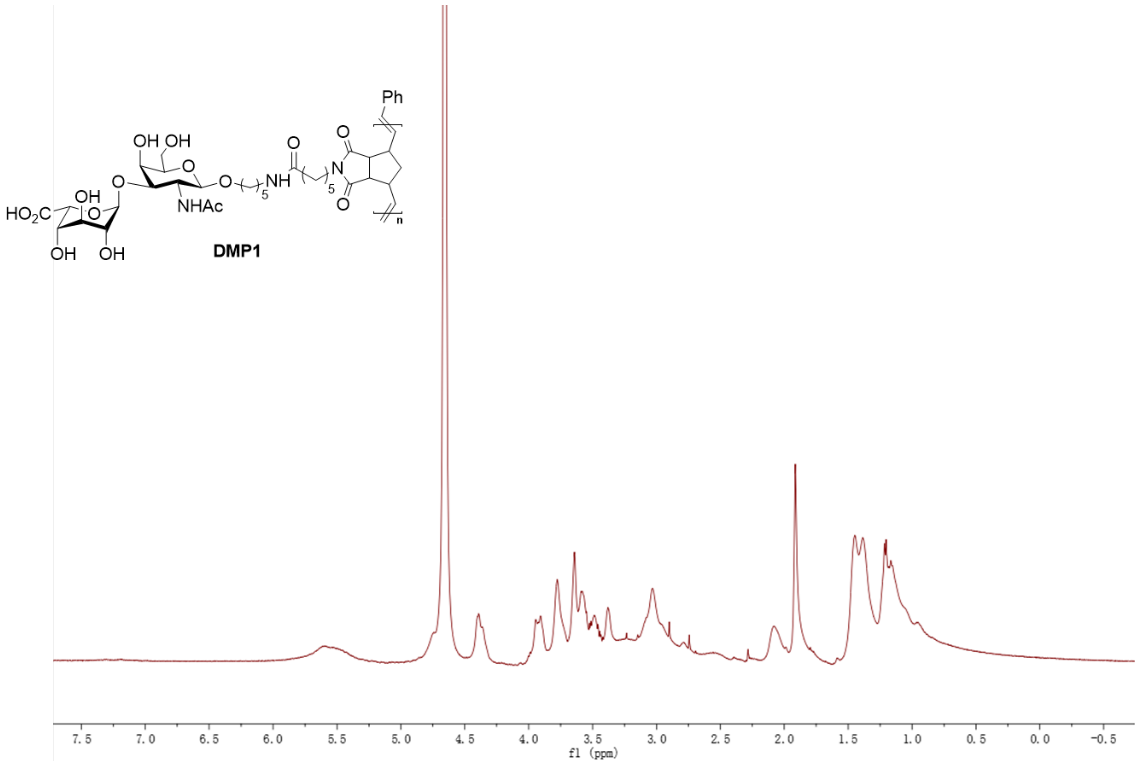

^1H NMR spectrum (D_2O, 500 MHz) of compound DMP2

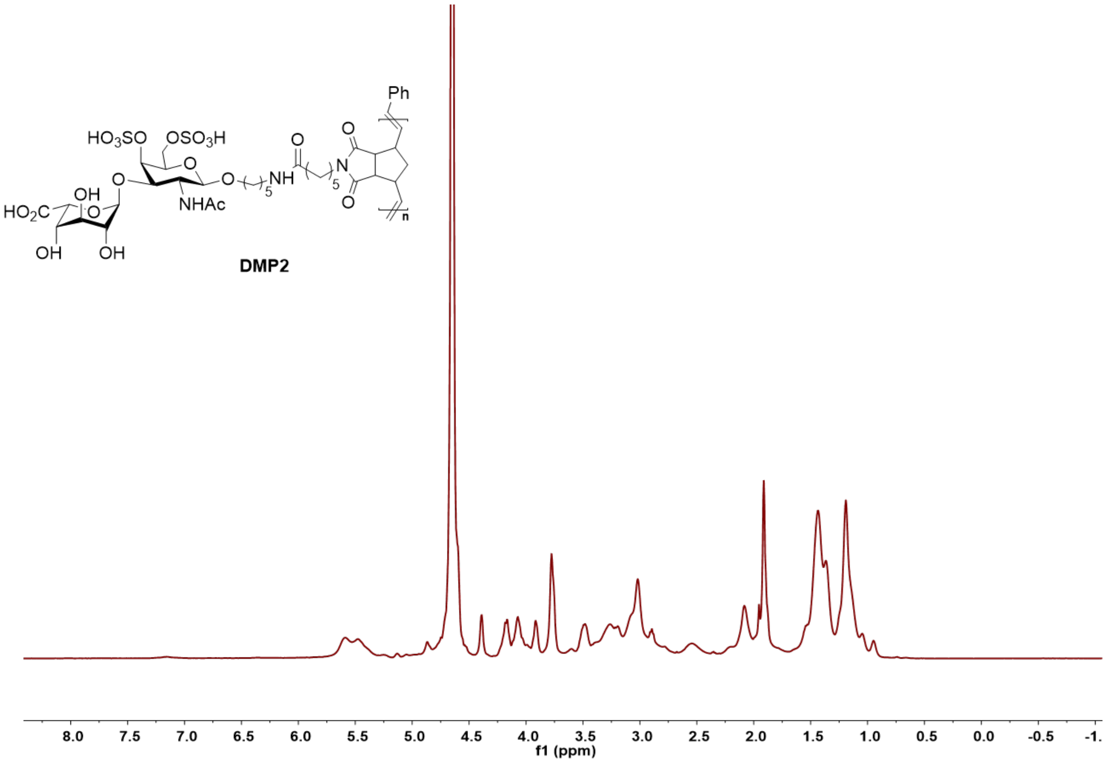

^1H NMR spectrum (D_2O, 500 MHz) of compound DMP3

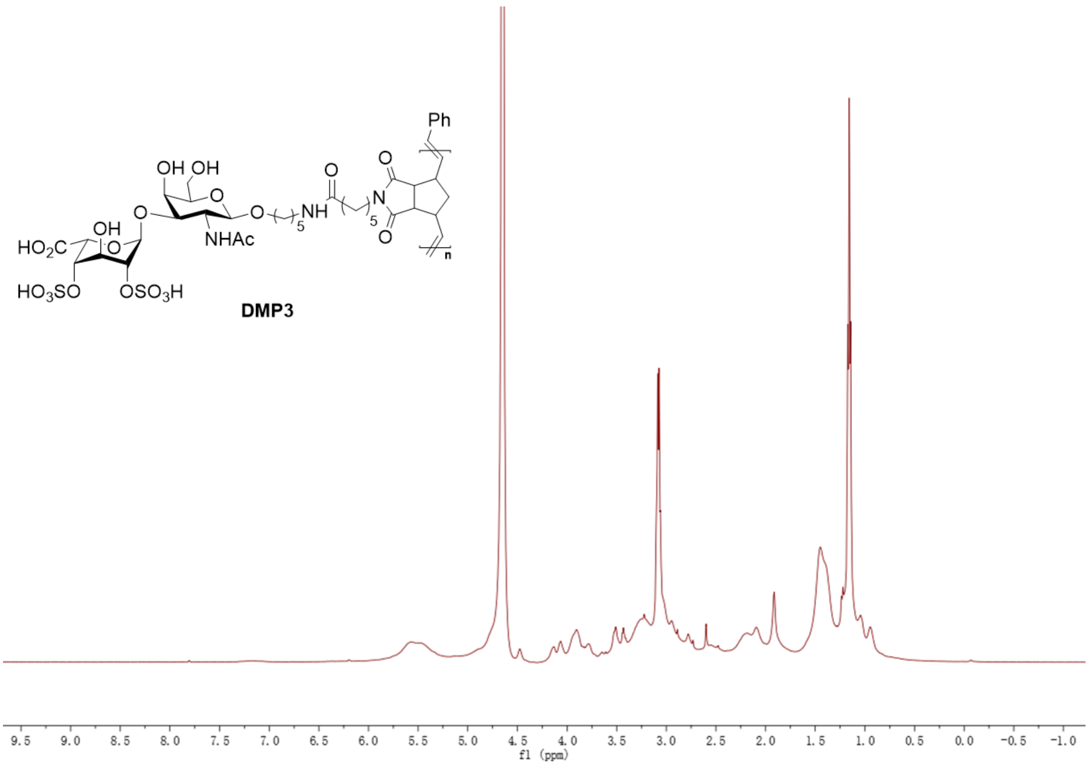

^{1}H NMR spectrum (D_2O, 500 MHz) of compound DMP4

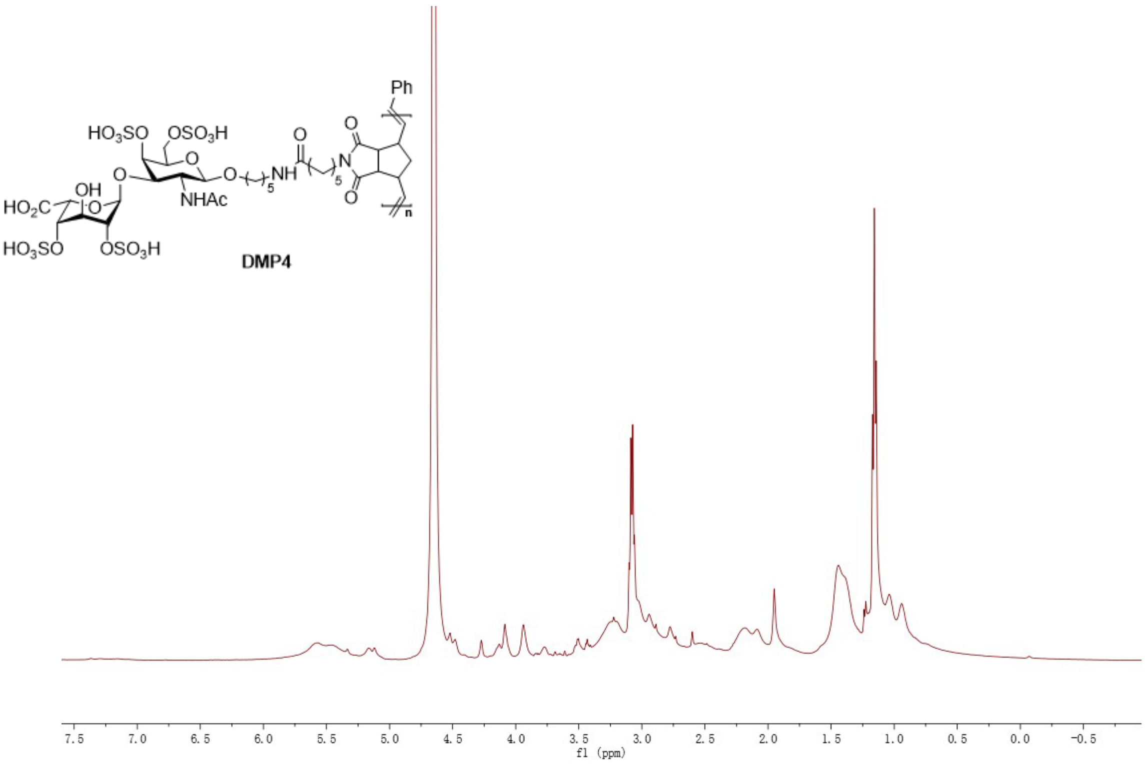

7. Transmission Electron Microscopy (TEM) Observations

7.1 TEM Imaging of the Glycopolymers

For TEM observation of the glycopolymer samples, the lyophilized sample powders were dissolved in PBS and filtered to make a 1 mg·mL^{-1} glycopolymer solution, sealed and left at room temperature overnight to allow the glycopolymer to self-assemble. Afterwards, a 20 μL aliquot of glycopolymer solution was dropped onto a 200 mesh copper TEM grids, stained with uranylacetate and dried before TEM observation.

7.2 TEM Imaging of the SARS-CoV-2 Pseudovirus.

Two hours prior to the TEM observation of SARS-CoV-2 pseudovirus aliquot (1.76 ×10^{6} TU·mL^{-1}) was thawed slowly on ice and then co-incubated with 1 mg·mL^{-1} DMP4 solution or PBS buffer (10 mM pH 7.4) at 37 °C for 5 min or 2 h before TEM imaging. Afterwards, a 20 μL of mixture was dropped onto a 200-mesh copper TEM grids, stained with uranylacetate and dried before TEM observation.

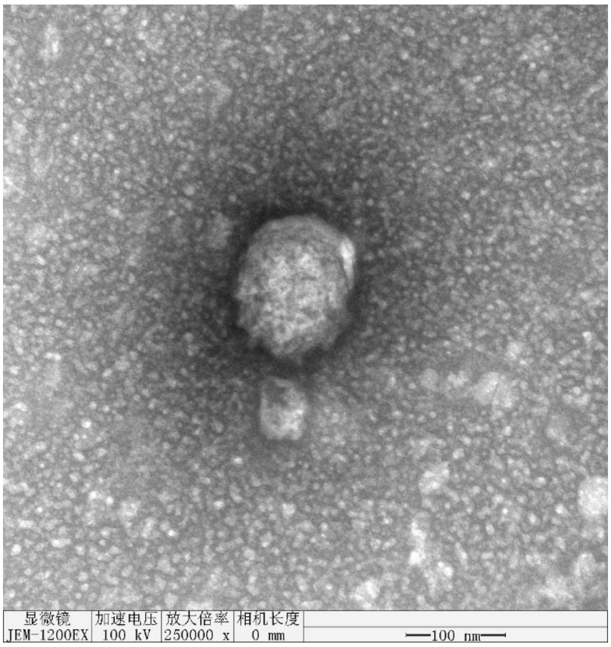

Figure S7. TEM images of pseudovirus

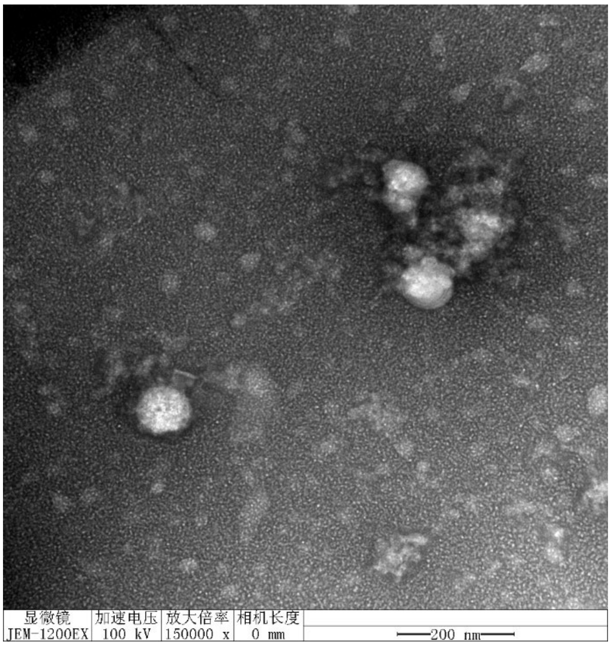

Figure S8. TEM images of co-incubation of the pseudovirus with **DMP4** for 5 minutes at 37 °C.

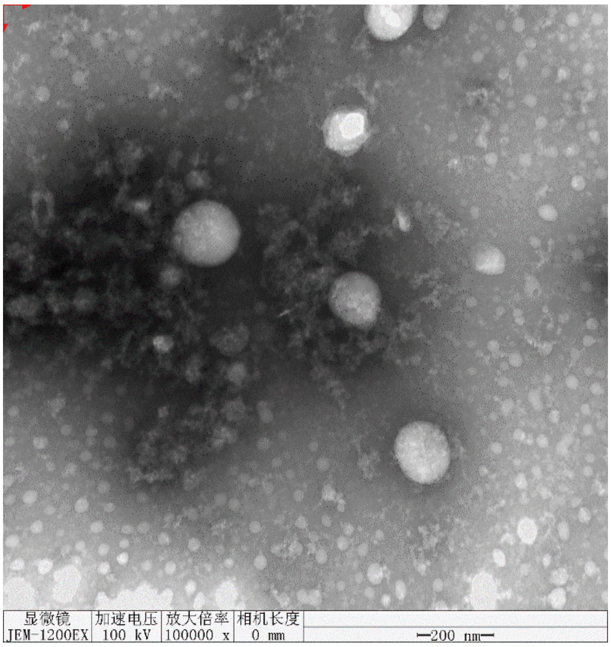

Figure S9. TEM images of co-incubation of the pseudovirus with **DMP4** for 2 hours at 37 °C.

8. Dynamic Light Scattering (DLS) and Zeta potential

For DLS and ζ-potential experiments of the glycopolymer samples, the lyophilized sample powders were dissolved in 10 mM PBS buffer (pH 7.4) and filtered to make a 1 $mg{\cdot}mL^{-1}$ glycopolymer solution, sealed and left at room temperature overnight to allow the glycopolymer to self-assemble. The results of the DLS and ζ-potential experiments were determined with a Malvern Nano ZS 90 nanoparticle analyser (United Kingdom) at room temperature.

9. SARS-CoV-2 M^{pro} Protein Expression and Purification.

The BL21/pGEX4T1-M^{pro} strain was generously provided by Prof. Xin Wang (Ocean University of China). Protein purification was carried out following established procedures described in previous studies.[53] A GST-tagged recombinant protein was purified by a GST Fusion Protein Purification Kit (Beyotime, Cat No. P2262) and digested with Factor Xa Protease (NEB, Cat No. P8010S) to remove any GST tags to afford the desired M^{pro} protein. The purity of protein was approximately 98% as assessed by SDS-PAGE (Fig. S1). The catalytic activity of M^{pro} was measured by continuous kinetic assays , using an identical fluorogenic substrate Dabcyl-KTSAVLQSGFRKME-Edans (Beyotime, Cat No. P9733). Fluorescence intensity (excitation 340 nm, emission 490 nm) was monitored with microplate reader Tecan Spark 10M.

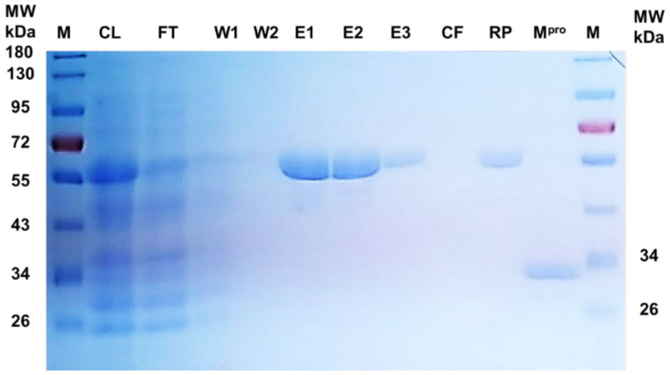

Figure S10. SDS-PAGE analysis of affinity chromatography-purified protein. Proteins (from left to right: Marker, Cell Lysate Solution, Flow Through Solution, Wash Solution 1-2, Elution Solution 1-3, Centrifugal Filtrates, Recombinant Protein, the desired M^{pro} protein, Marker) were successively resolved on a precasted 10% SDS-PAGE gel. The gel was stained with Coomassie Brilliant Blue. The migration positions of protein ladder (from Thermo Fisher Scientific) are indicated. The apparent mass of M^{pro} was approximately 33600 Da. Molecular mass standards (top to bottom: 180, 130, 95, 72, 55, 43, 34 and 26 kDa)

10. Confocal Microscopy for Internalization of Different Cells.

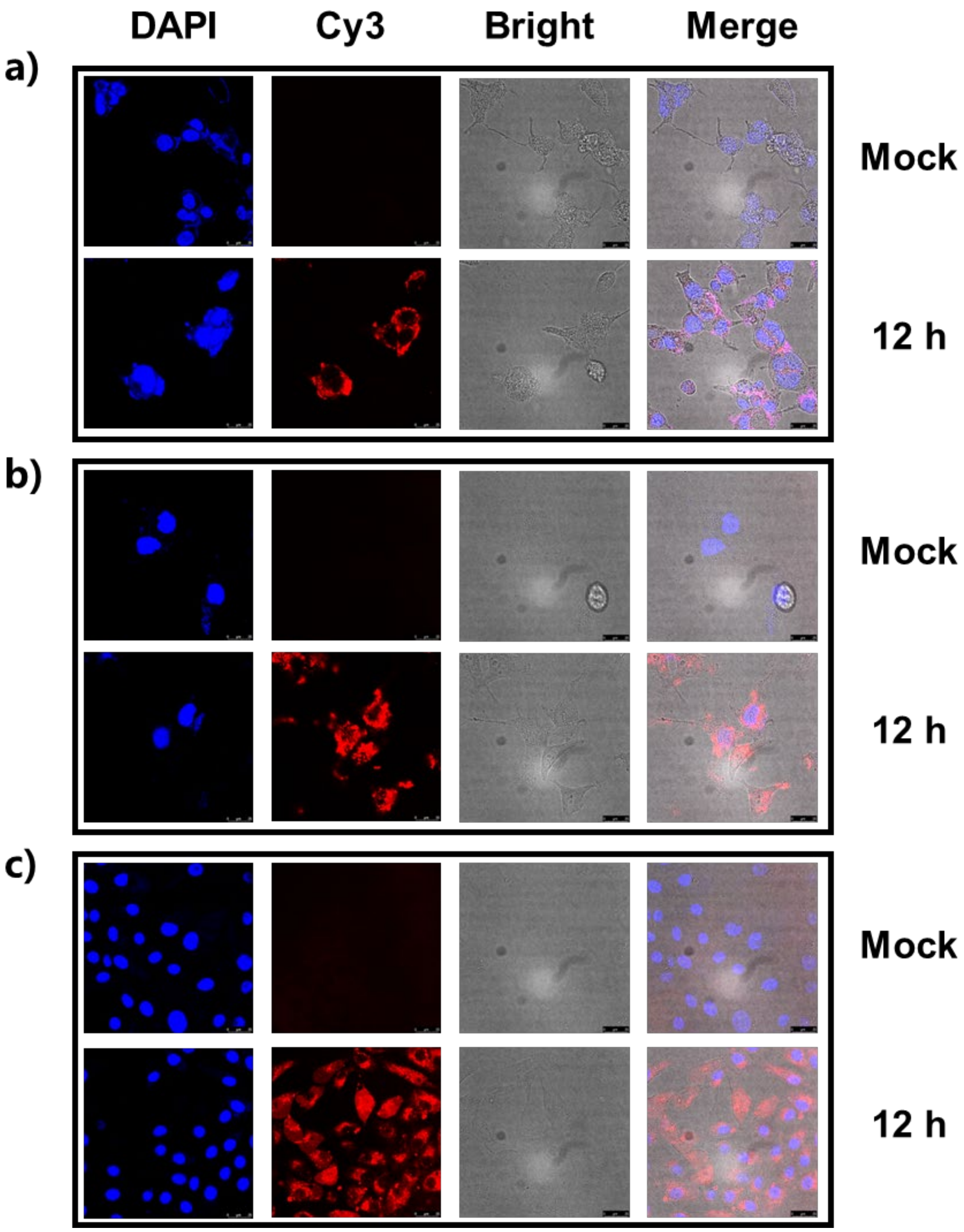

Figure S11. Confocal images of cells incubated with Cy3-SS (20 μg·mL−1) for 12 h: (a) HEK-293T cells; (b) Hela cells; (c) Vero cells.

References

[40] O. C. Grant, D. Wentworth, S. G. Holmes, R. Kandel, D. Sehnal, X. Wang, Y. Xiao, P. Sheppard, T. Grelsson, A. Coulter, G. Miller, B. L. Foley, R. J. Woods, Generating 3D models of carbohydrates with GLYCAM-web *Biorxiv* **2025**, 2025.5.8.652828.

[41] A. K. Nivedha, D. F. Thieker, H. Hu, R. J. Woods, Vina-carb: Improving glycosidic angles during carbohydrate docking *J. Chem. Theory Comput.* **2016**, *12*, 892–901.

[46] G. J. S. Lohman, D. K. Hunt, J. A. Högermeier, P. H. Seeberger, Synthesis of Iduronic Acid Building Blocks for the Modular Assembly of Glycosaminoglycans *J. Org. Chem.* **2003**, *68*, 7559–7561.

[39] C. Yang, L. Gao, M. Shao, C. Cai, L. Wang, Y. Chen, J. Li, F. Fan, Y. Han, M. Liu, R. J. Linhardt, G. Yu, End-functionalised glycopolymers as glycosaminoglycan mimetics inhibit HeLa cell proliferation *Polym. Chem.* **2020**, *11*, 4714–4722.

[54] F. Kong, Recent studies on reaction pathways and applications of sugar orthoesters in synthesis of oligosaccharides *Carbohydr. Res.* **2007**, *342*, 345–373.

[55] R. Castelli, H. S. Overkleeft, G. A. van der Marel, J. D. C. Codée, 2,2-dimethyl-4-(4-methoxy-phenoxy) butanoate and 2,2-dimethyl-4-azido butanoate: Two new pivaloate-ester-like protecting groups *Org. Lett.* **2013**, *15*, 2270–2273.

[53] Z. Li, X. Li, Y.-Y. Huang, Y. Wu, R. Liu, L. Zhou, Y. Lin, D. Wu, L. Zhang, H. Liu, X. Xu, K. Yu, Y. Zhang, J. Cui, C.-G. Zhan, X. Wang, H.-B. Luo, Identify potent SARS-CoV-2 main protease inhibitors via accelerated free energy perturbation-based virtual screening of existing drugs *Proc. Natl. Acad. Sci.* **2020**, *117*, 27381–27387.
